# Supplementary material for: Repurposing FDA-approved phytomedicines, natural products, antivirals and cell protectives against SARS-CoV-2 (COVID-19) RNA-dependent RNA polymerase
Source: PeerJ. 2020 Nov 30;8:e10480. doi: 10.7717/peerj.10480 (PMC7713599; doi:10.7717/peerj.10480)
Supplement: Supplemental Information 1 — The compounds are ramked according to their docking scores. [file peerj-08-10480-s001.pdf]

Name	MolWeight	CAS	docking score	glide ligand efficiency	glide lipo	glide hbond	glide evdw
NAD+	663.4	53-84-9	-8.2	-0.2	-0.9	-0.5	-56.2
Sennoside B	862.7	128-57-4	-8.1	-0.1	-0.6	-0.2	-58.8
Leucovorin Calcium Pentahydrate	601.6	6035-45-6	-8.1	-0.2	-0.3	-1.5	-32.2
Thymopentin	679.8	69558-55-0	-7.9	-0.2	-1.6	-1.5	-50.3
Digoxin	780.9	20830-75-5	-7.8	-0.1	-2.4	-0.2	-62.4
Ritonavir	720.9	155213-67-5	-7.7	-0.2	-2.9	-0.3	-67.9
Asiaticoside	959.1	16830-15-2	-7.6	-0.1	-1.8	0.0	-50.4
Glycyrrhizin (Glycyrrhizic Acid)	822.9	1405-86-3	-7.6	-0.1	-0.8	0.0	-55.6
Neohesperidin dihydrochalcone (Nhdc)	612.6	20702-77-6	-7.5	-0.2	-1.4	-0.1	-46.7
Venetoclax (ABT-199, GDC-0199)	868.4	1257044-40-8	-7.5	-0.1	-2.5	-0.6	-64.0
Taxifolin (Dihydroquercetin)	304.3	480-18-2	-7.4	-0.3	-1.3	-0.4	-23.7
Oxiglutatione	612.6	27025-41-8	-7.4	-0.2	-0.8	-0.8	-48.2
Iopamidol	777.1	60166-93-0	-7.4	-0.2	-0.9	-1.1	-37.1
(-)-Epicatechin gallate	442.4	1257-08-5	-7.3	-0.2	-1.3	-0.4	-31.8
Acarbose	645.6	56180-94-0	-7.3	-0.2	-1.2	-0.3	-37.2
Chlorhexidine HCl	578.4	3697-42-5	-7.3	-0.2	-1.9	-0.4	-39.7
Salvianolic acid B	718.6	121521-90-2	-7.3	-0.1	-1.2	0.0	-55.3
Sennoside A	862.7	81-27-6	-7.2	-0.1	-0.8	0.0	-45.6
Troxerutin	742.7	7085-55-4	-7.2	-0.1	-1.0	-0.5	-42.1
Cefodizime Sodium	628.6	86329-79-5	-7.2	-0.2	-1.0	-0.9	-44.0
(-)Epicatechin	290.3	490-46-0	-7.1	-0.3	-1.0	-0.6	-22.0
Ioversol	807.1	87771-40-2	-7.1	-0.2	-0.7	-1.1	-38.1
Quercetin (Sophoretin)	302.2	117-39-5	-7.1	-0.3	-1.1	-0.4	-25.1
Quercetin dihydrate (Sophoretin)	338.3	6151-25-3	-7.1	-0.3	-1.1	-0.4	-25.1
Pyrantel Pamoate	594.7	22204-24-6	-7.1	-0.2	-1.9	-0.2	-34.4
Cobicistat (GS-9350)	776.0	1004316-88-4	-7.0	-0.1	-2.8	-0.4	-63.7
Aloin (Barbaloin)	418.4	1415-73-2	-7.0	-0.2	-1.5	-0.3	-28.3
Clofarabine	303.677	123318-82-1	-6.969	-0.348	-0.332	-0.428	-26.543
Bergenin (Cuscutin)	328.271	477-90-7	-6.948	-0.302	-1.16	-0.32	-20.808
Neomycin sulfate	712.722	1405-10-3	-6.941	-0.165	-0.582	-0.57	-38.273
Mizoribine (Bredinin)	259.216	50924-49-7	-6.933	-0.385	-0.8	-0.635	-20.257
Temozolomide	194.151	85622-93-1	-6.917	-0.494	0	-1.219	-24.981
Catalpol	362.329	2415-24-9	-6.906	-0.276	-1.247	0	-23.646
Protirelin	362.384	24305-27-9	-6.89	-0.265	-0.851	-1.237	-31.101

Neohesperidin	610.561	13241-33-3	-6.88	-0.16	-1.264	-0.371	-38.025
Cyclo(RGDyK)	849.733	250612-42-1	-6.863	-0.156	-0.894	-1.331	-45.493
Chlorthalidone	338.766	77-36-1	-6.859	-0.312	-1.098	-0.906	-30.898
Diammonium Glycyrrhizinate	856.993	79165-06-3	-6.858	-0.118	-0.304	-0.067	-48.962
Resveratrol	228.243	501-36-0	-6.849	-0.403	-1.4	-0.72	-19.559
Isoquercitrin	464.376	482-35-9	-6.83	-0.207	-1.551	-0.32	-37.25
Echinacoside	786.728	82854-37-3	-6.819	-0.124	-1.249	0	-45.529
Isoprinosine	1115.233	36703-88-5	-6.809	-0.358	-0.717	-0.48	-16.47
Inosine	268.226	58-63-9	-6.808	-0.358	-0.717	-0.48	-16.479
Rutin (Rutoside)	610.518	153-18-4	-6.803	-0.158	-0.871	0	-40.521
Luteolin	286.236	491-70-3	-6.761	-0.322	-1.057	-0.304	-20.317
bentiromide	404.415	37106-97-1	-6.76	-0.225	-1.645	-0.434	-35.623
ceftazidime pentahydrate	636.652	78439-06-2	-6.752	-0.182	-1.278	-0.515	-43.42
Oleuropein	540.514	32619-42-4	-6.725	-0.177	-1.105	-0.248	-35.154
Lenalidomide (CC-5013)	259.261	191732-72-6	-6.723	-0.354	-0.837	-0.658	-30.929
Pralatrexate(Folotyn)	477.473	146464-95-1	-6.701	-0.191	-0.849	-1.182	-37.38
Kaempferol	286.236	520-18-3	-6.669	-0.318	-1.087	-0.652	-23.217
Nilotinib (AMN-107)	529.516	641571-10-0	-6.657	-0.171	-2.517	-0.32	-53.458
Dichlorphenamide (Diclofenamide)	305.159	120-97-8	-6.638	-0.415	-0.458	-0.77	-24.707
Didanosine (Videx)	236.227	69655-05-6	-6.624	-0.39	-0.556	-0.565	-24.646
Novobiocin sodium (Albamycin)	634.606	1476-53-5	-6.59	-0.15	-1.772	-0.415	-45.099
Diacerein	368.294	13739-02-1	-6.588	-0.244	-1.262	-0.468	-34.361
Liquiritin	418.394	551-15-5	-6.583	-0.219	-1.146	-0.205	-30.824
Doxifluridine	246.192	436349	-6.577	-0.387	-0.506	-0.617	-23.126
Bismuth Subcitrate Potassium	782.671	880149-29-1	-6.572	-0.506	0	-0.825	-8.971
Morin hydrate (Aurantica)	320.251	6202-27-3	-6.57	-0.299	-1.058	-0.577	-29.029
Notoginsenoside R1	933.127	80418-24-2	-6.563	-0.101	-0.98	0	-43.573
Ammonium Glycyrrhizinate (AMGZ)	839.963	1407-03-0	-6.545	-0.113	-0.264	-0.32	-51.998
Ceftiofur hydrochloride	560.024	103980-44-5	-6.543	-0.192	-1.503	-0.466	-53.992
Guanosine	283.241	118-00-3	-6.525	-0.326	-0.832	-0.302	-27.699
Cilengitide trifluoroacetate	702.679	188968-51-6	-6.522	-0.155	-1.205	-0.847	-37.518
Clodronate Disodium	288.856	22560-50-5, 1059	-6.504	-0.591	0	-0.84	-9.987
5-hydroxytryptophan (5-HTP)	220.225	56-69-9	-6.503	-0.406	-1.251	-0.633	-19.379
Zileuton	236.29	111406-87-2	-6.501	-0.406	-1.289	-0.31	-20.333
Folic acid	441.397	59-30-3	-6.499	-0.203	-1.111	-0.683	-40.469

Deferasirox (Exjade)	373.361	201530-41-8	-6.482	-0.232	-0.743	-0.626	-34.917
Diosmin	608.545	520-27-4	-6.481	-0.151	-0.819	-0.088	-35.363
spiramycin	843.053	8025-81-8	-6.472	-0.11	-2.087	0	-49.59
Lomitapide	693.72	182431-12-5	-6.472	-0.129	-2.329	0	-55.594
Streptomycin sulfate	1457.384	3810-74-0	-6.44	-0.161	-0.935	-0.731	-37.34
Hesperidin	610.561	520-26-3	-6.437	-0.15	-0.877	-0.072	-34.533
Apigenin	270.237	520-36-5	-6.433	-0.322	-1.103	-0.659	-20.641
Cytarabine	243.217	147-94-4	-6.428	-0.378	-0.491	-0.602	-20.909
Fludarabine Phosphate (Fludara)	365.212	75607-67-9	-6.425	-0.268	-0.339	-0.411	-29.855
Arbutin (Uva, p-Arbutin)	272.251	497-76-7	-6.424	-0.338	-0.967	-0.32	-18.986
R935788 (Fostamatinib disodium)	624.423	1025687-58-4	-6.399	-0.16	-1.438	-0.767	-43.207
Thiamine HCl (Vitamin B1)	337.269	24539	-6.396	-0.355	-0.851	-0.999	-26.041
Ribostamycin Sulfate	552.551	53797-35-6	-6.394	-0.206	-1.024	-0.99	-30.343
Cefoselis sulfate	620.636	122841-12-7	-6.376	-0.182	-0.755	-1.038	-46.003
Aesculin (Esculin)	340.282	531-75-9	-6.371	-0.265	-1.514	-0.082	-29.695
Labetalol HCl	364.866	32780-64-6	-6.369	-0.265	-1.601	-0.749	-27.966
Entecavir hydrate	295.294	209216-23-9	-6.361	-0.318	-1.073	-0.637	-26.379
Sulfasalazine (Azulfidine)	398.393	599-79-1	-6.355	-0.227	-1.65	-0.208	-41.581
Cefdinir (Omnicef)	395.414	91832-40-5	-6.352	-0.244	-0.867	-0.53	-37.601
Ethacridine lactate monohydrate	361.392	6402-23-9	-6.335	-0.333	-2.189	-0.345	-29.684
Clevudine (Levovir)	260.219	163252-36-6	-6.324	-0.351	-0.925	-0.434	-24.409
Raltitrexed (Tomudex)	458.488	112887-68-0	-6.323	-0.198	-1.54	-0.16	-43.814
Dihydrostreptomycin sulfate	1461.415	5490-27-7	-6.321	-0.158	-0.532	-0.768	-28.8
Thioguanine	167.192	154-42-7	-6.313	-0.574	-0.259	-0.548	-22.361
(+)-Catechin	290.268	154-23-4	-6.312	-0.301	-1.162	-0.403	-26.291
Ixazomib Citrate (MLN9708)	517.122	1201902-80-8	-6.307	-0.186	-1.022	-0.425	-44.84
Daidzin	416.378	552-66-9	-6.306	-0.21	-1.852	-0.218	-29.398
Noradrenaline bitartrate monohydrate (Levoph	337.28	108341-18-0	-6.295	-0.525	-0.906	-0.65	-14.806
Naringin Dihydrochalcone (Naringin DC)	582.55	18916-17-1	-6.288	-0.153	-0.755	-0.452	-26.086
PD 0332991 (Palbociclib) Isethionate	573.664	827022-33-3, 571	-6.279	-0.19	-1.879	-0.369	-46.648
Vidarabine (Vira-A)	267.241	5536-17-4	-6.264	-0.33	-0.279	-0.083	-24.46
Methoxsalen (Oxsoralen)	216.19	298-81-7	-6.248	-0.391	-1.88	-0.351	-28.78
Doripenem Hydrate	438.52	364622-82-2	-6.245	-0.231	-1.281	-0.603	-34.147
Maltose	342.296	69-79-4	-6.238	-0.271	-1.035	-0.412	-21.456
Emodin	270.237	518-82-1	-6.233	-0.312	-1.332	-0.165	-30.848

Orotic acid (6-Carboxyuracil)	156.096	65-86-1	-6.232	-0.567	-0.323	-0.26	-17.835
Ginsenoside Re	947.154	52286-59-6	-6.231	-0.094	-0.531	-0.162	-38.639
Cytidine	243.217	65-46-3	-6.217	-0.366	-0.789	-0.16	-26.343
Sofosbuvir(PSI-7977)	529.453	1190307-88-0	-6.215	-0.173	-1.185	-0.686	-48.891
6-Mercaptopurine (6-MP) Monohydrate	170.192	6112-76-1	-6.212	-0.621	-0.226	-0.521	-21.159
Lisinopril (Zestril)	405.488	83915-83-7	-6.209	-0.214	-1.633	-0.541	-31.658
Mercaptopurine (6-MP)	152.177	50-44-2	-6.208	-0.621	-0.225	-0.521	-21.176
Danthron	240.211	117-10-2	-6.202	-0.345	-1.292	-0.152	-28.285
Piracetam	142.156	7491-74-9	-6.201	-0.62	-0.661	-0.481	-17.191
Salicin (Salicoside, Salicine)	286.278	138-52-3	-6.183	-0.309	-1.16	-0.16	-19.909
Radotinib	530.504	926037-48-1	-6.182	-0.159	-2.338	-0.32	-52.443
Hydralazine hydrochloride	196.637	304-20-1	-6.173	-0.514	-1.02	-0.355	-20.782
Montelukast Sodium	608.165	151767-02-1	-6.164	-0.15	-1.151	-0.935	-37.662
Cefadroxil hydrate	381.404	66592-87-8	-6.163	-0.247	-1.056	-0.146	-34.154
Cefixime	453.45	79350-37-1	-6.16	-0.205	-0.915	-0.107	-41.771
Puromycin 2HCl	544.431	58-58-2	-6.158	-0.181	-0.636	-0.236	-40.543
Atazanavir	704.855	198904-31-3	-6.156	-0.121	-2.101	-0.608	-50.39
Adenosine (Adenocard)	267.241	58-61-7	-6.144	-0.323	-0.604	-0.413	-25.29
Eslicarbazepine Acetate	296.321	236395-14-5	-6.137	-0.279	-1.56	-0.41	-30.18
Carbidopa	226.229	28860-95-9	-6.134	-0.383	-0.377	-0.354	-14.569
Trifluridine (Viroptic)	296.2	70-00-8	-6.132	-0.307	-0.66	-0.345	-26.102
Trimethoprim	290.318	738-70-5	-6.132	-0.292	-1.572	-0.611	-31.11
Valganciclovir Hydrochloride	392.839	175865-59-5	-6.127	-0.245	-0.419	-0.715	-37.704
Cefcapene Pivoxil Hydrochloride	622.111	147816-24-8	-6.117	-0.161	-0.481	-1.13	-36.911
Nepafenac	254.284	78281-72-8	-6.102	-0.321	-0.713	-1.179	-28.57
Scutellarin	462.36	27740-01-8	-6.102	-0.185	-1.421	0	-34.886
Lincomycin hydrochloride (Lincocin)	442.998	859-18-7	-6.097	-0.226	-0.79	-0.64	-21.891
Allantoin	158.115	97-59-6	-6.091	-0.554	0	-0.616	-20.944
Pemetrexed	471.374	150399-23-8	-6.087	-0.196	-1.006	-0.608	-30.7
Oclacitinib maleate	453.513	1640292-55-2	-6.086	-0.265	-1.375	-0.738	-30.471
Usnic acid	344.315	125-46-2	-6.079	-0.243	-1.066	-0.304	-27.292
Amikacin hydrate	603.618	1257517-67-1	-6.076	-0.152	-0.763	-0.28	-28.169
Gemcitabine HCl (Gemzar)	299.659	122111-03-9	-6.074	-0.337	-0.422	-0.531	-21.626
Peficitinib (ASP015K, JNJ-54781532)	326.393	944118-01-8	-6.066	-0.253	-1.451	-0.674	-31.348
Loganin	390.382	18524-94-2	-6.064	-0.225	-0.851	-0.247	-27.882

Mebendazole	295.293	31431-39-7	-6.06	-0.275	-0.883	-0.392	-29.976
Polydatin(Piceid)	390.384	65914-17-2	-6.057	-0.216	-0.706	-0.617	-24.762
Clorsulon	380.656	60200-06-8	-6.054	-0.303	-1.059	-0.946	-27.985
Equol	242.27	531-95-3	-6.053	-0.336	-1.543	-0.515	-27.06
Epinastine HCl	285.771	108929-04-0	-6.051	-0.318	-1.459	-0.387	-28.123
Zonisamide	212.226	68291-97-4	-6.048	-0.432	-0.987	-0.288	-23.049
Paromomycin Sulfate	713.707	1263-89-4	-6.042	-0.144	-0.757	-0.37	-40.248
Carzenide	201.2	138-41-0	-6.034	-0.464	-0.418	-0.457	-17.986
Cefoperazone (Cefobid)	645.667	62893-19-0	-6.027	-0.137	-0.628	-0.586	-54.097
Diflunisal	250.198	22494-42-4	-6.019	-0.334	-1.118	-0.2	-23.224
Aminophylline (Truphylline)	420.426	317-34-0	-6.014	-0.463	-0.645	-0.32	-24.036
Swertiamarin	374.34	17388-39-5	-6.013	-0.231	-0.772	-0.312	-25.677
Dinitolmide (Zoalene)	225.158	148-01-6	-6.011	-0.376	-0.657	-0.16	-25.508
Eprosartan Mesylate	520.618	144143-96-4	-6.007	-0.2	-1.432	-0.223	-38.946
Diosmetin (Luteolin 4-methyl ether)	300.263	520-34-3	-6.005	-0.273	-1.299	-0.158	-31.265
Gastrodin (Gastrodine)	286.278	62499-27-8	-6.004	-0.3	-1.236	-0.32	-21.318
Azacitidine (Vidaza)	244.205	320-67-2	-6.001	-0.353	-0.819	-0.16	-23.862
Synephrine HCl	203.666	5985-28-4	-5.998	-0.5	-0.746	-0.96	-11.281
Cefradine	349.405	38821-53-3	-5.996	-0.25	-1.448	-0.63	-40.235
Uridine	244.201	58-96-8	-5.99	-0.352	-0.528	-0.366	-23.32
Olmesartan medoxomil (Benicar)	558.585	144689-63-4	-5.987	-0.146	-1.492	-0.722	-44.57
Sorafenib	464.825	284461-73-0	-5.977	-0.187	-1.443	-0.621	-43.229
Indole-3-carboxylic acid	161.157	771-50-6	-5.966	-0.497	-0.846	-0.281	-20.763
4-Methylumbelliferone (4-MU)	176.169	90-33-5	-5.956	-0.458	-0.449	-0.456	-15.393
Lapatinib	581.058	231277-92-2	-5.954	-0.149	-2.115	-0.14	-49.929
Azathioprine (Azasan, Imuran)	277.263	446-86-6	-5.949	-0.313	-0.543	-0.411	-30.528
Menadione	172.18	58-27-5	-5.94	-0.457	-1.263	-0.32	-24.178
Glutathione	307.323	70-18-8	-5.937	-0.297	-0.333	-0.99	-24.5
Brinzolamide	383.507	138890-62-7	-5.932	-0.258	-0.799	-0.497	-34.742
Silodosin	495.534	160970-54-7	-5.932	-0.169	-1.277	-0.701	-38.592
Sorafenib Tosylate	637.027	475207-59-1	-5.931	-0.185	-1.488	-0.567	-43.639
Cabozantinib (XL184, BMS-907351)	501.506	849217-68-1	-5.919	-0.16	-2.285	-0.552	-47.334
Nalidixic acid (NegGram)	232.235	389-08-2	-5.918	-0.348	-0.708	-0.273	-24.375
Rosmarinic acid	360.315	20283-92-5	-5.915	-0.227	-1.189	0	-31.902
Geneticin (G418 Sulfate)	692.709	108321-42-2	-5.914	-0.174	-0.621	-0.573	-29.156

Hematoxylin (Hydroxybrazilin)	302.279	517-28-2	-5.909	-0.269	-1.199	0	-30.328
Sulfanilamide	172.205	63-74-1	-5.909	-0.537	-0.545	-0.423	-19.774
Levetiracetam	170.209	102767-28-2	-5.902	-0.492	-0.813	-0.16	-20.651
Saxagliptin hydrate	333.425	945667-22-1	-5.896	-0.256	-1.323	-0.657	-29.913
PD 0332991 (Palbociclib) HCl	483.994	827022-32-2, 571	-5.895	-0.179	-1.1	-0.682	-41.33
Pyridoxal 5-phosphate monohydrate	265.157	41468-25-1	-5.889	-0.368	-0.498	-0.297	-20.999
Lapatinib (GW-572016) Ditosylate	925.461	388082-77-7	-5.887	-0.147	-1.695	-0.16	-47.493
Silibinin (Silybin)	482.436	22888-70-6	-5.884	-0.168	-1.507	-0.488	-30.956
7-Aminocephalosporanic acid	272.278	957-68-6	-5.884	-0.327	-0.732	-0.503	-29.531
Daidzein	254.238	486-66-8	-5.881	-0.31	-1.458	-0.399	-27.276
Avibactam sodium	287.226	1192491-61-4	-5.877	-0.346	-0.849	-0.465	-23.5
Adrenalone HCl	217.649	62-13-5	-5.876	-0.452	-0.689	-0.556	-19.485
Felbamate	238.24	25451-15-4	-5.874	-0.346	-1.308	-0.475	-26.447
Amygdalin	457.428	29883-15-6	-5.87	-0.183	-0.996	0	-28.742
Suprofen (Profenal)	260.308	40828-46-4	-5.866	-0.326	-1.792	-0.239	-27.3
Fenoldopam (mesylate)	401.862	67227-57-0	-5.863	-0.279	-1.116	-0.3	-28.527
Proxyphylline	238.243	603-00-9	-5.862	-0.345	-0.519	-0.403	-27.977
Bemegride	155.194	64-65-3	-5.855	-0.532	-0.699	-0.545	-19.633
Gemcitabine (Gemzar)	263.198	95058-81-4	-5.852	-0.325	-0.312	-0.516	-21.973
Isosorbide	146.141	652-67-5	-5.852	-0.585	-0.93	-0.288	-16.223
Flubendazole (Flutelmium)	313.283	31430-15-6	-5.846	-0.254	-1.15	-0.396	-33.548
ODM-201	398.846	1297538-32-9	-5.845	-0.209	-1.61	-0.432	-37.512
Flunixin meglumin	491.458	42461-84-7	-5.841	-0.278	-1.29	-0.227	-30.371
Indirubin	262.263	479-41-4	-5.839	-0.292	-1.26	-0.533	-28.391
Allopurinol Sodium (Aloprim)	159.101	17795-21-0	-5.839	-0.584	-0.422	-0.456	-17.505
Pomalidomide	273.244	19171-19-8	-5.835	-0.292	-0.672	-0.514	-32.458
Daphnetin	178.142	486-35-1	-5.834	-0.449	-1.208	0	-22.934
Resiquimod	314.382	144875-48-9	-5.832	-0.254	-1.663	-0.435	-34.401
Oxcarbazepine	252.268	28721-07-5	-5.831	-0.307	-1.182	-0.16	-28.851
Oxaceprol	173.167	33996-33-7	-5.83	-0.486	-0.456	-0.546	-19.056
Canagliflozin	444.516	842133-18-0	-5.826	-0.188	-0.946	0	-32.884
Natamycin	665.725	7681-93-8	-5.826	-0.124	-0.294	-0.472	-42.84
Capecitabine (Xeloda)	359.35	154361-50-9	-5.824	-0.233	-1.298	0	-33.757
phthalylsulfacetamide	362.357	131-69-1	-5.823	-0.233	-1.016	-0.309	-34.18
Flumazenil	303.288	78755-81-4	-5.822	-0.265	-1.222	-0.32	-32.015

Lactobionic acid	358.296	96-82-2	-5.821	-0.243	-1.222	0	-20.751
Niflumic acid	282.218	4394-00-7	-5.818	-0.291	-1.311	-0.214	-28.669
AZD9291	499.607	1421373-65-0	-5.818	-0.157	-1.594	-0.463	-49.19
(S)-crizotinib	450.337	1374356-45-2	-5.811	-0.194	-1.584	-0.548	-40.88
Haloperidol (Haldol)	375.864	52-86-8	-5.809	-0.223	-1.64	-0.375	-31.259
Biapenem	350.393	120410-24-4	-5.797	-0.242	-1.01	-0.165	-35.164
Protocatechuic acid	154.12	99-50-3	-5.791	-0.526	-0.593	-0.311	-16.006
Creatinine	113.118	60-27-5	-5.789	-0.724	-0.448	-0.419	-14.146
Gimeracil	145.544	103766-25-2	-5.788	-0.643	-0.767	-0.577	-16.11
Stavudine	224.213	3056-17-5	-5.783	-0.361	-0.572	-0.292	-22.856
Berberine chloride	371.814	633-65-8	-5.782	-0.231	-1.718	0	-30.363
Tobramycin	467.514	32986-56-4	-5.779	-0.181	-0.618	-0.582	-28.471
Ginsenoside Rg1	801.013	22427-39-0	-5.779	-0.103	-0.727	0	-46.209
LDE225 (NVP-LDE225, Erismodegib)	485.498	956697-53-3	-5.778	-0.165	-1.625	-0.396	-43.493
Methylene Blue	319.852	61-73-4	-5.777	-0.289	-1.735	0	-28.687
Adenine hydrochloride	171.588	2922-28-3	-5.772	-0.577	-0.201	-0.497	-16.32
Cephalexin (Cefalexin)	347.389	15686-71-2	-5.769	-0.24	-1.432	-0.321	-39.818
Argatroban	508.634	74863-84-6	-5.769	-0.165	-1.389	-0.96	-37.567
Menbutone	258.269	3562-99-0	-5.767	-0.304	-1.618	-0.304	-27.753
Salidroside (Rhodioloside)	300.304	10338-51-9	-5.766	-0.275	-1.24	-0.324	-22.639
Trigonelline Hydrochloride	173.597	6138-41-6	-5.765	-0.576	-0.526	-0.306	-14.037
Nordihydroguaiaretic acid	302.365	500-38-9	-5.764	-0.262	-1.154	0	-22.909
Ribociclib (LEE011)	434.537	1211441-98-3	-5.763	-0.18	-1.399	-0.472	-42.334
Lifitegrast	615.481	1025967-78-5	-5.763	-0.141	-1.293	-0.304	-50.783
Estradiol	272.382	50-28-2	-5.757	-0.288	-1.293	-0.497	-29.29
Chlorothiazide	295.723	58-94-6	-5.755	-0.339	-0.963	-0.435	-24.324
Nelarabine (Arranon)	297.267	121032-29-9	-5.749	-0.274	-0.603	-0.141	-25.311
Diminazene Aceturate	515.522	908-54-3	-5.747	-0.274	-0.658	-0.484	-29.032
(+,-)-Octopamine HCl	189.639	770-05-8	-5.744	-0.522	-1.043	-0.725	-17.468
VX-809 (Lumacaftor)	452.407	936727-05-8	-5.744	-0.174	-1.128	-0.501	-37.083
Eprodisate (disodium)	248.186	36589-58-9	-5.739	-0.522	0	-0.881	-7.659
Bazedoxifene Acetate	530.655	198481-33-3	-5.738	-0.164	-1.388	-0.632	-43.627
Famotidine (Pepcid)	337.445	76824-35-6	-5.734	-0.287	-0.461	-0.757	-33.811
benzthiazide	431.937	91-33-8	-5.731	-0.22	-1.674	-0.16	-42.301
Rufinamide (Banzel)	238.194	106308-44-5	-5.73	-0.337	-0.462	-0.793	-25.148

Mafenide Acetate	246.283	13009-99-9	-5.721	-0.477	-0.578	-0.41	-19.662
Clindamycin phosphate	504.963	24729-96-2	-5.72	-0.185	-0.945	0	-35.153
Tigecycline	585.649	220620-09-7	-5.717	-0.136	-1.141	-0.194	-48.78
Prednisolone acetate (Omnipred)	402.481	52-21-1	-5.716	-0.197	-0.979	-0.456	-38.954
Teriflunomide	270.207	108605-62-5	-5.715	-0.301	-0.805	-0.479	-20.267
Vanillin	152.147	121-33-5	-5.712	-0.519	-1.197	-0.292	-20.744
Mitoxantrone 2HCl	517.403	70476-82-3	-5.711	-0.178	-0.974	-0.751	-38.232
Mupirocin	500.622	12650-69-0	-5.71	-0.163	-1.684	-0.277	-39.8
Hypoxanthine	136.111	68-94-0	-5.709	-0.571	-0.276	-0.454	-16.683
Ademetionine disulfate tosylate	766.796	97540-22-2	-5.708	-0.211	-0.501	-0.671	-26.593
Furosemide (Lasix)	330.744	54-31-9	-5.708	-0.272	-1.008	-0.515	-34.063
Gallic acid	170.12	149-91-7	-5.707	-0.476	-0.808	0	-20.388
Quercitrin	448.377	522-12-3	-5.706	-0.178	-1.04	0	-34.849
Cladribine	285.687	4291-63-8	-5.704	-0.3	-0.156	-0.329	-27.27
Chlorogenic acid	354.309	327-97-9	-5.7	-0.228	-0.373	-0.32	-32.559
Ezetimibe (Zetia)	409.425	163222-33-1	-5.7	-0.19	-1.837	-0.33	-40.171
Mesterolone	304.467	1424-00-6	-5.698	-0.259	-1.405	-0.291	-32.324
Paclitaxel (Taxol)	853.906	33069-62-4	-5.696	-0.092	-1.588	-0.332	-58.451
Moxifloxacin hydrochloride	437.892	186826-86-8	-5.693	-0.196	-1.594	-0.608	-34.997
Cefaclor	385.823	70356-03-5	-5.693	-0.237	-1.421	-0.324	-39.969
Tranexamic acid (Transamin)	157.21	1197-18-8	-5.689	-0.517	-0.425	-0.568	-12.969
Cinepazide maleate	533.571	26328-04-1	-5.686	-0.19	-1.78	-0.599	-44.524
Docetaxel (Taxotere)	807.879	114977-28-5	-5.685	-0.098	-0.907	-0.154	-57.857
Chromocarb	190.152	4940-39-0	-5.684	-0.406	-0.367	-0.392	-23.094
Besifloxacin HCl (Besivance)	430.301	405165-61-9	-5.683	-0.21	-1.234	-0.501	-41.214
Proflavine Hemisulfate	516.572	1811-28-5	-5.679	-0.355	-1.769	-0.16	-25.288
Zaltoprofen	298.356	74711-43-6	-5.678	-0.27	-1.015	-0.366	-27.86
Naringin (Naringoside)	580.535	10236-47-2	-5.677	-0.138	-1.016	-0.32	-34.321
Vilanterol Trifenate	774.768	503070-58-4	-5.675	-0.177	-2.464	-0.152	-43.523
Estriol	288.381	50-27-1	-5.674	-0.27	-1.031	-0.32	-27.49
Evista (Raloxifene Hydrochloride)	510.044	82640-04-8	-5.674	-0.167	-1.695	-0.328	-46.877
Ethidium bromide	394.308	1239-45-8	-5.669	-0.236	-1.467	-0.16	-30.854
Oxytetracycline (Terramycin)	460.434	79-57-2	-5.668	-0.172	-0.995	-0.194	-37.872
Saccharin	183.185	29769	-5.664	-0.472	-0.837	-0.321	-19.47
Amiloride HCl	266.088	2016-88-8	-5.664	-0.378	-0.994	-0.578	-27.217

Iopromide	791.112	73334-07-3	-5.663	-0.177	-0.395	-0.563	-35.126
Cytisine	190.242	485-35-8	-5.662	-0.404	-1.145	-0.376	-23.721
Pazopanib HCl	473.979	635702-64-6	-5.66	-0.183	-1.385	-0.54	-43.7
4-Aminoantipyrine	203.24	30505	-5.657	-0.377	-1.11	-0.433	-25.516
Ethosuximide	141.168	77-67-8	-5.657	-0.566	-0.783	-0.482	-17.733
Umbelliferone	162.142	93-35-6	-5.656	-0.471	-0.19	-0.456	-13.101
Inulin	342.296	9005-80-5	-5.656	-0.246	-0.71	0	-24.701
Dolutegravir (GSK1349572)	419.379	1051375-16-6	-5.656	-0.189	-1.458	-0.16	-40.38
Methyldopa (Aldomet)	211.215	555-30-6	-5.655	-0.377	-0.84	-0.209	-19.646
Tipiracil hydrochloride	279.123	183204-72-0	-5.653	-0.353	-0.891	-0.432	-26.847
piromidic acid	288.302	19562-30-2	-5.649	-0.269	-1.013	-0.499	-32.442
Pyrogallol	126.11	87-66-1	-5.647	-0.627	-0.885	0	-14.992
Pyridoxine	169.178	65-23-6	-5.646	-0.471	-0.898	-0.16	-13.782
Dyphylline (Dilor)	254.243	479-18-5	-5.646	-0.314	-0.469	-0.334	-29.008
Trelagliptin	357.382	865759-25-7	-5.645	-0.217	-1.284	-0.648	-31.337
Silymarin (Silybin B)	482.436	65666-07-1	-5.642	-0.161	-1.101	-0.583	-42.269
Flufenamic acid	281.23	530-78-9	-5.641	-0.282	-1.248	-0.143	-25.507
Lawsone	174.153	83-72-7	-5.639	-0.434	-0.785	-0.21	-24.157
Sitagliptin phosphate monohydrate	523.324	654671-77-9	-5.639	-0.201	-0.469	-0.424	-32.817
Lonidamine	321.158	50264-69-2	-5.637	-0.268	-1.276	-0.219	-31.883
Epinephrine bitartrate (Adrenalinium)	333.291	51-42-3	-5.636	-0.434	-0.955	-0.48	-15.147
Isepamicin Sulphate	667.682	67814-76-0	-5.635	-0.144	-0.515	-0.392	-31.567
Pazufloxacin mesylate	414.405	163680-77-1	-5.635	-0.245	-1.034	-0.288	-30.473
Nadifloxacin	360.379	124858-35-1	-5.634	-0.217	-0.697	-0.292	-33.893
Acetazolamide	222.245	59-66-5	-5.634	-0.433	-0.417	-0.576	-26.189
Harmine hydrochloride	248.708	343-27-1	-5.632	-0.352	-1.265	-0.346	-22.868
PTC124 (Ataluren)	284.242	775304-57-9	-5.63	-0.268	-1.13	-0.287	-28.557
Tolcapone	273.241	134308-13-7	-5.63	-0.282	-0.95	0	-24.937
Baicalein	270.237	491-67-8	-5.629	-0.281	-1.315	0	-29.488
Carbamazepine (Carbatrol)	236.269	298-46-4	-5.622	-0.312	-1.327	-0.228	-27.44
Norethindrone (Norethisterone)	298.419	68-22-4	-5.621	-0.256	-1.481	-0.252	-26.715
Ethamsylate	263.311	2624-44-4	-5.621	-0.468	-0.267	-0.242	-15.64
Ceftriaxone Sodium Trihydrate	598.544	104376-79-6	-5.62	-0.156	-0.63	-0.36	-48.457
Idoxuridin	354.099	54-42-2	-5.615	-0.33	-0.636	-0.505	-22.841
Docetaxel Trihydrate	861.925	148408-66-6	-5.615	-0.097	-1.254	-0.063	-56.379

Amoxicillin sodium (Amox)	387.386	34642-77-8	-5.614	-0.225	-1.442	-0.387	-35.117
Tolmetin	257.284	26171-23-3	-5.614	-0.295	-1.279	-0.254	-28.305
Triapine	195.245	236392-56-6	-5.612	-0.432	-1.011	-0.371	-22.763
Aminoglutethimide (Cytadren)	232.278	125-84-8	-5.612	-0.33	-0.514	-0.656	-23.303
Sotalol HCl	308.825	959-24-0	-5.61	-0.312	-1.46	-0.43	-27.408
Indapamide (Lozol)	365.835	26807-65-8	-5.608	-0.234	-1.221	-0.238	-36.256
Prazosin HCl	419.862	19237-84-4	-5.608	-0.2	-1.232	-0.323	-38.861
Pyridoxine hydrochloride	205.639	58-56-0	-5.606	-0.467	-0.895	-0.16	-13.258
L (+)-Rhamnose Monohydrate	182.172	10030-85-0	-5.603	-0.509	-0.651	0	-17.227
L-Rhamnose monohydrate	182.172	6155-35-7	-5.601	-0.509	-0.807	0	-19.502
Flucytosine (Ancobon)	129.092	2022-85-7	-5.6	-0.622	-0.449	-0.593	-14.772
Salicylic acid	138.121	69-72-7	-5.597	-0.56	-0.901	-0.201	-14.6
Flumequine	261.248	42835-25-6	-5.595	-0.294	-0.714	-0.204	-24.981
Abitrexate (Methotrexate)	454.439	21672	-5.595	-0.17	-0.488	-0.419	-52.426
Halobetasol Propionate	484.96	66852-54-8	-5.592	-0.169	-2.001	-0.318	-36.464
Albendazole Oxide (Ricobendazole)	281.331	54029-12-8	-5.591	-0.294	-1.393	-0.094	-28.447
Posaconazole	700.777	171228-49-2	-5.591	-0.11	-1.389	-0.2	-61.631
Cytosine	111.102	71-30-7	-5.591	-0.699	-0.452	-0.593	-14.254
Clobetasol propionate	466.97	25122-46-7	-5.59	-0.175	-1.997	-0.354	-34.704
Methscopolamine (Pamine)	398.291	155-41-9	-5.59	-0.243	-1.632	0	-30.068
Triamterene	253.263	396-01-0	-5.584	-0.294	-1.365	-0.316	-29.839
Roxadustat (FG-4592)	352.341	808118-40-3	-5.584	-0.215	-1.235	-0.155	-34.205
Fludrocortisone acetate	422.487	514-36-3	-5.584	-0.186	-0.948	-0.45	-37.888
Pranoprofen	255.269	52549-17-4	-5.58	-0.294	-0.856	-0.249	-23.863
ractopamine hydrochloride	337.841	90274-24-1	-5.579	-0.254	-0.776	-1.057	-22.645
CB-5083	413.472	1542705-92-9	-5.579	-0.18	-1.597	-0.24	-42.724
isoetharine mesylate	335.416	7279-75-6	-5.579	-0.328	-1.077	-0.265	-21.365
Tamibarotene	351.439	94497-51-5	-5.577	-0.215	-1.196	-0.152	-35.058
MK-4827(Niraparib)	320.388	1038915-60-4	-5.576	-0.232	-1.411	-0.425	-34.785
Floxuridine	246.192	50-91-9	-5.575	-0.328	-0.578	-0.432	-21.65
Nebivolol HCl	441.896	152520-56-4	-5.575	-0.192	-1.453	0	-33.276
meticrane	275.345	1084-65-7	-5.574	-0.328	-1.002	-0.16	-26.284
Enoxacin (Penetrex)	320.319	74011-58-8	-5.573	-0.242	-0.896	-0.152	-28.171
Dinoprostone	352.465	363-24-6	-5.573	-0.223	-1.236	-0.66	-23.886
Trimebutine	387.469	39133-31-8	-5.573	-0.199	-1.995	-0.486	-38.079

Olaparib (AZD2281, Ku-0059436)	434.463	763113-22-0	-5.572	-0.174	-1.488	-0.654	-42.097
Harmine	212.247	442-51-3	-5.569	-0.348	-1.229	-0.315	-23.907
Scopoletin	192.168	92-61-5	-5.564	-0.397	-1.706	0	-24.189
Imiquimod	240.304	99011-02-6	-5.563	-0.309	-1.488	-0.259	-29.935
Sulindac (Clinoril)	356.411	38194-50-2	-5.561	-0.222	-1.605	-0.32	-35.144
Candesartan cilexetil (Atacand)	610.66	145040-37-5	-5.558	-0.124	-2.117	-0.16	-49.841
Bumetanide	364.416	28395-03-1	-5.556	-0.222	-0.943	-0.613	-33.244
Pazopanib	437.518	444731-52-6	-5.555	-0.179	-1.533	-0.553	-41.841
Valsartan (Diovan)	435.519	137862-53-4	-5.55	-0.173	-1.139	-0.152	-42.152
Atipamezole hydrochloride	248.751	104075-48-1	-5.549	-0.347	-1.459	-0.365	-22.022
Menadiol Diacetate	258.269	573-20-6	-5.547	-0.292	-1.25	-0.515	-29.732
Resorcinol	110.111	108-46-3	-5.546	-0.693	-0.902	-0.456	-14.375
Mafenide hydrochloride	222.692	138-37-4	-5.546	-0.462	-0.51	-0.304	-22.444
Palmatine	352.404	3486-67-7	-5.545	-0.213	-1.329	-0.209	-32.333
Itraconazole (Sporanox)	705.633	84625-61-6	-5.544	-0.113	-1.416	0	-60.924
Lumiracoxib (COX-189)	293.721	220991-20-8	-5.544	-0.277	-1.57	-0.102	-30.343
Chlortetracycline HCl	515.341	64-72-2	-5.543	-0.168	-1.493	-0.249	-38.573
L-Adrenaline (Epinephrine)	183.204	51-43-4	-5.54	-0.426	-0.954	-0.426	-16.583
Ceftizoxime	383.403	68401-81-0	-5.54	-0.222	-0.944	-0.527	-42.894
Oxaliplatin (Eloxatin)	397.286	61825-94-3	-5.54	-0.692	-0.721	-0.719	-14.553
Sulfathiazole	255.317	72-14-0	-5.539	-0.346	-0.918	-0.429	-27.298
Fenofibric acid	318.752	42017-89-0	-5.538	-0.252	-1.967	-0.216	-31.999
Pidotimod	244.268	121808-62-6	-5.536	-0.346	-0.728	-0.248	-28.608
Adenine sulfate	368.332	321-30-2	-5.536	-0.554	-0.122	-0.346	-19.88
Tenofovir (Viread)	287.212	147127-20-6	-5.528	-0.291	-0.549	-0.32	-26.72
Ciclopirox (Penlac)	207.269	29342-05-0	-5.527	-0.368	-1.532	-0.301	-26.131
Tedizolid Phosphate	450.318	856867-55-5	-5.525	-0.178	-1.076	0	-40.219
tacrine hydrochlorid	234.725	1684-40-8	-5.518	-0.368	-1.72	-0.117	-25.553
Avanafil	483.951	330784-47-9	-5.516	-0.162	-1.161	-0.833	-40.955
Prasugrel Hydrochloride	409.902	389574-19-0	-5.516	-0.212	-1.636	-0.228	-36.684
Veratric acid	182.173	34152	-5.515	-0.424	-1.315	-0.304	-22.279
Alosetron Hydrochloride	330.812	122852-69-1	-5.512	-0.251	-1.029	-0.356	-33.137
Tacrine hydrochloride hydrate	198.264	206658-92-6	-5.51	-0.367	-1.716	-0.115	-25.526
Butylscopolamine bromide (Scopolamine butylb	440.371	149-64-4	-5.509	-0.212	-1.807	0	-33.468
Fumalic acid (Ferulic acid)	194.184	1135-24-6	-5.508	-0.393	-0.324	-0.25	-18.781

spironolactone	416.573	19000	-5.508	-0.19	-1.263	-0.506	-37.398
Ripasudil (K-115)	395.877	887375-67-9	-5.505	-0.25	-1.164	-0.315	-31.842
Cyclo (-RGDfK)	717.694	161552-03-0	-5.503	-0.128	-0.745	-0.778	-46.452
Fosaprepitant dimeglumine salt	1004.834	265121-04-8	-5.503	-0.134	-0.574	-0.255	-46.391
Succinylsulfathiazole	355.389	116-43-8	-5.502	-0.239	-0.691	-0.185	-33.466
Indobufen	295.332	63610-08-2	-5.502	-0.25	-1.582	-0.157	-33.174
Alizapride hydrochloride	351.831	59338-87-3	-5.5	-0.239	-0.982	-0.498	-29.493
Esculetin	178.142	305-01-1	-5.498	-0.423	-0.329	-0.288	-15.939
Nitroxoline	190.156	4008-48-4	-5.497	-0.393	-0.837	-0.16	-23.754
Norfloxacin (Norxacin)	319.331	70458-96-7	-5.495	-0.239	-0.967	-0.152	-29.833
Prasugrel (Effient)	373.441	150322-43-3	-5.494	-0.211	-1.62	-0.226	-36.747
Ganciclovir	255.231	82410-32-0	-5.49	-0.305	-0.414	-0.432	-24.519
Amodiaquin (dihydrochloride dihydrate)	464.814	6398-98-7	-5.489	-0.22	-1.763	-0.436	-35.275
Ribavirin (Copegus)	244.205	36791-04-5	-5.486	-0.323	-0.07	-0.343	-23.332
Sodium Monofluorophosphate	143.95						
Amonafide	283.325	69408-81-7	-5.485	-0.261	-1.002	-0.317	-31.607
Metolazone (Zaroxolyn)	365.835	17560-51-9	-5.485	-0.229	-1.222	-0.272	-35.164
MK-4827(Niraparib) tosylate	492.59	1038915-73-9	-5.485	-0.229	-1.392	-0.361	-34.836
Ampicillin Trihydrate	403.451	7177-48-2	-5.482	-0.228	-0.871	-0.429	-36.524
Oxiracetam	158.155	62613-82-5	-5.479	-0.498	-0.689	-0.557	-21.128
Dicoumarol	336.295	66-76-2	-5.479	-0.219	-1.924	-0.308	-38.466
Methyclothiazide	360.237	135-07-9	-5.477	-0.274	-0.543	-0.16	-34.213
Ibandronate sodium	360.234	138926-19-9	-5.476	-0.288	-0.908	0	-20.652
E7080 (Lenvatinib)	426.853	417716-92-8	-5.47	-0.182	-1.643	-0.379	-43.559
Sulfisoxazole	267.304	127-69-5	-5.47	-0.304	-1.056	-0.308	-23.936
Hexachlorophene	406.904	70-30-4	-5.468	-0.26	-1.872	-0.304	-40.647
Diazoxide	230.671	364-98-7	-5.466	-0.39	-1.453	-0.004	-28.802
Naftopidil (Flivas)	392.491	57149-07-2	-5.465	-0.188	-1.216	-0.715	-31.909
Sodium salicylate	161.111	54-21-7	-5.464	-0.546	-1.081	-0.09	-15.727
LX1606 hippurate	754.155	11137608-69-5	-5.463	-0.137	-1.399	-0.546	-47.621
Terazosin HCl	423.894	63074-08-8	-5.462	-0.195	-1.648	-0.42	-39.935
Pentamidine isethionate	592.683	140-64-7	-5.462	-0.218	-1.062	-0.382	-32.289
Balofloxacin	389.421	127294-70-6	-5.46	-0.195	-0.556	-0.307	-31.625
Atipamezole	212.29	104054-27-5	-5.458	-0.341	-1.39	-0.363	-21.932
Cefditoren pivoxil	620.721	117467-28-4	-5.456	-0.133	-1.029	-0.195	-59.081

L-5-Hydroxytryptophan	220.225	895096	-5.455	-0.341	-1.145	-0.327	-26.072
Salbutamol sulfate (Albuterol)	337.389	51022-70-9	-5.452	-0.321	-1.225	-0.559	-19.669
Potassium Canrenoate	396.562	102731	-5.452	-0.21	-1.774	-0.413	-26.082
Deferiprone	139.152	30652-11-0	-5.452	-0.545	-1.057	-0.214	-17.92
pasiniazid	290.275	2066-89-9	-5.451	-0.496	-0.787	-0.288	-16.366
Sophoridine	248.364	6882-68-4	-5.45	-0.303	-1.147	-0.252	-29.146
Naftopidil Dihydrochloride	465.413	57149-08-3	-5.45	-0.188	-1.274	-0.719	-32.443
Ramosetron Hydrochloride	315.797	132907-72-3	-5.45	-0.26	-1.233	-0.286	-28.167
Propylthiouracil	170.232	51-52-5	-5.446	-0.495	-0.618	-0.291	-23.326
Cabozantinib malate	635.593	1140909-48-3	-5.445	-0.147	-1.855	-0.506	-45.186
Caffeic acid	180.157	331-39-5	-5.44	-0.418	-0.275	-0.143	-14.209
Fasudil HCl (HA-1077)	327.83	105628-07-7	-5.439	-0.272	-1.184	-0.241	-30.871
Sisomicin sulfate	1385.445	53179-09-2	-5.437	-0.175	-1.075	-0.948	-28.542
D-Galactose	180.156	59-23-4	-5.435	-0.453	-0.263	-0.16	-14.339
cinoxacin	262.218	28657-80-9	-5.434	-0.286	-0.73	-0.702	-29.118
Paeonol (Peonol)	166.174	552-41-0	-5.434	-0.453	-0.909	-0.175	-19.711
Lactulose	342.296	4618-18-2	-5.431	-0.236	-0.438	0	-21.712
R788 (Fostamatinib)	580.46	901119-35-5	-5.428	-0.136	-0.872	-0.011	-50.551
Erythromycin Ethylsuccinate	862.053	1264-62-6	-5.428	-0.09	-1.295	-0.119	-50.041
ethoxzolamide	258.317	452-35-7	-5.427	-0.339	-0.801	-0.28	-24.965
metaproterenol sulfate	520.594	5874-97-5	-5.426	-0.362	-0.809	-0.8	-19.92
Zalcitabine	211.218	7481-89-2	-5.423	-0.362	-0.218	-0.546	-23.977
Mesalamine (Lialda)	153.135	89-57-6	-5.423	-0.493	-0.569	-0.475	-18.036
Tolfenamic acid	261.704	13710-19-5	-5.421	-0.301	-1.266	-0.187	-28.958
Tranilast (SB 252218)	327.331	53902-12-8					
Cabazitaxel (Jevtana)	835.932	183133-96-2	-5.42	-0.09	-1.35	0	-60.138
Triflusal	248.155	322-79-2	-5.415	-0.319	-1	-0.092	-22.198
Chloroxine	214.048	773-76-2	-5.415	-0.417	-1.272	-0.32	-23.554
Methandrostenolone	300.435	72-63-9	-5.414	-0.246	-1.062	-0.296	-31.067
Zanamivir (Relenza)	332.31	139110-80-8	-5.413	-0.235	-0.376	-0.402	-27.466
4-Hydroxybenzoic acid	138.121	99-96-7	-5.412	-0.541	-0.741	-0.392	-14.476
Methylthiouracil	142.179	20547	-5.412	-0.601	-0.745	-0.16	-22.391
Isopsoralen	186.164	523-50-2	-5.41	-0.386	-1.603	-0.171	-24.997
Tanshinone I	276.286	568-73-0	-5.41	-0.258	-1.463	-0.01	-31.067
Regorafenib (BAY 73-4506)	482.815	755037-03-7	-5.409	-0.164	-1.316	-0.304	-41.08

Dehydrocholic acid	402.524	81-23-2	-5.409	-0.187	-1.28	-0.531	-29.326
Paliperidone	426.484	144598-75-4	-5.407	-0.174	-1.097	-0.407	-40.139
Doxofylline	266.253	69975-86-6	-5.406	-0.285	-0.715	-0.267	-30.667
Pranlukast	481.503	103177-37-3	-5.405	-0.15	-2.133	-0.299	-53.218
Isoniazid (Tubizid)	137.139	54-85-3	-5.405	-0.54	-0.65	-0.529	-19.619
Bergapten	216.19	484-20-8	-5.399	-0.337	-1.607	-0.233	-27.96
Allopurinol (Zyloprim)	136.111	315-30-0	-5.399	-0.54	-0.523	-0.16	-20.901
Benzenesulfonamide	157.19	36070	-5.397	-0.54	-0.483	-0.456	-15.878
Psoralen	186.164	66-97-7	-5.395	-0.385	-1.304	-0.201	-23.737
Nilutamide	317.221	63612-50-0	-5.394	-0.245	-0.976	-0.32	-34.844
Milrinone (Primacor)	211.219	78415-72-2	-5.39	-0.337	-1.294	-0.272	-26
Fludarabine (Fludara)	285.232	21679-14-1	-5.389	-0.269	-0.513	-0.288	-24.344
Rebamipide	370.786	90098-04-7	-5.389	-0.207	-1.396	-0.352	-37.721
Palmatine chloride	387.857	10605-02-4	-5.387	-0.207	-1.217	-0.32	-31.674
Ronidazole	200.152	7681-76-7	-5.385	-0.385	-0.378	-0.289	-21.493
Danofloxacin Mesylate	453.484	119478-55-6	-5.385	-0.207	-0.824	-0.2	-31.404
Retigabine	303.331	150812-12-7	-5.384	-0.245	-1.912	-0.618	-32.39
Ketorolac	255.269	74103-07-4	-5.383	-0.283	-1.169	0	-30.475
Sulfaguanidine	214.245	57-67-0	-5.38	-0.384	-0.626	-0.768	-21.34
Piroxicam (Feldene)	331.346	36322-90-4	-5.379	-0.234	-0.857	-0.245	-34.955
Niacin (Nicotinic acid)	123.109	59-67-6	-5.378	-0.598	-0.549	-0.274	-16.089
Alpelisib (BYL719)	441.47	1217486-61-7	-5.378	-0.179	-1.03	-0.544	-33.829
Oxfendazole	315.347	53716-50-0	-5.378	-0.244	-1.343	-0.16	-30.06
Sulfachloropyridazine	284.722	80-32-0	-5.377	-0.299	-0.918	-0.31	-24.496
Cefepime Dihydrochloride Monohydrate	571.498	123171-59-5	-5.376	-0.168	-1.096	-0.529	-37.193
anisindione	252.265	117-37-3	-5.374	-0.283	-1.743	-0.027	-28.849
Mefloquine HCl	414.773	51773-92-3	-5.373	-0.207	-1.125	-0.16	-28.621
Benzoic acid	122.121	65-85-0	-5.372	-0.597	-0.563	-0.288	-15.423
anisotropine methylbromide	362.345	80-50-2	-5.371	-0.269	-2.049	-0.32	-22.99
Geniposide	388.366	24512-63-8	-5.371	-0.199	-0.686	-0.311	-33.317
Phenindione (Rectadione)	222.239	30655	-5.37	-0.316	-1.897	0	-27.896
Bithionol	356.052	97-18-7	-5.368	-0.283	-1.643	-0.304	-33.192
Edaravone (MCI-186)	174.199	89-25-8	-5.367	-0.413	-0.886	-0.257	-21.579
L(+)-Arabinose	150.13	87-72-9	-5.364	-0.536	-0.567	0	-17.464
Enalapril maleate (Vasotec)	492.519	76095-16-4	-5.363	-0.199	-1.434	-0.304	-37.322

Teniposide (Vumon)	656.654	29767-20-2	-5.363	-0.117	-1.189	0	-49.789
Marimastat (BB-2516)	331.408	154039-60-8	-5.361	-0.233	-0.565	-0.605	-29.153
Levonorgestrel (Levonelle)	312.446	797-63-7	-5.36	-0.233	-1.326	-0.377	-20.429
Fenbendazole (Panacur)	299.348	43210-67-9	-5.358	-0.255	-1.245	-0.16	-29.299
Carprofen	273.714	53716-49-7	-5.352	-0.282	-0.899	-0.225	-23.586
Kitasamycin	785.958	1392-21-8	-5.352	-0.097	-1.906	0	-49.503
Sodium Danshensu	220.155	67920-52-9	-5.351	-0.382	-1.057	-0.14	-17.309
TriacetonaMine	155.237	826-36-8	-5.351	-0.486	-1.047	-0.226	-21.548
VX-661	520.498	1152311-62-0	-5.35	-0.145	-1.121	-0.478	-39.116
Acotiamide hydrochloride	541.058	773092-05-0	-5.35	-0.173	-1.395	-0.563	-43.397
Amikacin disulfate	781.759	39831-55-5	-5.349	-0.134	-0.486	-0.585	-29.804
Indomethacin (Indocid, Indocin)	357.788	53-86-1	-5.342	-0.214	-1.845	-0.213	-39.047
Nicotinamide (Vitamin B3)	122.125	98-92-0	-5.342	-0.594	-0.629	-0.329	-17.074
Flopropione	182.173	2295-58-1	-5.341	-0.411	-0.853	-0.304	-19.493
Amiloride hydrochloride dihydrate	302.119	17440-83-4	-5.339	-0.356	-0.643	-0.606	-27.255
Retigabine 2HCl	376.253	150812-13-8	-5.338	-0.243	-1.583	-0.536	-35.57
Exemestane	296.403	107868-30-4	-5.336	-0.243	-1.163	-0.32	-31.748
Levofloxacin hydrate	361.368	138199-71-0	-5.33	-0.205	-0.853	-0.225	-29.736
Isatin	147.131	91-56-5	-5.328	-0.484	-0.614	-0.204	-21.826
Epinephrine HCl (Adrenaline)	219.665	55-31-2	-5.328	-0.41	-0.775	-0.288	-17.517
Flavone	222.239	525-82-6	-5.325	-0.313	-1.966	-0.169	-28.889
(R)-baclofen	213.661	69308-37-8	-5.325	-0.38	-0.723	-0.376	-18.503
Sasapyrine	258.226	552-94-3	-5.324	-0.28	-1.042	0	-26.225
Ranolazine (Ranexa)	427.537	95635-55-5	-5.321	-0.172	-1.356	-0.658	-38.008
Terbutaline Sulfate	548.647	23031-32-5	-5.32	-0.333	-1.444	-0.529	-22.607
Faropenem Sodium	307.298	122547-49-3	-5.313	-0.28	-1.083	-0.165	-29.683
Sarafloxacin HCl	421.825	91296-87-6	-5.313	-0.19	-0.946	-0.301	-37.318
Telbivudine (Sebivo, Tyzeka)	242.229	3424-98-4	-5.311	-0.312	-0.577	-0.316	-22.644
Voglibose	267.276	83480-29-9	-5.311	-0.295	-0.265	-0.071	-16.145
Nefiracetam (Translon)	246.305	77191-36-7	-5.311	-0.295	-1.403	-0.32	-27.858
Fulvestrant (Faslodex)	606.771	129453-61-8	-5.31	-0.13	-2.261	-0.186	-42.894
Quinine hydrochloride dihydrate	396.908	6119-47-7	-5.308	-0.221	-1.467	-0.549	-32.64
Tioxolone	168.17	4991-65-5	-5.307	-0.482	-0.91	-0.332	-21.716
Lomefloxacin hydrochloride (Maxaquin)	387.809	98079-52-8	-5.307	-0.212	-0.943	-0.152	-31.868
Altretamine (Hexalen)	210.279	645-05-6	-5.306	-0.354	-0.634	0	-24.443

Diclofenac Potassium	334.239	15307-81-0	-5.305	-0.279	-1.449	-0.32	-25.946
Citiolone	159.206	1195-16-0	-5.303	-0.53	-0.567	-0.282	-21.94
CAL-101 (GS-1101)	415.423	870281-82-6	-5.303	-0.171	-1.563	-0.36	-42.044
Omeprazole (Prilosec)	345.416	73590-58-6	-5.3	-0.221	-1.017	-0.16	-29.783
Acesulfame Potassium	202.25	55589-62-3	-5.296	-0.53	-0.371	-0.32	-20.745
Forsythin	534.552	487-41-2	-5.295	-0.139	-1.362	-0.127	-34.215
Lamivudine (Epivir)	229.256	134678-17-4	-5.295	-0.353	-0.325	-0.456	-23.882
Nevirapine (Viramune)	266.298	129618-40-2	-5.295	-0.265	-1.434	-0.32	-27.015
D-Cycloserine	102.092	68-41-7	-5.294	-0.756	-0.408	-0.44	-12.326
Puerarin (Kakonein)	432.378	3681-99-0	-5.292	-0.171	-1.122	-0.29	-32.624
Sulfaphenazole	314.362	526-08-9	-5.29	-0.24	-1.272	-0.347	-34.817
D-Phenylalanine	165.189	673-06-3	-5.29	-0.441	-1.168	-0.378	-18.723
Bortezomib (Velcade)	384.237	179324-69-7	-5.286	-0.189	-1.152	-0.31	-34.288
Curcumin	368.38	458-37-7	-5.284	-0.196	-1.42	-0.356	-39.502
Baicalin	446.361	21967-41-9	-5.282	-0.165	-0.409	0	-30.226
metaraminol bitartrate	317.292	33402-03-8	-5.282	-0.44	-0.609	-0.629	-18.799
Piribedil	298.34	622744	-5.277	-0.24	-2.037	-0.2	-35.081
Trospium chloride (Sanctura)	427.964	10405-02-4	-5.275	-0.182	-1.451	-0.204	-33.095
Tyramine	137.179	51-67-2	-5.272	-0.527	-0.757	-0.576	-11.842
Topiroxostat	248.243	577778-58-6	-5.272	-0.277	-0.726	-0.385	-30.873
Taurolidine	284.356	19388-87-5	-5.267	-0.31	-0.339	-0.456	-28.769
Anagrelide hydrochloride	292.549	58579-51-4	-5.267	-0.329	-0.856	-0.412	-27.819
Sulfogaiacol	242.291	1321-14-8	-5.266	-0.405	-1.008	-0.306	-18.833
Risedronate sodium	305.094	115436-72-1	-5.262	-0.31	-0.274	-0.453	-17.173
Abacavir sulfate	670.743	188062-50-2	-5.262	-0.251	-1.035	-0.479	-31.445
Elvitegravir (GS-9137)	447.884	697761-98-1	-5.262	-0.17	-1.333	-0.153	-42.882
Chloramphenicol (Chloromycetin)	323.129	56-75-7	-5.262	-0.263	-0.655	-0.304	-29.558
Aceclidine HCl	205.682	6109-70-2	-5.261	-0.438	-1.21	-0.208	-20.824
Epiandrosterone (خ3┬-androsterone)	290.44	481-29-8	-5.259	-0.25	-1.279	-0.32	-30.654
Daunorubicin HCl (Daunomycin HCl)	563.981	23541-50-6	-5.259	-0.138	-0.853	-0.41	-34.727
Atovaquone (Atavaquone)	366.837	95233-18-4	-5.256	-0.202	-1.338	-0.431	-36.769
Baricitinib (LY3009104)	371.417	1187594-09-7	-5.254	-0.202	-0.82	-0.506	-38.737
Brivudine	333.135	69304-47-8	-5.254	-0.277	-1.289	-0.238	-31.269
Aceclidine Hydrochloride	205.682	56715-13-0	-5.253	-0.438	-1.19	-0.232	-20.556
Loxoprofen	246.302	68767-14-6	-5.253	-0.292	-1.353	-0.124	-20.962

Brimonidine Tartrate	442.221	70359-46-5	-5.25	-0.309	-1.164	-0.298	-27.189
Ampicillin sodium	371.387	69-52-3	-5.247	-0.219	-0.888	-0.329	-31.471
Ibudilast	230.306	50847-11-5	-5.247	-0.309	-1.108	-0.244	-26.18
Irbesartan (Avapro)	428.529	138402-11-6	-5.246	-0.164	-1.406	-0.16	-44.648
Cefmenoxime hydrochloride	1059.578	75738-58-8	-5.246	-0.159	-0.765	-0.532	-50.674
Osalmid	229.231	526-18-1	-5.246	-0.309	-1.212	-0.304	-24.926
Medroxyprogesterone	344.488	520-85-4	-5.245	-0.21	-1.44	0	-36.346
Dipyridamole (Persantine)	504.626	58-32-2	-5.244	-0.146	-0.619	-0.804	-42.514
Acalabrutinib (ACP-196)	465.507	1420477-60-6	-5.244	-0.15	-1.861	-0.224	-48.201
Indacaterol Maleate	508.563	753498-25-8	-5.235	-0.181	-1.064	-0.649	-32.897
Carteolol HCl	328.834	51781-21-6	-5.235	-0.249	-0.903	-0.487	-27.553
Tegafur (FT-207, NSC 148958)	200.167	17902-23-7	-5.235	-0.374	-0.694	-0.16	-24.925
Cefuroxime axetil	510.474	64544-07-6	-5.235	-0.15	-0.875	-0.754	-47.37
Benzbromarone	424.083	3562-84-3	-5.234	-0.238	-1.551	-0.439	-37.083
Melatonin	232.278	73-31-4	-5.233	-0.308	-1.247	-0.427	-30.473
Istradefylline (KW-6002)	384.429	155270-99-8	-5.23	-0.187	-0.523	-0.191	-39.764
Etoricoxib	358.842	202409-33-4	-5.228	-0.218	-1.476	-0.32	-34.791
Dofetilide (Tikosyn)	441.565	115256-11-6	-5.228	-0.18	-1.482	-0.233	-40.51
Doxorubicin (Adriamycin) HCl	579.98	25316-40-9	-5.227	-0.134	-0.545	-0.29	-36.014
Ketoprofen (Actron)	254.281	22071-15-4	-5.226	-0.275	-1.331	-0.161	-30.041
Acipimox	154.123	51037-30-0	-5.222	-0.475	-0.891	-0.106	-16.135
LCZ696	915.979	936623-90-4	-5.222	-0.163	-0.788	-0.266	-43.912
Lornoxicam (Xefo)	371.819	70374-39-9	-5.221	-0.227	-1.476	-0.197	-38.99
Benzyl alcohol	108.138	100-51-6	-5.22	-0.653	-1.097	-0.289	-14.951
Obeticholic Acid	420.625	459789-99-2	-5.218	-0.174	-0.988	-0.495	-28.753
Losartan potassium	462.009	124750-99-8	-5.218	-0.174	-1.19	-0.285	-42.393
2-Methoxyestradiol	302.408	362-07-2	-5.217	-0.237	-1.279	-0.16	-21.632
Acemetacin (Emflex)	415.824	53164-05-9	-5.216	-0.18	-1.414	-0.312	-39.898
Penciclovir	253.258	39809-25-1	-5.214	-0.29	-0.432	-0.229	-28.655
N-Ethylmaleimide (NEM)	125.125	128-53-0	-5.213	-0.579	-0.471	-0.32	-17.151
Progesterone (Prometrium)	314.462	57-83-0	-5.212	-0.227	-1.075	-0.313	-32.243
Thiamphenicol (Thiophenicol)	356.222	15318-45-3	-5.209	-0.248	-0.449	-0.37	-27.072
Chlorquinaldol	228.075	72-80-0	-5.209	-0.372	-1.245	-0.318	-24.057
Formononetin (Formononetol)	268.264	485-72-3	-5.207	-0.26	-1.598	-0.257	-29.704
Amantadine hydrochloride (Symmetrel)	187.71	665-66-7	-5.206	-0.473	-0.897	-0.456	-18.467

Triclabendazole	359.658	68786-66-3	-5.205	-0.248	-1.63	-0.32	-33.458
Harmaline	214.263	304-21-2	-5.204	-0.325	-1.048	-0.127	-24.994
Antipyrine	188.226	60-80-0	-5.202	-0.372	-1.224	-0.32	-24.653
Levofloxacin (Levaquin)	361.368	100986-85-4	-5.202	-0.2	-0.997	-0.306	-31.534
Serotonin HCl	212.676	153-98-0	-5.195	-0.4	-0.856	-0.314	-18.015
Bicalutamide (Casodex)	430.373	90357-06-5	-5.195	-0.179	-0.668	-0.214	-39.328
Moroxydine HCl	207.661	3160-91-6	-5.195	-0.433	-0.616	-0.53	-19.601
Histamine Phosphate	307.135	51-74-1	-5.194	-0.649	-0.319	-0.554	-9.987
Trilostane	329.433	13647-35-3	-5.194	-0.216	-1.464	-0.16	-35.087
Dihydrotestosterone (DHT)	290.44	521-18-6	-5.193	-0.247	-1.474	-0.16	-32.283
Minocycline HCl	493.937	13614-98-7	-5.19	-0.157	-0.78	-0.163	-39.711
Crisaborole (AN2728)	251.045	906673-24-3	-5.188	-0.273	-1	-0.219	-23.443
Deoxycorticosterone acetate	372.498	56-47-3	-5.186	-0.192	-1.132	-0.32	-40.648
Amlexanox	298.293	68302-57-8	-5.184	-0.236	-2.048	0	-32.315
Maltol	126.11	118-71-8	-5.184	-0.576	-1.18	-0.217	-16.558
Sodium ascorbate	201.13	134-03-2	-5.183	-0.432	-0.891	-0.142	-17.185
Povidone iodine	141.211	25655-41-8	-5.182	-0.518	-0.972	-0.255	-16.593
Hexestrol (Bibenzyl)	270.366	84-16-2	-5.181	-0.259	-1.277	-0.454	-25.045
Nafamostat mesylate	539.582	82956-11-4	-5.181	-0.199	-0.91	-0.343	-37.785
Phentolamine Mesylate	377.458	65-28-1	-5.18	-0.247	-1.264	-0.294	-25.463
Rucaparib (AG-014699,PF-01367338) phosphate	421.359	459868-92-9	-5.179	-0.216	-0.565	-0.715	-30.657
Favipiravir (T-705)	157.103	259793-96-9	-5.177	-0.471	-0.528	-0.285	-20.762
p-Coumaric Acid	164.158	501-98-4	-5.176	-0.431	-0.427	-0.402	-15.969
Sulfalene(SMPZ)	280.303	152-47-6	-5.174	-0.272	-0.864	-0.444	-27.817
L-Cycloserine	102.092	339-72-0	-5.171	-0.739	-0.282	-0.613	-14.284
Sparfloxacin	392.4	110871-86-8	-5.171	-0.185	-1.024	-0.388	-38.515
Dopamine hydrochloride (Inotropin)	189.639	62-31-7	-5.17	-0.47	-0.836	-0.144	-17.08
Chloroxylenol	156.609	88-04-0	-5.166	-0.517	-1.166	-0.16	-17.959
Tenoxicam(Mobiflex)	337.374	59804-37-4	-5.165	-0.235	-1.337	-0.289	-36.464
Guaiacol	124.137	32994	-5.164	-0.574	-1.02	-0.151	-17.23
Tioconazole	387.711	65899-73-2	-5.162	-0.224	-1.475	-0.283	-33.099
Kinetin (6-Furfuryladenine)	215.211	525-79-1	-5.16	-0.322	-1.1	-0.472	-28.133
Azaperone	327.396	1649-18-9	-5.16	-0.215	-1.558	-0.538	-30.257
Calcium Dobesilate	189.166	20123-80-2	-5.159	-0.43	-0.093	-0.304	-8.459
2-Thiouracil	128.152	141-90-2	-5.158	-0.645	-0.59	-0.16	-21.266

Acetanilide (Antifebrin)	135.163	103-84-4	-5.158	-0.516	-0.663	-0.371	-19.606
Pirarubicin	627.636	72496-41-4	-5.157	-0.115	-1.097	-0.056	-45.214
Famciclovir (Famvir)	321.332	104227-87-4	-5.157	-0.224	-0.339	-1.015	-30.298
Duvelisib (IPI-145, INK1197)	416.863	1201438-56-3	-5.155	-0.172	-1.52	-0.145	-41.258
Flibanserin	390.402	167933-07-5	-5.155	-0.184	-1.178	-0.315	-34.116
Valaciclovir HCl	360.797	124832-27-5	-5.154	-0.224	-0.941	-0.78	-33.172
Domperidone (Motilium)	425.911	57808-66-9	-5.153	-0.172	-1.367	-0.155	-39.872
Thymidine	242.229	50-89-5	-5.153	-0.303	-0.387	-0.288	-21.938
Decitabine	228.205	2353-33-5	-5.15	-0.322	-0.004	-0.367	-24.311
Enrofloxacin	359.395	93106-60-6	-5.149	-0.198	-1.058	-0.217	-32.313
Mefenamic Acid	241.285	61-68-7	-5.149	-0.286	-0.992	-0.269	-27.265
Ilaprazole	366.437	172152-36-2	-5.147	-0.198	-1.309	-0.16	-36.427
Pimobendan (Vetmedin)	334.372	74150-27-9	-5.146	-0.206	-1.051	-0.16	-32.82
Xanthinol Nicotinate	434.446	437-74-1	-5.145	-0.234	-0.589	-0.544	-32.008
Mirabegron (YM178)	396.506	223673-61-8	-5.145	-0.184	-1.068	-0.864	-34.002
Axitinib	386.47	319460-85-0	-5.144	-0.184	-1.553	-0.175	-42.317
Citric acid trilithium salt tetrahydrate	281.984	6080-58-6	-5.143	-0.321	-0.469	-0.154	-15.229
Fluorometholone Acetate	418.498	694485	-5.143	-0.171	-0.903	-0.453	-39.085
Praziquantel (Biltricide)	312.406	55268-74-1	-5.142	-0.224	-1.113	-0.233	-37.089
L-Tryptophan	204.225	73-22-3	-5.142	-0.343	-0.313	-0.319	-18.623
Masitinib (AB1010)	498.642	790299-79-5	-5.14	-0.143	-2.824	-0.075	-51.476
Naphazoline hydrochloride (Naphcon)	246.735	550-99-2	-5.139	-0.321	-1.208	-0.16	-21.387
Glucosamine hydrochloride	215.632	66-84-2	-5.138	-0.428	-0.145	-0.028	-10.261
Aliskiren Hemifumarate	1219.589	173334-58-2	-5.135	-0.132	-0.838	-0.829	-39.548
(-)-Huperzine A (HupA)	242.316	102518-79-6	-5.135	-0.285	-1.162	-0.276	-26.454
Gramine	174.242	87-52-5	-5.135	-0.395	-0.703	-0.32	-21.062
NEXIUM (esomeprazole magnesium)	713.121	161973-10-0	-5.135	-0.214	-1.059	-0.225	-33.931
Clopidol	192.043	2971-90-6	-5.132	-0.467	-1.422	-0.181	-20.87
Melibiose	342.296	585-99-9	-5.132	-0.223	-0.679	0	-20.561
Corticosterone	346.461	50-22-6	-5.127	-0.205	-0.704	-0.544	-23.736
Furazolidone	225.158	67-45-8	-5.121	-0.32	-0.671	-0.463	-29.384
Miglitol (Glyset)	207.224	72432-03-2	-5.121	-0.366	-0.343	-0.614	-14.417
Uracil	112.087	66-22-8	-5.121	-0.64	-0.368	-0.342	-15.6
Entinostat (MS-275, SNDX-275)	376.409	209783-80-2	-5.121	-0.183	-1.545	-0.374	-41.52
Meloxicam (Mobic)	351.401	71125-38-7	-5.12	-0.223	-0.765	-0.247	-35.744

Dehydroepiandrosterone (DHEA)	288.424	53-43-0	-5.117	-0.244	-1.205	-0.32	-31.933
4-Biphenylacetic acid	212.244	5728-52-9	-5.115	-0.32	-1.589	0	-20.666
Mestranol	310.43	72-33-3	-5.115	-0.222	-1.764	-0.16	-24.765
PCI-32765 (Ibrutinib)	440.497	936563-96-1	-5.115	-0.155	-1.36	-0.268	-46.256
Tiratricol	621.932	51-24-1	-5.114	-0.244	-1.121	-0.304	-30.596
Ethionamide	166.243	536-33-4	-5.113	-0.465	-0.794	-0.213	-23.738
Bazedoxifene HCl	507.063	198480-56-7	-5.112	-0.146	-1.384	-0.51	-44.453
Ciclopirox ethanolamine	268.352	41621-49-2	-5.112	-0.341	-1.13	-0.122	-27.097
Thalidomide	258.229	50-35-1	-5.112	-0.269	-0.51	-0.387	-31.677
GSK1120212 (Trametinib)	615.395	871700-17-3	-5.11	-0.138	-0.936	-0.466	-49.518
Memantine hydrochloride (Namenda)	215.763	41100-52-1	-5.107	-0.393	-1.231	-0.191	-22.73
Amoxicillin (Amoxycillin)	365.404	26787-78-0	-5.105	-0.204	-0.638	-0.152	-36.855
Medetomidine HCl	236.74	86347-15-1	-5.104	-0.34	-1.477	-0.31	-23.709
Zidovudine (Retrovir)	267.241	30516-87-1	-5.102	-0.269	-0.639	-0.546	-30.687
Abscisic Acid (Dormin)	264.317	21293-29-8	-5.099	-0.268	-0.919	-0.182	-28.739
Pilocarpine HCl	244.718	54-71-7	-5.098	-0.34	-0.671	-0.403	-23.896
Ethinyl Estradiol	296.403	57-63-6	-5.097	-0.232	-1.326	-0.16	-26.557
Topiramate	339.362	97240-79-4	-5.097	-0.232	-0.367	-0.539	-25.408
Ponatinib (AP24534)	532.559	943319-70-8	-5.096	-0.131	-2.123	-0.32	-45.12
Carbazochrome sodium sulfonate	322.273	51460-26-5	-5.093	-0.255	-0.431	-0.446	-23.805
Maltitol	344.312	585-88-6	-5.092	-0.221	-0.565	0	-19.588
Trapidil	205.26	15421-84-8	-5.091	-0.339	-0.339	-0.26	-26.102
Adrucil (Fluorouracil)	130.077	51-21-8	-5.09	-0.566	-0.5	-0.16	-17.429
Tauroursodeoxycholic Acid	499.704	14605-22-2	-5.089	-0.15	-0.926	-0.295	-32.934
ARN-509	477.435	956104-40-8	-5.089	-0.154	-1.195	-0.31	-43.554
Combretastatin A4	316.348	117048-59-6	-5.088	-0.221	-1.192	-0.202	-34.694
Phenformin hydrochloride	241.721	834-28-6	-5.086	-0.339	-1.169	-0.997	-22.327
Rimantadine (Flumadine)	179.302	13392-28-4	-5.085	-0.391	-1.26	-0.272	-17.225
Vilazodone Hydrochloride	477.986	163521-08-2	-5.083	-0.154	-1.317	-0.32	-39.455
Etidronate (Didronel)	206.028	2809-21-4	-5.083	-0.462	-0.299	0	-16.032
Enalaprilat dihydrate	384.424	84680-54-6	-5.083	-0.203	-0.89	-0.044	-34.563
Eflornithine hydrochloride hydrate	236.645	96020-91-6	-5.082	-0.423	-0.399	-0.48	-7.186
Amfebutamone (Bupropion) HCl	276.202	31677-93-7	-5.081	-0.318	-1.563	-0.316	-26.407
Clorprenaline HCL	250.165	6933-90-0	-5.08	-0.363	-1.217	-0.304	-21.381
Ondansetron hydrochloride (Zofran)	329.824	99614-01-4	-5.078	-0.231	-0.945	-0.125	-32.956

Everolimus (RAD001)	958.224	159351-69-6	-5.078	-0.075	-1.051	-0.174	-47.24
Terpin (hydrate)	190.28	201256	-5.077	-0.423	-1.139	-0.32	-20.92
Chlormadinone acetate	404.927	302-22-7	-5.077	-0.181	-1.383	-0.32	-40.473
Fluconazole	306.271	86386-73-4	-5.07	-0.23	-0.881	-0.185	-29.554
Mycophenolate mofetil (CellCept)	433.495	128794-94-5	-5.069	-0.164	-1.317	-0.417	-39.459
Sulfadiazine	250.277	68-35-9	-5.068	-0.298	-0.864	-0.307	-22.294
Aniracetam	219.237	72432-10-1	-5.067	-0.317	-0.928	-0.41	-24.631
Guanfacine Hydrochloride	282.554	29110-48-3	-5.067	-0.338	-0.325	-0.525	-22.419
Cysteamine HCl	113.61	156-57-0	-5.067	-1.267	-0.354	-0.72	-9.347
Meropenem	383.462	96036-03-2	-5.066	-0.195	-0.653	-0.243	-38.295
Daclatasvir (BMS-790052)	738.875	1009119-64-5	-5.064	-0.094	-1.731	0	-57.299
Cephapirin Benzathine	1087.27	97468-37-6	-5.063	-0.181	-1.52	0	-45.784
4-Aminobenzoic acid	137.136	150-13-0	-5.062	-0.506	-0.595	-0.3	-15.416
Clindamycin HCl	461.444	21462-39-5	-5.062	-0.187	-1.274	0	-34.306
Dexrazoxane Hydrochloride	304.73	149003-01-0	-5.059	-0.266	-0.385	-0.692	-28.085
Pefloxacin mesylate	429.463	70458-95-6	-5.059	-0.211	-1.186	-0.015	-32.468
Pefloxacin Mesylate Dihydrate	465.494	149676-40-4	-5.059	-0.211	-1.159	-0.013	-32.432
Cefazedone	548.443	56187-47-4	-5.058	-0.153	-0.829	0	-54.649
Agomelatine	243.301	138112-76-2	-5.058	-0.281	-1.139	-0.492	-29.639
Tyloxapol	789.177	25301-02-4	-5.057	-0.089	-1.349	-0.453	-52.305
Isosorbide Mononitrate	191.139	16051-77-7	-5.057	-0.389	-0.783	-0.253	-22.445
Difluprednate	508.551	23674-86-4	-5.056	-0.14	-1.856	-0.108	-37.749
Fimasartan	501.646	247257-48-3	-5.054	-0.14	-1.623	-0.421	-44.496
Varenicline tartrate	361.349	375815-87-5	-5.053	-0.316	-1.531	0	-25.536
cephapirin sodium	445.445	24356-60-3	-5.053	-0.18	-1.305	-0.395	-39.754
Doxercalciferol (Hectorol)	412.648	54573-75-0	-5.051	-0.168	-1.986	-0.267	-29.613
Netilmicin Sulfate	1441.551	7664-93-9	-5.05	-0.153	-1.274	-0.45	-31.709
Acyclovir (Aciclovir)	225.205	59277-89-3	-5.049	-0.316	-0.53	-0.285	-19.612
Disodium Cromoglycate	512.33	15826-37-6	-5.048	-0.148	-1.572	-0.152	-41.367
Cloxiquine	179.603	130-16-5	-5.047	-0.421	-0.861	-0.16	-19.313
Glyburide (Glibenclamide)	494.004	10238-21-8	-5.044	-0.153	-2.119	-0.32	-40.227
Trenbolone acetate	312.403	10161-34-9	-5.041	-0.219	-0.931	-0.319	-32.782
Formestane	302.408	566-48-3	-5.04	-0.229	-0.98	-0.214	-30.757
Gabapentin Hydrochloride	207.698	60142-95-2	-5.033	-0.419	-0.708	-0.514	-15.24
Zolmitriptan (Zomig)	287.357	139264-17-8	-5.033	-0.24	-0.942	-0.679	-27.956

Primaquine Diphosphate	455.337	63-45-6	-5.029	-0.265	-1.698	-0.608	-27.931
Guanabenz (WY-8678) Acetate	291.134	23256-50-0	-5.028	-0.359	-1.15	-0.508	-25.061
Indigo	262.263	482-89-3	-5.028	-0.251	-1.163	-0.313	-29.153
Clindamycin	424.983	18323-44-9	-5.027	-0.186	-1.25	0	-34.345
Gabapentin (Neurontin)	171.237	60142-96-3	-5.027	-0.419	-0.686	-0.523	-15.182
Irsogladine	256.091	57381-26-7	-5.025	-0.314	-0.395	-0.391	-30.363
Oseltamivir Phosphate	410.4	204255-11-8	-5.025	-0.228	-1.192	-0.619	-27.678
Lansoprazole	369.361	103577-45-3	-5.023	-0.201	-1.39	0	-35.014
Chlorocresol	142.583	59-50-7	-5.021	-0.558	-0.905	-0.15	-14.437
Tebipenem pivoxil (L-084)	497.628	161715-24-8	-5.021	-0.152	-1.213	-0.456	-43.714
Glycocholic acid	465.623	475-31-0	-5.021	-0.152	-0.688	-0.622	-33.621
Ramelteon	259.343	196597-26-9	-5.02	-0.264	-1.709	-0.32	-25.303
Olanzapine (Zyprexa)	312.433	132539-06-1	-5.02	-0.228	-1.71	0	-33.221
Droperidol	379.427	548-73-2	-5.018	-0.179	-1.644	-0.16	-33.903
Cephalothin	418.42	58-71-9	-5.017	-0.193	-1.442	-0.524	-36.26
Efonidipine	631.655	111011-63-3	-5.014	-0.111	-1.954	-0.32	-46.354
MK-2048	461.874	869901-69-9	-5.009	-0.157	-1.346	-0.196	-45.204
Guaiazulene	198.303	489-84-9	-5.008	-0.334	-1.641	0	-21.842
S-(+)-Rolipram	275.343	85416-73-5	-5.005	-0.25	-1.335	-0.431	-27.138
Atomoxetine HCl	291.816	82248-59-7	-5.003	-0.263	-2.134	-0.437	-29.31
Leflunomide	270.207	75706-12-6	-5.003	-0.263	-0.859	-0.16	-25.653
Quinidine sulfate	782.943	6591-63-5	-4.997	-0.208	-1.524	-0.207	-31.645
carbadox	262.221	1791337	-4.996	-0.263	-1.264	-0.16	-24.915
Tivozanib (AV-951)	454.863	475108-18-0	-4.992	-0.156	-1.969	-0.38	-47.934
Chlorzoxazone	169.565	95-25-0	-4.991	-0.454	-0.773	-0.32	-20.501
Ipratropium bromide	412.361	22254-24-6	-4.991	-0.208	-1.586	0	-29.129
Rifapentine	877.031	61379-65-5	-4.988	-0.079	-1.44	0	-47.422
Dutasteride	528.53	164656-23-9	-4.988	-0.135	-1.271	0	-48.727
Benazepril hydrochloride	460.95	86541-74-4	-4.988	-0.161	-0.949	-0.427	-36.223
	294.391	118-10-5	-4.986	-0.227	-1.465	-0.177	-33.364
Broxyquinoline	302.95	521-74-4	-4.984	-0.383	-1.23	-0.32	-25.283
Phenytoin (Lepitoin)	252.268	57-41-0	-4.984	-0.262	-0.92	-0.454	-26.931
Iminostilbene	193.244	256-96-2	-4.984	-0.332	-1.494	0	-24.112
Medroxyprogesterone acetate	386.524	71-58-9	-4.981	-0.178	-1.18	-0.32	-40.649
Yohimbine hydrochloride (Antagonil)	390.904	65-19-0	-4.98	-0.192	-1.588	0	-33.313

L-Glutamine	146.144	56-85-9	-4.977	-0.498	-0.137	-0.94	-13.908
Albendazole (Albenza)	265.331	54965-21-8	-4.976	-0.276	-1.388	-0.296	-29.78
Timolol Maleate	432.492	26921-17-5	-4.973	-0.237	-1.314	-0.576	-33.381
Loperamide hydrochloride	513.498	34552-83-5	-4.972	-0.146	-1.59	-0.456	-42.228
Methimazole (Tapazole, Northyx)	114.169	60-56-0	-4.969	-0.71	-0.427	-0.159	-17.498
Aceclofenac	354.185	89796-99-6	-4.969	-0.216	-0.934	-0.195	-30.651
ATP (Adenosine-Triphosphate)	551.145	987-65-5	-4.964	-0.16	-0.546	0	-37.956
Methylprednisolone	374.471	83-43-2	-4.962	-0.184	-0.758	-0.3	-32.619
Benznidazole	260.249	22994-85-0	-4.962	-0.261	-0.834	-0.269	-29.865
Propacetamol hydrochloride	300.781	66532-86-3	-4.959	-0.261	-1.288	-0.366	-28.34
Flurbiprofen	244.261	51543-39-6	-4.957	-0.275	-1.147	-0.121	-26.158
Tinidazole	247.272	19387-91-8	-4.956	-0.31	-0.629	0	-25.474
Aclidinium Bromide	564.555	320345-99-1	-4.952	-0.15	-1.603	-0.097	-39.256
Homatropine Methylbromide	370.281	80-49-9	-4.95	-0.236	-1.194	0	-27.903
Carvedilol	406.474	72956-09-3	-4.949	-0.165	-2.08	-0.013	-39.24
Imidapril HCl	441.906	89396-94-1	-4.948	-0.171	-1.309	-0.012	-42.986
Conivaptan HCl (Vaprisol)	535.035	168626-94-6	-4.947	-0.13	-1.551	-0.202	-49.688
Teneligliptin hydrobromide	426.578	906093-29-6	-4.947	-0.165	-1.828	-0.283	-40.809
Cinchonidine	294.391	485-71-2	-4.941	-0.225	-1.497	-0.16	-30.569
Altrenogest	310.43	850-52-2	-4.94	-0.215	-1.583	-0.16	-23.396
Ticagrelor	522.568	274693-27-5	-4.939	-0.137	-0.888	-0.024	-36.85
Tizanidine HCl	290.172	64461-82-1	-4.938	-0.309	-1.331	-0.174	-28.265
Lynestrenol	284.436	52-76-6	-4.937	-0.235	-1.994	0	-30.95
Sodium benzoate	144.103	532-32-1	-4.935	-0.548	-1.144	-0.153	-12.831
Histamine 2HCl	184.067	56-92-8	-4.934	-0.617	-0.336	-0.46	-12.702
Formoterol hemifumarate	804.882	43229-80-7	-4.934	-0.197	-0.883	-0.612	-30.575
Hydroxyprogesterone caproate	428.604	630-56-8	-4.933	-0.159	-2.154	-0.256	-34.493
Doxycycline hyclate	1025.875	24390-14-5	-4.927	-0.154	-0.365	-0.191	-34.07
Aspirin (Acetylsalicylic acid)	180.157	50-78-2	-4.925	-0.379	-0.497	-0.277	-22.848
Olopatadine hydrochloride (Opatanol)	373.873	140462-76-6	-4.925	-0.197	-1.512	0	-31.045
Coumarin	146.143	91-64-5	-4.922	-0.447	-1.097	-0.193	-20.838
Plerixafor 8HCl (DB06809)	794.469	155148-31-5	-4.922	-0.137	-1.438	-0.456	-48.976
Halofuginone	414.681	55837-20-2	-4.919	-0.205	-0.844	-0.435	-34.287
Florfenicol	358.213	73231-34-2	-4.919	-0.234	-0.534	-0.304	-29.201
PF-06463922	406.413	1454846-35-5	-4.916	-0.164	-1.048	-0.16	-40.642

Dihydroartemisinin (DHA)	284.348	71939-50-9	-4.916	-0.246	-0.98	0	-28.817
Prednisone (Adasone)	358.428	19420	-4.915	-0.189	-0.886	-0.152	-29.5
Hydrocortisone (Cortisol)	362.46	50-23-7	-4.913	-0.189	-0.854	-0.145	-29.414
Prednisolone	360.444	50-24-8	-4.913	-0.189	-0.71	-0.145	-29.668
Tranylcypromine (2-PCPA) HCl	169.651	4548-34-9	-4.913	-0.491	-1.375	-0.375	-17.527
Honokiol	266.334	35354-74-6	-4.913	-0.246	-1.654	-0.464	-27.725
Furaltadone HCl	360.75	3759-92-0	-4.911	-0.214	-0.658	-0.323	-31.945
Valdecoxib	314.359	181695-72-7	-4.906	-0.223	-0.739	-0.265	-32.507
Hydroxychloroquine Sulfate	433.95	747-36-4	-4.905	-0.213	-1.747	-0.412	-31.983
Luliconazole	354.277	187164-19-8	-4.904	-0.234	-1.587	-0.22	-36.423
Loteprednol etabonate	466.952	82034-46-6	-4.902	-0.153	-1.695	-0.476	-37.79
(+)-Borneol	154.249	464-43-7	-4.9	-0.445	-1.5	0	-20.742
Voriconazole	349.31	137234-62-9	-4.9	-0.196	-1.035	-0.16	-33.147
Hydroquinidine	326.433	1435-55-8	-4.899	-0.204	-1.367	-0.16	-32.044
Dantrolene sodium hemiheptahydrate	326.433	24868-20-0	-4.899	-0.204	-1.367	-0.16	-32.044
Dropropizine	236.31	17692-31-8	-4.898	-0.288	-1.579	-0.456	-24.847
Marbofloxacin	362.356	115550-35-1	-4.898	-0.188	-1.162	0	-35.41
Borneol	154.249	507-70-0	-4.898	-0.445	-1.497	0	-20.707
Rofecoxib (Vioxx)	314.356	N/A	-4.898	-0.223	-1.205	-0.195	-33.476
Palonosetron HCl	332.868	135729-62-3	-4.896	-0.223	-1.44	0	-33.144
Triptolide	360.401	38748-32-2	-4.891	-0.188	-0.911	-0.046	-26.014
Calcitriol (Rocaltrol)	416.636	32222-06-3	-4.89	-0.163	-1.918	-0.442	-28.984
Naratriptan HCl	371.925	143388-64-1	-4.89	-0.213	-0.645	-0.435	-30.095
Clonidine hydrochloride (Catapres)	266.555	4205-91-8	-4.89	-0.349	-1.412	-0.16	-22.379
Penfluridol	523.965	26864-56-2	-4.887	-0.136	-1.799	-0.16	-39.971
Bethanechol chloride	196.675	590-63-6	-4.885	-0.444	-0.716	-0.332	-14.684
bendroflumethiazide	421.415	73-48-3	-4.885	-0.181	-1.044	-0.046	-35.103
Terazosin HCl Dihydrate	459.924	70024-40-7	-4.88	-0.174	-1.279	-0.41	-41.311
Eltrombopag olamine	564.633	496775-62-3	-4.88	-0.148	-1.229	-0.304	-43.08
Lercanidipine (hydrochloride)	648.188	132866-11-6	-4.879	-0.108	-1.759	-0.033	-55.107
Pyridostigmine Bromide (Mestinon)	261.116	101-26-8	-4.879	-0.375	-0.952	-0.215	-21.49
Crystal violet	407.979	548-62-9	-4.876	-0.174	-0.786	0	-34.651
Sertraline HCl	342.691	79559-97-0	-4.872	-0.244	-1.493	-0.16	-30.26
Azilsartan Medoxomil (TAK-491)	568.534	863031-21-4	-4.871	-0.116	-1.332	-0.595	-53.938
Ornidazole	219.626	16773-42-5	-4.87	-0.348	-0.789	0	-22.796

(-)-Tetramisole	240.752	16595-80-5	-4.868	-0.348	-1.309	0	-25.907
Methoxyphenamine Hydrochloride	215.72	1347292	-4.867	-0.374	-0.813	-0.587	-18.231
Sulfamethizole (Proklar)	270.331	144-82-1	-4.866	-0.286	-1.241	-0.172	-31.426
Epalrestat	319.399	82159-09-9	-4.865	-0.232	-0.561	-0.317	-26.042
(+)-Camphor	152.233	464-49-3	-4.863	-0.442	-1.39	0	-20.473
Asenapine maleate	401.84	65576-45-6, 1358	-4.862	-0.243	-1.334	-0.187	-28.486
Dextromethorphan (hydrobromide hydrate)	370.324	6700-34-1	-4.86	-0.243	-1.263	-0.259	-25.74
Doxazosin mesylate	547.581	77883-43-3	-4.859	-0.147	-1.419	-0.398	-44.58
Glimepiride	490.616	93479-97-1	-4.858	-0.143	-1.448	-0.32	-44.662
Avobenzone (Parsol 1789)	310.387	70356-09-1	-4.858	-0.211	-1.105	-0.636	-31.235
Methyl 4-hydroxybenzoate	152.147	99-76-3	-4.857	-0.442	-0.829	-0.32	-19.065
Tetracycline hydrochloride	480.896	64-75-5	-4.855	-0.152	-0.951	-0.16	-40.013
Chlorpropamide	276.74	94-20-2	-4.854	-0.286	-1.334	-0.317	-31.484
Emtricitabine	247.247	143491-57-0	-4.851	-0.303	-0.589	-0.247	-25.527
2-Deoxy-D-glucose	164.156	154-17-6	-4.851	-0.441	-0.55	0	-16.044
Linagliptin (BI-1356)	472.542	668270-12-0	-4.849	-0.139	-0.826	-0.594	-45.071
Oxymatrine (Matrine N-oxide)	264.363	16837-52-8	-4.848	-0.255	-1.211	0	-31.444
Ouabain	728.775	630-60-4	-4.847	-0.118	-0.586	0	-32.405
Sivelestat sodium tetrahydrate	528.506	201677-61-4	-4.845	-0.162	-1.662	-0.253	-35.611
Sclareol	308.499	515-03-7	-4.844	-0.22	-1.127	-0.29	-24.18
Nabumetone	228.286	42924-53-8	-4.844	-0.285	-1.078	-0.225	-22.117
Buparvaquone	326.429	88426-33-9	-4.843	-0.202	-1.39	-0.087	-32.382
Demeclocycline HCl	501.314	64-73-3	-4.842	-0.151	-1.012	-0.148	-28.023
Sulfadoxine (Sulphadoxine)	310.329	2447-57-6	-4.841	-0.231	-1.146	-0.291	-30.969
Flupenthixol dihydrochloride	507.439	51529-01-2	-4.84	-0.161	-1.115	-0.548	-31.537
Fexofenadine HCl	538.117	153439-40-8	-4.84	-0.131	-1.372	-0.361	-36.105
mesoridazine besylate	544.749	32672-69-8	-4.839	-0.186	-1.804	0	-41.414
Pirfenidone	185.222	53179-13-8	-4.839	-0.346	-1.368	-0.155	-24.915
Diphemanil Methylsulfate	389.508	62-97-5	-4.836	-0.23	-1.372	0	-25.052
Apramycin Sulfate	637.656	65710-07-8	-4.835	-0.131	-0.695	-0.588	-32.502
Etodolac (Lodine)	287.354						
-0.396	0	-0.303	-0.094	-0.014	-0.042	-0.192	4.837
Tetrahydropalmatine hydrochloride	391.888	6024-85-7	-4.831	-0.186	-1.321	-0.32	-32.133
Tropicamide	284.353	1508-75-4	-4.831	-0.23	-1.15	-0.564	-31.118
Oxybenzone	228.243	131-57-7	-4.83	-0.284	-1.443	-0.16	-28.423

Dehydrocostus Lactone	230.302	477-43-0	-4.83	-0.284	-1	-0.067	-26.708
Sulisobenzone	308.306	4065-45-6	-4.83	-0.23	-0.377	0	-22.865
Fomepizole (Antizol)	82.104	7554-65-6	-4.828	-0.805	-0.785	-0.32	-12.901
Eltrombopag	442.467	496775-61-2	-4.826	-0.146	-1.066	-0.304	-42.119
Megestrol Acetate	384.508	595-33-5	-4.825	-0.172	-1.142	-0.327	-33.846
Dexmedetomidine	200.28	113775-47-6	-4.824	-0.322	-1.456	-0.259	-24.373
Sucralose	397.633	56038-13-2	-4.822	-0.21	-1.015	0	-27.002
Lopinavir (ABT-378)	628.801	192725-17-0	-4.821	-0.105	-1.757	-0.178	-50.442
Neostigmine bromide (Prostigmin)	303.195	114-80-7	-4.821	-0.301	-0.749	-0.074	-25.137
Desloratadine	310.821	100643-71-8	-4.82	-0.219	-1.152	-0.205	-33.267
Lamotrigine	256.091	84057-84-1	-4.82	-0.301	-0.7	-0.243	-29.057
Nicarbazin	426.383	330-95-0	-4.818	-0.219	-0.668	-0.203	-34.511
Mequinol	124.137	150-76-5	-4.815	-0.535	-0.752	-0.16	-15.199
Estrone	270.366	53-16-7	-4.814	-0.241	-0.64	-0.544	-28.249
Pyrimethamine	248.711	58-14-0	-4.814	-0.283	-0.804	-0.307	-26.533
(-)-Menthol	156.265	2216-51-5	-4.813	-0.438	-1.034	-0.16	-16.023
Torsemide (Demadex)	348.42	56211-40-6	-4.812	-0.201	-1.644	-0.247	-36.985
Piperazine	86.136	110-85-0	-4.807	-0.801	-0.566	-0.601	-10.282
Meptazinol HCl	269.81	59263-76-2	-4.806	-0.283	-1.192	-0.179	-24.993
Ketoconazole	531.431	65277-42-1	-4.806	-0.133	-1.153	-0.29	-47.472
Sulfamethoxazole	253.278	723-46-6	-4.805	-0.283	-1.062	-0.16	-28.109
Pregnenolone	316.478	145-13-1	-4.804	-0.209	-1.3	-0.165	-34.487
Capreomycin Sulfate	750.785	1405-37-4	-4.801	-0.104	-0.705	-0.353	-47.879
Sulfamethazine	278.33	57-68-1	-4.8	-0.253	-1.325	-0.122	-30.388
Naproxen Sodium	252.241	26159-34-2	-4.799	-0.282	-1.403	-0.177	-23.396
Benzocaine hydrochloride	201.65	23239-88-5	-4.799	-0.4	-1.504	-0.32	-22.15
Rotundine	355.428	483-14-7	-4.798	-0.185	-1.404	-0.284	-32.2
Bromfenac Sodium	356.147	91714-93-1	-4.797	-0.24	-1.381	-0.16	-32.36
Nimesulide	308.31	51803-78-2	-4.797	-0.228	-1.286	0	-31.927
Sulfamonomethoxine	280.303	1220-83-3	-4.796	-0.252	-1.009	-0.388	-29.651
Clioquinol	305.5	130-26-7	-4.795	-0.369	-1.21	-0.32	-25.376
Darunavir Ethanolate (Prezista)	593.732	635728-49-3	-4.791	-0.126	-1.031	-0.576	-35.271
Probenecid (Benemid)	285.359	57-66-9	-4.79	-0.252	-0.974	0	-29.222
Amfenac Sodium (monohydrate)	295.266	61618-27-7	-4.788	-0.252	-1.315	-0.16	-29.213
Duloxetine HCl (Cymbalta)	333.875	136434-34-9	-4.788	-0.228	-1.808	-0.25	-31.748

Tropisetron HCl	320.814	105826-92-4	-4.788	-0.228	-0.95	-0.194	-22.577
Liothyronine Sodium	672.955	20241	-4.788	-0.208	-1.717	-0.431	-38.404
Asunaprevir	748.286	630420-16-5	-4.787	-0.094	-1.315	-0.008	-59.73
Cholic acid	408.571	81-25-4	-4.786	-0.165	-0.62	-0.392	-26.763
Hydroquinone	110.111	123-31-9	-4.786	-0.598	-0.748	-0.16	-13.216
AlogliptinثذSYR-ثر322	339.392	850649-61-5	-4.781	-0.191	-1.19	-0.16	-33.764
Alogliptin	339.392	850649-61-5	-4.781	-0.191	-1.19	-0.16	-33.764
Salmeterol Xinafoate	603.745	94749-08-3	-4.777	-0.159	-2.074	-0.348	-35.175
Spectinomycin 2HCl	405.271	21736-83-4	-4.775	-0.208	-0.404	-0.495	-22.997
MDV3100 (Enzalutamide)	464.436	915087-33-1	-4.774	-0.149	-0.827	-0.179	-44.436
Isoprenaline hydrochloride	247.719	51-30-9	-4.773	-0.318	-0.778	-0.147	-21.78
Amprolium HCl	315.241	137-88-2	-4.773	-0.265	-0.612	-0.16	-27.41
Dexmedetomidine HCl (Precedex)	236.74	145108-58-3	-4.771	-0.318	-1.384	-0.241	-24.037
Isoconazole nitrate (Travogen)	479.141	24168-96-5	-4.77	-0.191	-1.374	-0.316	-38.7
Vemurafenib (PLX4032, RG7204)	489.922	918504-65-1	-4.769	-0.145	-1.762	-0.156	-44.932
Oxibendazole	249.266	20559-55-1	-4.768	-0.265	-0.73	-0.243	-30.281
Ixazomib (MLN2238)	361.029	1072833-77-2	-4.765	-0.207	-1.161	-0.144	-30.212
Nifuroxazide	275.217	965-52-6	-4.765	-0.238	-0.785	-0.505	-28.69
Biotin (Vitamin B7)	244.311	58-85-5	-4.765	-0.298	-0.813	-0.167	-25.003
Methazolamide	236.272	554-57-4	-4.762	-0.34	-0.317	-0.16	-27.389
(-)-Borneol	154.249	464-45-9	-4.761	-0.433	-0.904	-0.16	-17.525
Fluvastatin sodium (Lescol)	433.448	93957-55-2	-4.761	-0.159	-1.549	-0.32	-33.232
Sodium Picosulfate	481.407	10040-45-6	-4.76	-0.164	-1.471	-0.293	-26.828
Amiodarone HCl	681.773	19774-82-4	-4.758	-0.153	-1.11	-0.353	-47.371
Riociguat (BAY 63-2521)	422.416	625115-55-1	-4.757	-0.153	-1.456	-0.35	-37.663
Sulfamerazine	264.304	127-79-7	-4.757	-0.264	-0.805	-0.263	-29.001
Sulconazole Nitrate	460.762	82382-23-8	-4.755	-0.198	-1.284	-0.294	-39.157
Vortioxetine (Lu AA21004) hydrobromide	379.358	960203-27-4	-4.754	-0.226	-1.328	-0.292	-30.804
Rolipram	275.343	61413-54-5	-4.752	-0.238	-1.266	-0.288	-26.241
Sulfacetamide Sodium	236.223	127-56-0	-4.751	-0.339	-1.308	-0.323	-21.928
Pantoprazole (Protonix)	383.37	102625-70-7	-4.748	-0.183	-0.857	-0.16	-34.372
Drostanolone Propionate	360.53	521-12-0	-4.747	-0.183	-1.38	-0.258	-25.967
Aztreonam (Azactam, Cayston)	435.433	78110-38-0	-4.747	-0.17	-0.687	-0.009	-35.966
Pergolide mesylate	410.594	66104-23-2	-4.746	-0.216	-1.565	-0.147	-29.449
Carboplatin	371.248	41575-94-4	-4.744	-0.474	-0.111	-0.32	-7.504

carbachol	182.649	51-83-2	-4.74	-0.474	-0.535	-0.295	-9.972
Griseofulvin	352.766	126-07-8	-4.74	-0.198	-1.417	0	-36.568
Troxipide	294.346	30751-05-4	-4.74	-0.226	-0.858	-0.451	-30.082
Fenoprofen calcium	522.602	34597-40-5	-4.74	-0.263	-1.495	-0.159	-23.989
Ambroxol HCl	414.564	23828-92-4	-4.738	-0.263	-1.138	-0.456	-33.641
Toltrazuril	425.382	69004-03-1	-4.737	-0.163	-1.102	-0.262	-39.591
Hexylresorcinol	194.27	136-77-6	-4.737	-0.338	-1.565	-0.456	-21.864
Olmutinib (HM61713, BI 1482694)	486.589	1353550-13-6	-4.735	-0.135	-1.186	-0.339	-45.355
Protionamide (Prothionamide)	180.27	14222-60-7	-4.733	-0.394	-0.773	-0.16	-20.582
Tianeptine sodium	458.934	30123-17-2	-4.729	-0.163	-2.063	-0.16	-29.46
Tazarotene (Avage)	351.462	118292-40-3	-4.729	-0.189	-1.618	-0.258	-38.333
Ketanserin (Vulketan Gel)	395.427	74050-98-9	-4.729	-0.163	-1.331	-0.279	-38.902
Entacapone	305.286	130929-57-6	-4.727	-0.215	-0.372	-0.261	-27.192
Triamcinolone (Aristocort)	394.434	124-94-7	-4.725	-0.169	-0.347	-0.109	-18.374
Sulfapyridine (Dagenan)	249.289	144-83-2	-4.724	-0.278	-0.826	-0.264	-26.631
Ziprasidone hydrochloride	449.397	122883-93-6	-4.723	-0.169	-1.26	-0.333	-39.514
Streptozotocin (STZ)	265.221	18883-66-4	-4.722	-0.262	-0.316	0	-24.417
Ciprofibrate	289.154	52214-84-3	-4.722	-0.262	-1.262	0	-26.451
Ruxolitinib (INCB018424)	306.365	941678-49-5	-4.722	-0.205	-1.137	-0.477	-30.133
Carbenicillin disodium	422.363	4800-94-6	-4.721	-0.182	-0.153	-0.435	-19.672
Camphor	152.233	76-22-2	-4.719	-0.429	-0.777	-0.115	-18.961
Ligustrazine hydrochloride	172.655	76494-51-4	-4.719	-0.472	-1.026	-0.137	-19.762
Rizatriptan Benzoate (Maxalt)	391.466	145202-66-0	-4.718	-0.236	-0.504	-0.306	-27.913
Moguisteine	339.407	119637-67-1	-4.717	-0.205	-1.392	-0.608	-35.776
Ivabradine HCl (Procoralan)	505.046	148849-67-6	-4.716	-0.139	-1.65	-0.24	-49.684
Vinorelbine Tartrate	1079.106	125317-39-7	-4.716	-0.083	-1.453	-0.246	-48.188
Nikethamide	178.231	59-26-7	-4.714	-0.363	-0.887	-0.163	-22.822
Vinpocetine (Cavinton)	350.454	42971-09-5	-4.714	-0.181	-1.382	-0.053	-33.57
Granisetron HCl	348.87	107007-99-8	-4.71	-0.205	-1.036	-0.46	-28.459
Tetramisole HCl	240.752	5086-74-8, 5036-[illegible]	-4.71	-0.336	-1.355	0	-25.231
Dorzolamide HCL	360.901	130693-82-2	-4.709	-0.248	-0.647	-0.16	-29.577
Nitazoxanide (Alinia, Annita)	307.282	55981-09-4	-4.708	-0.224	-0.944	-0.32	-36.195
Pemirolast (BMY 26517) potassium	266.3	100299-08-9	-4.706	-0.277	-0.903	0	-29.71
Prucalopride Succinat	485.958	179474-85-2	-4.702	-0.188	-1.956	-0.168	-32.38
Monomethyl auristatin E (MMAE)	717.979	474645-27-7	-4.698	-0.092	-1.508	-0.263	-47.355

Closantel	663.074	57808-65-8	-4.698	-0.157	-1.461	-0.16	-47.951
Moexipril HCl	535.029	82586-52-5	-4.698	-0.13	-1.55	-0.32	-46.689
Synephrine (Oxedrine)	167.205	34520	-4.697	-0.391	-0.759	-0.403	-17.876
Tyrosol	138.164	501-94-0	-4.696	-0.47	-1.029	-0.614	-15.809
Nitrofurazone (Nitrofural)	198.136	59-87-0	-4.696	-0.335	-0.292	-0.16	-24.175
Fluocinonide (Vanos)	494.525	356-12-7	-4.693	-0.134	-1.011	-0.16	-42.57
Lomitapide Mesylate	789.826	202914-84-9	-4.692	-0.094	-1.448	0	-52.476
Lumefantrine	528.94	82186-77-4	-4.691	-0.134	-2.13	-0.16	-42.683
Lafutidine	431.548	118288-08-7	-4.689	-0.156	-2.383	-0.092	-41.678
Afloqualone	283.3	56287-74-2	-4.688	-0.223	-1.162	-0.038	-32.487
Dexamethasone acetate	434.498	1177-87-3	-4.687	-0.151	-1.005	-0.239	-40.46
Penicillin G Sodium	356.372	69-57-8	-4.685	-0.204	-0.87	-0.265	-29.084
Manidipine (Manyper)	610.699	89226-50-6	-4.684	-0.104	-1.483	-0.092	-56.602
Manidipine dihydrochloride (CV-4093)	683.621	89226-75-5	-4.684	-0.104	-1.483	-0.092	-56.602
Dacarbazine (DTIC-Dome)	182.183	891986	-4.683	-0.36	-0.443	-0.315	-24.381
Galanthamine	287.354	357-70-0	-4.682	-0.223	-0.724	-0.099	-25.175
Mezlocillin Sodium	561.564	42057-22-7	-4.682	-0.13	-0.736	-0.184	-50.065
Econazole nitrate (Spectazole)	444.696	24169-02-6	-4.681	-0.195	-1.218	-0.296	-37.867
Nefopam HCl	289.8	23327-57-3	-4.68	-0.246	-1.719	0	-25.645
Robenidine Hydrochoride	370.664	25875-50-7	-4.676	-0.213	-0.993	-0.16	-40.559
Clofibric acid	214.646	882-09-7	-4.676	-0.334	-1.045	-0.006	-24.105
Icotinib	391.42	610798-31-7	-4.674	-0.161	-1.523	-0.031	-39.508
Desvenlafaxine	263.375	93413-62-8	-4.673	-0.246	-1.186	-0.3	-24.405
Aripiprazole (Abilify)	448.385	129722-12-9	-4.669	-0.156	-1.541	-0.269	-44.403
Primidone (Mysoline)	218.252	125-33-7	-4.668	-0.292	-0.782	-0.159	-24.748
Sertaconazole nitrate	500.783	99592-39-9	-4.667	-0.173	-1.359	-0.315	-39.661
Butoconazole nitrate	474.789	64872-77-1	-4.666	-0.187	-1.24	-0.32	-35.691
Cefpirome sulfate	612.656	98753-19-6	-4.663	-0.133	-0.233	-0.545	-35.037
Telmisartan (Micardis)	514.617	144701-48-4	-4.66	-0.119	-1.212	0	-39.719
Diclofenac Diethylamine	369.285	78213-16-8	-4.658	-0.245	-1.053	-0.079	-31.991
Erdosteine	249.307	84611-23-4	-4.658	-0.311	-0.445	-0.584	-25.215
Umeclidinium bromide	508.49	869113-09-7	-4.657	-0.146	-1.487	0	-42.063
Detomidine HCl	222.714	90038-01-0	-4.656	-0.333	-1.057	-0.253	-21.832
Rosiglitazone HCl	393.888	302543-62-0	-4.652	-0.186	-1.092	-0.241	-33.23
Rosiglitazone (Avandia)	357.427	122320-73-4	-4.651	-0.186	-1.092	-0.241	-33.227

Desvenlafaxine Succinate	381.463	386750-22-7	-4.648	-0.245	-1.164	-0.299	-24.393
Glasdegib (PF-04449913)	374.439	1095173-27-5	-4.648	-0.166	-0.971	-0.32	-39.433
Naloxone HCl	363.835	357-08-4	-4.647	-0.194	-0.958	-0.15	-25.54
Parthenolide ((-)-Parthenolide)	248.318	20554-84-1	-4.647	-0.258	-1.068	0	-26.643
Apatinib mesylate	493.578	811803-05-1	-4.646	-0.155	-1.08	-0.16	-43.023
Urethane	89.093	51-79-6	-4.641	-0.773	-0.356	-0.351	-11.463
Dapson	248.301	80-08-0	-4.638	-0.273	-0.857	-0.32	-25.941
Lubiprostone	390.462	136790-76-6	-4.637	-0.172	-1.243	-0.651	-28.816
Phenazopyridine HCl	249.699	136-40-3	-4.637	-0.29	-1.194	-0.23	-27.032
Camostat Mesilate (FOY-305)	494.518	59721-29-8	-4.634	-0.16	-0.932	-0.569	-39.666
Roxithromycin (Roxl-150)	837.047	80214-83-1	-4.631	-0.08	-0.935	0	-49.613
Sulfameter (Bayrena)	280.303	651-06-9	-4.63	-0.244	-0.776	-0.32	-29.652
Ursodiol (Actigal Urso)	392.572	128-13-2	-4.629	-0.165	-0.414	-0.283	-21.834
Celecoxib	381.372	169590-42-5	-4.628	-0.178	-1.037	-0.16	-31.245
Etofibrate	363.792	31637-97-5	-4.621	-0.185	-1.213	-0.435	-40.569
Alfuzosin hydrochloride (Uroxatral)	425.91	81403-68-1	-4.62	-0.165	-0.926	-0.577	-41.933
Pralidoxime (chloride)	172.612	51-15-0	-4.619	-0.462	-0.73	-0.16	-17.204
(+/-)-Sulfinpyrazone	404.481	57-96-5	-4.619	-0.159	-1.291	0	-42.068
Nitrofurantoin	238.157	67-20-9	-4.618	-0.272	-0.142	-0.424	-28.354
Lithocholic acid	376.573	434-13-9	-4.616	-0.171	-0.673	-0.433	-25.351
Donepezil HCl (Aricept)	415.953	120011-70-3	-4.614	-0.165	-1.611	-0.32	-36.945
Laquinimod (ABR-215062)	356.803	248281-84-7	-4.613	-0.185	-1.607	-0.031	-37.275
Cefsulodin sodium	554.528	52152-93-9	-4.613	-0.128	-0.486	-0.32	-40.701
Ibuprofen Lysine (NeoProfen)	352.468	57469-77-9	-4.61	-0.307	-1.051	0	-21.094
Sulpiride	341.426	15676-16-1	-4.609	-0.2	-1.277	-0.07	-34.167
Dasatinib	488.006	302962-49-8	-4.609	-0.14	-1.447	-0.181	-45.495
Sulbactam sodium (Unasyn)	255.223	69388-84-7	-4.606	-0.307	-0.239	-0.077	-22.443
Acetohydroxamic acid	75.067	546-88-3	-4.605	-0.921	-0.115	-0.294	-11.448
Cyproheptadine hydrochloride (Periactin)	323.859	969-33-5	-4.603	-0.209	-1.636	0	-28.349
Gliquidone	527.632	33342-05-1	-4.6	-0.124	-1.456	0	-46.687
Tanshinone IIA (Tanshinone B)	294.344	568-72-9	-4.599	-0.209	-0.58	-0.438	-29.567
Levosulpiride (Levogastrol)	341.426	23672-07-3	-4.599	-0.2	-0.846	-0.167	-33.318
Betamethasone (Celestone)	392.461	378-44-9	-4.598	-0.164	-1.026	0	-28.689
Rivaroxaban (Xarelto)	435.881	366789-02-8	-4.598	-0.159	-1.406	-0.368	-39.214
Quinapril hydrochloride (Accupril)	474.977	82586-55-8	-4.596	-0.144	-1.507	-0.024	-39.737

Prucalopride	367.87	179474-81-8	-4.595	-0.184	-1.584	-0.373	-32.653
oxethazaine	467.643	126-27-2	-4.593	-0.135	-1.749	-0.176	-41.8
Pioglitazone (Actos)	356.439	111025-46-8	-4.592	-0.184	-1.217	-0.262	-32.061
Brexpiprazole	433.566	913611-97-9	-4.592	-0.148	-1.459	-0.021	-49.588
Sulfamethoxypyridazine	280.303	80-35-3	-4.592	-0.242	-1.161	-0.095	-30.193
Sumatriptan succinate	413.488	103628-48-4	-4.591	-0.23	-0.348	-0.443	-29.485
Nilvadipine (ARC029)	385.371	75530-68-6	-4.59	-0.164	-0.847	-0.009	-35.994
Tofacitinib citrate (CP-690550 citrate)	504.493	540737-29-9	-4.589	-0.2	-0.722	-0.174	-31.93
Tofacitinib (CP-690550, Tasocitinib)	312.37	477600-75-2	-4.589	-0.2	-0.722	-0.174	-31.93
Penicillamine (Cuprimine)	149.211	52-67-5	-4.588	-0.51	-0.528	-0.291	-16.022
MPEP	193.244	96206-92-7	-4.587	-0.306	-2.018	0	-24.7
mepenzolate bromide	420.34	76-90-4	-4.585	-0.183	-0.712	-0.11	-30.596
Lomerizine 2HCl	541.457	101477-54-7, 101	-4.585	-0.135	-1.35	-0.32	-40.529
Pioglitazone hydrochloride (Actos)	392.9	112529-15-4	-4.578	-0.183	-1.219	-0.23	-31.813
Gatifloxacin	375.394	112811-59-3	-4.567	-0.169	-1.396	-0.01	-37.405
Benzyl benzoate	212.244	120-51-4	-4.564	-0.285	-1.264	-0.203	-27.005
Rotenone (Barbasco)	394.417	83-79-4	-4.561	-0.157	-1.78	0	-35.785
Febuxostat (Uloric)	316.375	144060-53-7	-4.554	-0.207	-1.056	-0.145	-30.591
Trifluoperazine 2HCl	480.417	440-17-5	-4.554	-0.163	-1.84	0	-39.182
Levosimendan	280.285	141505-33-1	-4.55	-0.217	-0.41	-0.78	-33.058
Phenylephrine hydrochloride	203.666	61-76-7	-4.548	-0.379	-0.535	-0.325	-16.635
Olsalazine Sodium	346.203	6054-98-4	-4.545	-0.207	-0.062	-0.388	-21.757
Miconazole nitrate	479.141	22832-87-7	-4.541	-0.182	-1.239	-0.304	-39.214
Flupirtine maleate	420.392	75507-68-5	-4.541	-0.206	-1.56	-0.19	-35.184
Methacycline hydrochloride (Physiomycine)	478.88	3963-95-9	-4.54	-0.142	-1.03	-0.141	-35.416
Dacomitinib (PF299804, PF-00299804)	469.939	1110813-31-4	-4.54	-0.138	-1.579	-0.101	-43.303
AMG-073 HCl (Cinacalcet hydrochloride)	393.873	364782-34-3	-4.539	-0.175	-1.547	-0.305	-35.261
Lathyrol	334.45	34420-19-4	-4.538	-0.189	-0.696	0	-32.93
Rosiglitazone maleate	473.499	155141-29-0	-4.535	-0.181	-0.986	-0.281	-39.485
D-(+)-Trehalose dihydrate	378.327	6138-23-4	-4.534	-0.197	-0.356	0	-22.194
Dabrafenib (GSK2118436)	519.562	1195765-45-7	-4.534	-0.13	-0.682	-0.372	-44.323
Betaxolol hydrochloride (Betoptic)	343.889	63659-19-8	-4.533	-0.206	-1.72	-0.29	-30.129
Dienogest	311.418	65928-58-7	-4.533	-0.197	-0.785	-0.156	-33.23
pentoxifylline	278.307	1677687	-4.532	-0.227	-0.534	-0.246	-31.711
MetoclopraMide HCl	336.257	7232-21-5	-4.532	-0.227	-1.142	-0.607	-31.056

Tazobactam	300.291	89786-04-9, 8978	-4.532	-0.227	-0.132	0	-26.884
glafenine hydrochloride	409.263	65513-72-6	-4.529	-0.174	-1.503	-0.072	-37.717
Metronidazole (Flagyl)	171.154	443-48-1	-4.528	-0.377	-0.333	-0.265	-20.556
Zafirlukast (Accolate)	575.675	107753-78-6	-4.528	-0.11	-1.373	0	-49.941
Chlorobutanol	177.457	57-15-8	-4.528	-0.566	-0.711	-0.16	-16.098
Nortriptyline hydrochloride	299.838	894-71-3	-4.527	-0.226	-0.808	-0.441	-28.801
Promestriene	328.488	39219-28-8	-4.525	-0.189	-1.9	0	-30.735
Saxagliptin (BMS-477118,Onglyza)	315.41	361442-04-8	-4.524	-0.197	-1.244	-0.259	-34.185
Belinostat (PXD101)	318.348	414864-00-9	-4.522	-0.206	-0.526	-0.373	-33.09
Iloperidone (Fanapt)	426.481	133454-47-4	-4.522	-0.146	-1.316	0	-41.137
Fenofibrate (Tricor, Trilipix)	360.831	49562-28-9	-4.52	-0.181	-1.655	-0.209	-38.613
Lesinurad	404.281	878672-00-5	-4.52	-0.188	-0.725	-0.229	-36.645
Piperine (1-Piperoylpiperidine)	285.338	94-62-2	-4.514	-0.215	-0.901	-0.32	-29.412
Racecadotril (Acetorphan)	385.477	81110-73-8	-4.511	-0.167	-0.391	-0.678	-36.672
terfenadine	471.673	50679-08-8	-4.511	-0.129	-1.8	-0.386	-33.329
Cefazolin Sodium	476.489	27164-46-1	-4.511	-0.156	-1.107	-0.136	-46.904
Pancuronium dibromide	732.67	15500-66-0	-4.51	-0.11	-1.755	0	-34.759
Ulipristal acetate	475.619	126784-99-4	-4.505	-0.129	-1.451	-0.309	-34.404
Amlodipine besylate (Norvasc)	567.051	111470-99-6	-4.503	-0.161	-1.197	-0.602	-36.951
Tenofovir Alafenamide (GS-7340)	476.466	379270-37-8	-4.501	-0.136	-0.667	-0.309	-46.851
Trichlormethiazide (Achletin)	380.656	133-67-5	-4.5	-0.225	-0.51	-0.16	-30.141
Xylazine HCl	256.795	23076-35-9	-4.499	-0.3	-1.032	-0.16	-24.491
Cinnamic acid	148.159	140-10-3	-4.499	-0.409	-0.416	-0.243	-18.485
Ethopabate	237.252	21704	-4.498	-0.265	-0.657	-0.374	-25.604
Butenafine HCl	353.928	101827-46-7	-4.495	-0.187	-1.593	0	-37.847
Mevastatin	390.513	73573-88-3	-4.493	-0.16	-1.651	-0.409	-35.777
Miconazole (Monistat)	416.129	22916-47-8	-4.493	-0.18	-1.171	-0.307	-39.422
Dasatinib Monohydrate	506.021	863127-77-9	-4.492	-0.136	-1.351	-0.162	-38.794
Vitamin C (Ascorbic acid)	176.124	50-81-7	-4.492	-0.374	-0.323	0	-15.532
Amoxapine	313.781	14028-44-5	-4.489	-0.204	-0.889	-0.178	-30.721
Ospemifene	378.891	128607-22-7	-4.488	-0.166	-1.774	-0.32	-32.361
Cefonicid sodium	586.53	61270-78-8	-4.487	-0.128	-0.743	-0.284	-40.416
Ozagrel HCl	264.708	78712-43-3	-4.486	-0.264	-1.058	-0.157	-22.509
Ranolazine dihydrochloride	500.458	95635-56-6	-4.486	-0.145	-1.495	-0.456	-41.581
Reserpine	608.679	50-55-5	-4.484	-0.102	-1.637	0	-47.618

Almotriptan malate (Axert)	469.552	181183-52-8	-4.483	-0.195	-1.608	0	-33.276
Parecoxib	370.422	198470-84-7	-4.481	-0.172	-1.501	-0.018	-39.268
Raltegravir (MK-0518)	444.416	518048-05-0	-4.481	-0.14	-0.814	-0.3	-43.373
Irinotecan HCl Trihydrate (Campto)	677.185	136572-09-3	-4.479	-0.104	-1.132	-0.165	-46.563
Salicylanilide	213.232	87-17-2	-4.477	-0.28	-1.008	-0.192	-24.944
Trazodone hydrochloride (Desyrel)	408.325	25332-39-2	-4.477	-0.172	-1.091	0	-37.223
Desonide	416.507	638-94-8	-4.474	-0.149	-0.839	-0.152	-28.855
Urapidil hydrochloride	423.937	64887-14-5	-4.473	-0.16	-0.725	-0.142	-41.012
Eletriptan HBr	463.431	177834-92-3	-4.473	-0.166	-1.536	-0.084	-38.053
Carbimazole	186.232	22232-54-8	-4.473	-0.373	-0.283	-0.28	-28.972
Caryophyllene oxide	220.35	1139-30-6	-4.472	-0.28	-1.477	0	-20.868
Hydroxyurea (Cytodrox)	76.055	127-07-1	-4.472	-0.894	0	-0.292	-11.358
Clozapine (Clozaril)	326.823	5786-21-0	-4.469	-0.194	-1.227	-0.09	-34.73
Meclofenoxate (Centrophenoxine) HCl	294.174	3685-84-5	-4.468	-0.263	-0.717	-0.754	-25.202
Bisacodyl	361.391	30652-11-0	-4.465	-0.165	-1.201	-0.267	-36.117
Etomidate	244.289	33125-97-2	-4.465	-0.248	-1.738	0	-33.285
Pravastatin sodium	446.51	81131-70-6	-4.461	-0.149	-0.943	-0.304	-28.51
Sildenafil citrate	666.7	171599-83-0	-4.461	-0.135	-1.654	0	-43.736
Secnidazole (Flagentyl)	185.181	3366-95-8	-4.459	-0.343	-0.389	-0.253	-21.36
Glycopyrrolate	398.335	596-51-0	-4.457	-0.194	-0.702	-0.199	-29.113
Andrographolide	350.449	5508-58-7	-4.457	-0.178	-0.782	0	-18.758
Ozagrel	228.247	82571-53-7	-4.457	-0.262	-1.048	-0.156	-22.648
Cefotiam hydrochloride	598.55	66309-69-1	-4.455	-0.131	-0.819	-0.088	-45.731
bephenium hydroxynaphthoate	443.534	3818-50-6	-4.453	-0.234	-1.418	-0.243	-24.867
Ibuprofen (Advil)	206.281	15687-27-1	-4.452	-0.297	-1.183	0	-22.365
Aceglutamide	188.181	2490-97-3	-4.448	-0.342	-0.372	-0.488	-21.545
Perphenazine	403.969	58-39-9	-4.443	-0.165	-1.163	-0.16	-30.719
Pramipexole (Mirapex)	211.327	104632-26-0	-4.441	-0.317	-0.966	-0.304	-20.705
Estradiol Cypionate	396.562	313-06-4	-4.437	-0.153	-1.471	-0.32	-32.838
Azatadine dimaleate	522.546	3978-86-7	-4.435	-0.202	-1.096	0	-25.037
Oxyclozanide	401.457	2277-92-1	-4.435	-0.202	-1.27	-0.249	-35.891
Flumethasone	410.452	2135-17-3	-4.431	-0.153	-0.721	-0.152	-26.235
Chlorpromazine HCl	355.325	69-09-0	-4.431	-0.211	-1.335	0	-33.659
Moclobemide (Ro 111163)	268.739	71320-77-9	-4.428	-0.246	-1.478	0	-27.439
Mexiletine HCl	215.72	1267397	-4.427	-0.341	-1.122	-0.304	-16.644

Homatropine Bromide	356.255	51-56-9	-4.424	-0.221	-1.047	-0.012	-27.272
Suplatast tosylate	499.641	94055-76-2	-4.423	-0.201	-1.371	-0.32	-26.974
Trimetazidine dihydrochloride	339.258	13171-25-0	-4.422	-0.233	-1.065	-0.107	-27.967
Thioctic acid	206.326	1077-28-7	-4.419	-0.368	-1.144	-0.153	-20.962
Hyodeoxycholic acid (HDCA)	392.572	83-49-8	-4.416	-0.158	-0.389	-0.401	-26.351
Pizotifen malate	429.529	1201595	-4.414	-0.21	-1.545	0	-27.822
Hyoscyamine (Daturine)	289.369	101-31-5	-4.41	-0.21	-1.353	0	-28.035
Letrozole	285.303	112809-51-5	-4.409	-0.2	-0.619	-0.149	-32.845
Vandetanib (ZD6474)	475.354	443913-73-3	-4.408	-0.147	-1.312	-0.376	-36.803
Propantheline bromide	448.393	50-34-0	-4.406	-0.163	-1.156	-0.31	-32.192
Telaprevir (VX-950)	679.849	402957-28-2	-4.399	-0.09	-0.971	-0.096	-55.281
Mometasone furoate	521.429	83919-23-7	-4.398	-0.126	-1.212	-0.249	-37.377
Carfilzomib (PR-171)	719.91	868540-17-4	-4.396	-0.085	-1.528	0	-54.006
Chlorotrianisene	380.864	569-57-3	-4.394	-0.163	-1.784	-0.07	-35.751
Betulin	442.717	473-98-3	-4.393	-0.137	-1.607	-0.471	-26.634
Drospirenone	366.493	67392-87-4	-4.389	-0.163	-1.178	0	-36.911
Iron sucrose	736.059	8047-67-4	-4.387	-0.313	-0.482	-0.212	-14.281
Protopine	353.369	130-86-9	-4.387	-0.169	-1.191	0	-31.297
Thioridazine hydrochloride	407.035	130-61-0	-4.386	-0.175	-1.576	0	-37.391
Nifedipine (Adalat)	346.335	21829-25-4	-4.386	-0.175	-1.059	0	-37.574
Repaglinide	452.586	135062-02-1	-4.386	-0.133	-0.918	-0.277	-42.416
Gliclazide (Diamicron)	323.411	21187-98-4	-4.385	-0.199	-1.243	-0.304	-26.627
Levamlodipine	408.876	103129-82-4	-4.385	-0.157	-1.552	-0.322	-38.582
Vorapaxar (SCH 530348)	492.582	618385-01-6	-4.384	-0.122	-1.192	-0.243	-43.563
Sivelestat (ONO-5046)	434.463	127373-66-4	-4.383	-0.146	-1.164	0	-34.33
Macitentan	588.273	441798-33-0	-4.383	-0.137	-0.88	-0.16	-49.529
Cidofovir (Vistide)	279.187	113852-37-2	-4.38	-0.243	-0.541	-0.478	-25.11
Benzethonium chloride	448.081	121-54-0	-4.379	-0.146	-2.19	0	-33.443
Misoprostol	382.534	59122-46-2	-4.379	-0.162	-1.625	-0.751	-31.025
Clindamycin palmitate HCl	699.853	25507-04-4	-4.376	-0.099	-1.443	-0.065	-50.788
Grazoprevir	766.903	1350514-68-9	-4.373	-0.081	-0.911	0	-47.633
Maprotiline hydrochloride	313.864	10347-81-6	-4.369	-0.208	-1.201	-0.289	-26.69
Oxaprozin	293.317	21256-18-8	-4.367	-0.199	-0.943	-0.052	-30.135
Betamipron	193.199	3440-28-6	-4.367	-0.312	-0.901	-0.178	-21.951
Nateglinide (Starlix)	317.423	105816-04-4	-4.364	-0.19	-0.753	-0.361	-25.875

Dexlansoprazole	369.361	138530-94-6	-4.361	-0.174	-0.449	-0.16	-33.504
Eliglustat (Tartrate)	404.543	491833-29-5	-4.361	-0.15	-1.198	-0.456	-39.426
Dapagliflozin	408.873	461432-26-8	-4.36	-0.156	-1.042	0	-29.818
Closantel Sodium	685.056	61438-64-0	-4.359	-0.145	-1.484	-0.297	-39.77
Flunarizine 2HCl	477.417	30484-77-6	-4.357	-0.145	-1.259	-0.008	-35.594
(+)-خ┴-Lipoic acid	206.326	1200-22-2	-4.354	-0.363	-1.097	0	-20.97
Cefotaxime sodium	477.447	64485-93-4	-4.35	-0.145	-0.853	-0.518	-45.499
L-carnitine (Levocarnitine)	161.199	541-15-1	-4.347	-0.395	-0.538	-0.16	-13.713
Erlotinib	393.436	183321-74-6	-4.346	-0.15	-1.78	-0.045	-42.161
Fingolimod (FTY720) HCl	343.932		-4.343	-0.197	-1.647	-0.144	-25.002
Fumagillin	458.544	23110-15-8	-4.342	-0.132	-1.198	-0.251	-34.296
Buflomedil HCl	343.846	35543-24-9	-4.338	-0.197	-1.196	-0.32	-31.408
Ethynodiol diacetate	384.508	297-76-7	-4.338	-0.155	-1.323	-0.02	-39.301
Mitomycin C	334.327						
Tiotropium Bromide hydrate	490.432	139404-48-1	-4.336	-0.167	-1.002	0	-32.071
Atorvastatin calcium (Lipitor)	1155.342	134523-03-8	-4.329	-0.106	-1.46	-0.48	-42.355
Dapoxetine hydrochloride (Priligy)	341.874	129938-20-1	-4.329	-0.188	-1.615	0	-30.797
Nimodipine (Nimotop)	418.44	66085-59-4	-4.328	-0.144	-0.889	-0.32	-40.344
Arecoline HBr	236.106	300-08-3	-4.327	-0.393	-0.706	-0.32	-19.162
Levothyroxine sodium	798.852	20156	-4.327	-0.18	-1.336	-0.48	-39.207
Dexamethasone Sodium Phosphate	516.405	55203-24-2	-4.321	-0.135	-1.066	-0.581	-27.945
Ramipril (Altace)	416.511	87333-19-5	-4.321	-0.144	-1.023	-0.41	-38.874
Enzastaurin (LY317615)	515.605	170364-57-5	-4.32	-0.111	-1.245	0	-49.519
Kanamycin sulfate	582.577	25389-94-0	-4.317	-0.131	-0.463	-0.261	-31.735
Clotrimazole (Canesten)	344.837	23593-75-1	-4.314	-0.173	-1.093	-0.268	-29.187
Atazanavir sulfate	802.934	229975-97-7	-4.314	-0.085	-1.461	-0.296	-49.988
Safinamide Mesylate (FCE28073)	398.449	202825-46-5	-4.314	-0.196	-1.387	-0.235	-29.171
Estradiol Benzoate	376.488	50-50-0	-4.31	-0.154	-1.063	-0.1	-38.125
Mianserin HCl	300.826	21535-47-7	-4.308	-0.215	-0.966	0	-28.091
Quinacrine 2HCl	472.879	25329	-4.308	-0.154	-1.229	-0.513	-34.098
Bepotastine Besilate	547.063	190786-44-8	-4.304	-0.159	-1.085	-0.016	-34.654
Tolnaftate	307.409	2398-96-1	-4.303	-0.196	-1.413	0	-31.541
Triclosan	289.542	3380-34-5	-4.302	-0.253	-1.077	0	-27.299
abemaciclib (LY2835219)	602.699	1231930-82-7	-4.3	-0.116	-1.575	0	-43.262
Mirtazapine (Remeron, Avanza)	265.353	85650-52-8	-4.299	-0.215	-0.92	0	-28.599

Betamethasone Dipropionate (Diprolene)	504.588	5593-20-4	-4.297	-0.119	-1.106	-0.456	-38.212
Aprepitant (MK-0869)	534.427	170729-80-3	-4.297	-0.116	-0.552	-0.258	-42.865
Moxonidine	241.677	75438-57-2	-4.297	-0.269	-0.825	-0.16	-27.559
Nelfinavir Mesylate	663.888	159989-65-8	-4.296	-0.107	-1.33	-0.306	-45.049
Dexamethasone	392.461	18296	-4.294	-0.153	-0.768	-0.152	-26.719
Propranolol HCl	295.804	318-98-9	-4.293	-0.226	-1.186	0	-27.788
Nicardipine HCl	515.986	54527-84-3	-4.292	-0.123	-0.93	-0.32	-48.649
Eugenol	164.201	97-53-0	-4.292	-0.358	-1.281	-0.168	-21.936
Sinomenine hydrochloride	365.851	6080-33-7	-4.29	-0.179	-0.894	-0.138	-31.039
Tanshinone IIA sulfonate (sodium)	396.389	69659-80-9	-4.289	-0.165	-1.166	-0.312	-28.66
Chloroquine Phosphate	515.863	50-63-5	-4.289	-0.195	-1.806	0	-34.954
Deoxycholic acid	392.572	83-44-3	-4.288	-0.153	-0.448	-0.259	-27.941
BAF312 (Siponimod)	516.595	1230487-00-9	-4.288	-0.116	-1.227	-0.063	-40.376
Pitavastatin calcium (Livalo)	880.984	147526-32-7	-4.284	-0.138	-1.118	-0.311	-36.292
Phenacetin	179.216	62-44-2	-4.282	-0.329	-0.663	-0.374	-21.439
Methenamine (Mandelamine)	140.186	100-97-0	-4.279	-0.428	0	0	-18.308
Darifenacin HBr	507.462	133099-07-7	-4.275	-0.134	-1.533	0	-41.631
Ivacaftor (VX-770)	392.491	873054-44-5	-4.273	-0.147	-0.866	-0.274	-35.836
Balsalazide disodium	437.312	150399-21-6	-4.273	-0.164	-0.368	-0.4	-22.833
Estradiol valerate	356.498	979-32-8	-4.271	-0.164	-1.248	-0.39	-27.011
Cyclobenzaprine HCl	311.848	6202-23-9	-4.27	-0.203	-1.455	0	-27.853
Verapamil HCl	491.063	152-11-4	-4.269	-0.129	-1.579	-0.331	-40.021
Busulfan (Myleran, Busulfex)	246.302	55-98-1	-4.266	-0.305	-1.082	0	-25.398
Rimonabant (SR141716)	463.787	168273-06-1	-4.263	-0.142	-1.196	-0.257	-40.154
Cobimetinib (GDC-0973, RG7420)	531.31	934660-93-2	-4.263	-0.142	-1.25	-0.16	-38.899
Finasteride	372.544	98319-26-7	-4.26	-0.158	-0.995	0	-40.603
Tiagabine hydrochloride	412.009	145821-59-6	-4.259	-0.17	-1.497	0	-33.588
4-Methylbenzylidene camphor	254.367	36861-47-9	-4.259	-0.224	-1.43	0	-25.296
L-Cysteine HCl	157.619	52-89-1	-4.258	-0.608	-0.212	-0.695	-12.908
Nisoldipine	388.414	63675-72-9	-4.257	-0.152	-1.152	-0.154	-37.225
Benidipine hydrochloride	542.023	91599-74-5	-4.257	-0.115	-1.467	0	-56.119
Amitriptyline HCl	313.864	549-18-8	-4.256	-0.203	-1.288	-0.149	-28.097
Flavoxate HCl	427.921	3717-88-2	-4.256	-0.147	-1.386	-0.332	-39.665
Perindopril Erbumine (Aceon)	441.605	107133-36-8	-4.252	-0.164	-0.993	-0.158	-33.664
Glipizide (Glucotrol)	445.535	29094-61-9	-4.248	-0.137	-0.588	-0.573	-43.156

Triamcinolone Acetonide	434.498	76-25-5	-4.248	-0.137	-0.703	-0.16	-29.088
Ampiroxicam	447.462	99464-64-9	-4.246	-0.137	-0.934	-0.01	-41.903
Artemisinin	282.332	63968-64-9	-4.246	-0.212	-1.055	0	-29.775
Vildagliptin (LAF-237)	303.399	274901-16-5	-4.245	-0.193	-0.966	-0.317	-27.468
Iproniazid	179.219	54-92-2	-4.243	-0.326	-0.695	-0.341	-25.086
Fenticonazole nitrate	518.412	73151-29-8	-4.24	-0.141	-2.07	0	-44.394
Idebenone	338.438	58186-27-9	-4.239	-0.177	-1.629	-0.275	-29.972
Buspirone hydrochloride	421.964	33386-08-2	-4.236	-0.151	-0.902	-0.319	-33.729
Efaproxiral sodium	363.383	170787-99-2	-4.235	-0.169	-0.858	-0.52	-25.817
Bosutinib (SKI-606)	530.446	380843-75-4	-4.235	-0.118	-1.183	-0.24	-46.16
Maraviroc	513.666	376348-65-1	-4.233	-0.114	-0.888	0	-42.913
Guanidine (Aminoformamidine) HCl	95.531	18264	-4.226	-1.056	0	-0.281	-8.916
Fluoxetine HCl	345.787	56296-78-7	-4.224	-0.192	-1.372	-0.16	-27.502
Halcinonide	454.959	3093-35-4	-4.221	-0.136	-0.752	-0.16	-30.036
Cilostazol	369.461	73963-72-1	-4.22	-0.156	-0.87	-0.16	-39.046
Lovastatin (Mevacor)	404.54	75330-75-5	-4.22	-0.146	-1.211	-0.445	-34.272
Venlafaxine HCl	313.863	99300-78-4	-4.219	-0.211	-0.746	-0.16	-23.908
Nizatidine	331.457	76963-41-2	-4.219	-0.201	-0.874	-0.513	-35.39
Cyclandelate	276.371	456-59-7	-4.218	-0.211	-1.234	0	-32.792
Gefitinib (ZD1839)	446.902	184475-35-2	-4.218	-0.136	-1.566	-0.001	-42.293
Phenoxybenzamine HCl	340.287	63-92-3	-4.217	-0.201	-1.487	-0.32	-30.6
Sarpogrelate hydrochloride	465.967	135159-51-2	-4.217	-0.136	-0.93	-0.344	-40.748
Pramipexole dihydrochloride monohydrate	302.264	191217-81-9	-4.216	-0.301	-0.694	-0.343	-21.01
Diltiazem HCl	450.979	33286-22-5	-4.214	-0.145	-1.475	-0.232	-38.984
Vonoprazan Fumarate (TAK-438)	461.463	1260141-27-2	-4.214	-0.176	-0.938	-0.04	-35.127
Bezafibrate	361.819	41859-67-0	-4.214	-0.169	-0.942	-0.187	-31.621
L-Thyroxine	776.87	51-48-9	-4.211	-0.175	-0.733	-0.152	-35.074
Cyproheptadine hydrochloride	323.859	41354-29-4	-4.21	-0.191	-1.277	0	-27.8
Betahistine 2HCl	209.116	5579-84-0	-4.207	-0.421	-0.454	-0.455	-15.057
Doxepin hydrochloride	315.837	1229-29-4	-4.2	-0.2	-1.23	0	-28.245
Guaifenesin (Guaiphenesin)	198.216	93-14-1	-4.2	-0.3	-0.448	-0.576	-16.566
Afatinib (BIBW2992)	485.938	439081-18-2	-4.199	-0.123	-0.982	-0.575	-40.023
Cyromazine	166.184	66215-27-8	-4.198	-0.35	-0.116	-0.465	-19.155
Roxatidine acetate HCl	384.898	93793-83-0	-4.197	-0.168	-1.303	-0.608	-34.455
Camptothecin	348.352	2114454	-4.196	-0.161	-0.506	-0.32	-33.954

Fenspiride HCl	296.792						
Diclofenac Sodium	318.13	15307-79-6	-4.194	-0.221	-1.208	-0.142	-29.431
Paroxetine HCl	365.826	78246-49-8	-4.192	-0.175	-1.129	-0.416	-29.66
Methylbenactyzine Bromide	422.356	3166-62-9	-4.191	-0.168	-1.417	0	-30.617
Diethylstilbestrol	268.35	56-53-1	-4.189	-0.209	-1.167	-0.32	-26.445
Magnolol	266.334	528-43-8	-4.185	-0.209	-1.188	-0.293	-27.629
Phenylbutazone (Butazolidin, Butatron)	308.374	50-33-9	-4.184	-0.182	-0.815	-0.138	-32.462
Taurine	125.147	107-35-7	-4.182	-0.597	-0.347	0	-11.813
Betapar (Meprednisone)	372.455	1247-42-3	-4.18	-0.155	-0.554	-0.004	-31.119
Loxapine Succinate	445.896	27833-64-3	-4.179	-0.182	-1.254	0	-30.875
Temocapril HCl	513.07	110221-44-8	-4.176	-0.131	-0.743	-0.152	-42.299
Levobetaxolol HCl	343.889	116209-55-3, 932	-4.17	-0.19	-1.742	-0.14	-30.64
Diiodohydroxyquinoline	396.951	83-73-8	-4.167	-0.321	-1.222	-0.32	-26.11
Nicaraven	284.313	79455-30-4	-4.167	-0.198	-1.09	-0.001	-32.513
Rasagiline mesylate	267.344	161735-79-1	-4.166	-0.32	-1.129	-0.195	-22.464
Mosapride	421.893	112885-41-3	-4.166	-0.144	-1.438	-0.141	-39.07
10-Hydroxycamptothecin	364.351	19685-09-7	-4.163	-0.154	-0.428	-0.068	-34.009
Prochlorperazine dimaleate salt	606.087	30718	-4.162	-0.166	-1.381	0	-37.43
Ticlopidine hydrochloride	300.247	53885-35-1	-4.161	-0.245	-1.316	0	-30.318
Methocarbamol (Robaxin)	241.241	532-03-6	-4.16	-0.245	-0.353	-0.435	-25.259
Ropinirole HCl	296.836	91374-20-8	-4.156	-0.219	-0.905	-0.283	-28.045
Verteporfin (Visudyne)	718.794	129497-78-5	-4.156	-0.078	-0.605	0	-42.308
Procarbazine hydrochloride (Matulane)	257.76	366-70-1	-4.156	-0.26	-0.848	-0.567	-26.214
diperodon hydrochloride	433.928	537-12-2	-4.154	-0.143	-1.081	-0.476	-41.741
Anastrozole	293.366	120511-73-1	-4.15	-0.189	-0.529	-0.048	-31.844
Amitraz	293.406	33089-61-1	-4.149	-0.189	-1.523	0	-34.821
Crizotinib (PF-02341066)	450.337	877399-52-5, 877	-4.149	-0.138	-1.086	-0.272	-41.454
Alfacalcidol	400.637	41294-56-8	-4.148	-0.143	-1.509	-0.277	-22.053
Ritodrine hydrochloride (Yutopar)	323.814	23239-51-2	-4.146	-0.197	-1.103	-0.92	-26.082
Cilnidipine	492.52	132203-70-4	-4.145	-0.115	-1.018	-0.204	-44.465
Bronopol	199.988	52-51-7	-4.145	-0.461	-0.24	-0.145	-12.354
Chlorprothixene	315.86	113-59-7	-4.144	-0.197	-1.511	0	-31.553
Boceprevir	519.677	394730-60-0	-4.144	-0.112	-1.08	-0.31	-40.573
Ifenprodil Tartrate	800.976	23210-58-4	-4.138	-0.172	-1.093	-0.292	-30.903
Bendamustine HCL	394.724	3543-75-7	-4.137	-0.18	-0.602	-0.098	-30.621

Acetylleucine	173.21	99-15-0	-4.134	-0.345	-0.296	-0.159	-17.737
Rilpivirine	366.419	500287-72-9	-4.128	-0.147	-0.957	-0.121	-39.449
Tolterodine tartrate (Detrol LA)	475.574	124937-52-6	-4.128	-0.172	-1.195	-0.16	-29.993
Sunitinib Malate	532.561	341031-54-7	-4.127	-0.142	-1.05	-0.16	-40.032
Brompheniramine hydrogen maleate	435.312	980-71-2	-4.125	-0.217	-1.409	-0.165	-29.449
Itopride hydrochloride	394.892	122892-31-3	-4.124	-0.159	-1.34	-0.32	-38.312
Isavuconazole	437.465	241479-67-4	-4.124	-0.133	-1.315	0	-39.802
Trihexyphenidyl hydrochloride	337.927	52-49-3	-4.12	-0.187	-1.143	-0.16	-26.55
Metaxalone	221.252	1665-48-1	-4.118	-0.257	-0.732	-0.342	-24.636
Bimatoprost	415.566	155206-00-1	-4.117	-0.137	-0.859	-0.418	-37.824
Acitretin	326.429	55079-83-9	-4.113	-0.171	-1.223	0	-27.178
Tilorone dihydrochloride	483.471	27591-69-1	-4.106	-0.137	-2.002	-0.224	-38.279
Betaine	117.146	107-43-7	-4.105	-0.513	-0.265	-0.152	-10.705
Dibucaine (Cinchocaine) HCl	379.924	22616	-4.105	-0.164	-1.485	-0.261	-37.526
Netupitant	578.592	290297-26-6	-4.101	-0.1	-0.959	0	-48.219
Mycophenolic acid	320.337	24280-93-1	-4.1	-0.178	-0.48	-0.184	-29.207
Bromhexine HCl	412.591	611-75-6	-4.1	-0.228	-1.258	-0.215	-27.315
Naltrexone HCl	377.862	16676-29-2	-4.099	-0.164	-0.787	0	-27.074
Choline Chloride	139.624	67-48-1	-4.092	-0.585	-0.366	-0.475	-9.391
Plerixafor	502.782	110078-46-1	-4.091	-0.114	-1.539	-0.16	-46.434
Rupatadine Fumarate	532.03	182349-12-8	-4.089	-0.136	-1.212	0	-38.386
Lomustine (CeeNU)	233.695	13010-47-4	-4.088	-0.273	-0.855	-0.32	-25.613
Gadodiamide Hydrate	591.672	122795-43-1	-4.086	-0.141	-0.738	-0.64	-31.002
Valpromide	143.227	2430-27-5	-4.083	-0.408	-0.848	-0.336	-16.293
Eplerenone	414.491	107724-20-9	-4.083	-0.136	-1.048	0	-42.506
Catharanthine	336.427	2468-21-5	-4.079	-0.163	-1.444	0	-31.043
Thiotepa (Thioplex)	189.218	52-24-4	-4.078	-0.371	-0.803	0	-25.699
Promethazine HCl	320.88	58-33-3	-4.077	-0.204	-0.848	-0.056	-30.864
Mepivacaine HCl	282.809	1722-62-9	-4.076	-0.226	-1.133	-0.015	-26.721
Amlodipine (Norvasc)	408.876	88150-42-9	-4.074	-0.146	-1.057	-0.468	-38.522
Empagliflozin (BI10773)	450.909	864070-44-0	-4.073	-0.131	-0.733	0	-27.394
Solifenacin succinate	480.553	242478-38-2	-4.071	-0.151	-1.157	0	-36.994
DL-Carnitine hydrochloride	197.66	461-05-2	-4.06	-0.369	-0.164	-0.082	-14.244
Propafenone HCl	341.444	34183-22-7	-4.059	-0.162	-1.257	-0.016	-37.248
Rocuronium bromide	609.678	119302-91-9	-4.058	-0.107	-1.829	-0.002	-34.415

Efavirenz	315.675	154598-52-4		-4.056	-0.193	-0.702	-0.32	-31.654
Ethylparaben	166.174	120-47-8		-4.055	-0.338	-0.54	-0.16	-21.288
Clopidogrel (Plavix)	419.9	120202-66-6		-4.055	-0.193	-1.147	-0.153	-32.711
cis-Aconitic acid	174.108	585-84-2		-4.053	-0.338	-0.088	-0.07	-14.461
Climbazole	292.761	38083-17-9		-4.048	-0.202	-0.537	-0.412	-28.143
Ipriflavone	280.318	35212-22-7		-4.047	-0.193	-1.118	0	-33.208
S-Ruxolitinib	306.365	941685-37-6		-4.045	-0.176	-1.411	0	-34.444
Phenytoin sodium (Dilantin)	274.25	630-93-3		-4.045	-0.213	-0.429	-0.388	-28.949
Atropine sulfate monohydrate	694.833	5908-99-6		-4.045	-0.193	-0.867	-0.28	-26.412
Rifampin	822.94	13292-46-1		-4.042	-0.069	-0.976	-0.101	-43.246
Etoposide (VP-16)	588.557							
Adapalene	412.52	106685-40-9		-4.033	-0.13	-1.217	0	-35.596
Ranitidine Hydrochloride	350.865	66357-59-3		-4.033	-0.192	-1.42	-0.16	-32.436
Brucine sulfate salt hydrate	510.557							
-3.672	243.934		-0.013	-0.009	0.018	-0.12	0.008	-0.113
Vardenafil Hydrochloride Trihydrate (Vivanza	579.11	224785-90-4		-4.031	-0.119	-0.672	0	-46.3
Gabexate mesylate	417.477	56974-61-9		-4.029	-0.175	-1.229	-0.225	-29.609
Cloxacillin sodium	475.878	7081-44-9		-4.028	-0.139	-0.827	0	-34.512
Etravirine (TMC125)	435.277	269055-15-4		-4.025	-0.144	-1.005	0	-42.716
Picropodophyllin (PPP)	414.405	477-47-4		-4.021	-0.134	-1.113	-0.087	-36.274
Eprazinone 2HCl	453.445	10402-53-6		-4.016	-0.143	-1.332	0	-38.956
Budesonide	430.534	51333-22-3		-4.014	-0.129	-0.728	-0.152	-27.93
Methacholine chloride	195.687	62-51-1		-4.013	-0.365	-0.495	-0.09	-17.527
Gallamine triethiodide (Flaxedil)	891.529	65-29-2		-4.009	-0.111	-1.59	0	-39.603
Cefuroxime sodium	446.367	56238-63-2		-3.999	-0.138	-0.744	-0.07	-36.402
Malotilate	288.383	59937-28-9		-3.996	-0.222	-0.888	-0.396	-31.638
Azlocillin sodium salt	484.481	37091-65-9		-3.995	-0.125	-0.98	0	-46.908
Tolperisone HCl	281.821	3644-61-9		-3.995	-0.222	-1.19	0	-25.085
Benztropine mesylate	403.535	132-17-2		-3.993	-0.174	-1.619	0	-30.718
Diphenidol HCl	345.906	3254-89-5		-3.985	-0.173	-1.4	-0.162	-28.569
Gestodene	310.43	60282-87-3		-3.985	-0.173	-1.002	0	-28.223
Betaxolol (Betoptic)	307.428	659-18-7		-3.982	-0.181	-1.93	0	-35.084
Imatinib Mesylate (STI571)	589.708	220127-57-1		-3.982	-0.108	-1.443	-0.167	-48.027
Sulfabenzamide	276.311	127-71-9		-3.98	-0.209	-0.403	-0.148	-29.43
Colchicine	399.437	64-86-8		-3.974	-0.137	-1.397	-0.014	-39.556

Orphenadrine citrate (Norflex)	461.505	4682-36-4	-3.972	-0.199	-1.421	-0.198	-25.203
Anamorelin	546.704	249921-19-5	-3.971	-0.099	-0.49	-0.34	-44.523
Artesunate	384.421	88495-63-0	-3.97	-0.147	-0.389	-0.187	-29.697
Nitrendipine	360.361	39562-70-4	-3.97	-0.153	-0.649	-0.32	-38.457
Vismodegib (GDC-0449)	421.297	879085-55-9	-3.969	-0.147	-0.896	0	-40.025
Sunitinib	398.474	557795-19-4	-3.967	-0.137	-0.93	-0.16	-39.825
Rosuvastatin Calcium	1001.137	147098-20-2	-3.966	-0.12	-0.393	-0.32	-36.675
Amorolfine Hydrochloride	353.97	78613-38-4	-3.966	-0.172	-1.159	-0.32	-31.38
Imperatorin	270.28	482-44-0	-3.965	-0.198	-0.956	0	-29.688
Mifepristone (Mifeprex)	429.594	84371-65-3	-3.965	-0.124	-1.263	0	-37.727
Gamma-Oryzanol	602.886	11042-64-1	-3.962	-0.09	-1.287	-0.08	-43.473
Amprenavir (Agenerase)	505.627	161814-49-9	-3.959	-0.113	-1.192	0	-41.237
Captopril (Capoten)	217.285	62571-86-2	-3.956	-0.283	-0.602	-0.152	-24.751
guanethidine sulfate	494.695	21952	-3.954	-0.282	-0.56	-0.307	-19.694
Reboxetine mesylate	409.496	98769-84-7	-3.953	-0.172	-1.41	-0.32	-27.907
Levodropropizine	236.31	99291-25-5	-3.95	-0.232	-0.764	0	-21.279
Azathramycin	734.958	76801-85-9	-3.947	-0.077	-0.793	-0.272	-38.48
Hederagenin	472.7	465-99-6	-3.945	-0.116	-1.236	-0.13	-28.158
Carmofur	257.261	61422-45-5	-3.94	-0.219	-0.81	-0.16	-25.877
Chlormezanone (Trancopal)	273.736	80-77-3	-3.937	-0.232	-0.613	0	-27.059
Sitafloxacin hydrate	873.674	163253-35-8, 163	-3.934	-0.14	-1.036	0	-35.312
Tenofovir Disoproxil Fumarate	635.515	202138-50-9	-3.932	-0.112	-0.665	-0.212	-49.653
Clomipramine hydrochloride (Anafranil)	351.313	17321-77-6	-3.93	-0.179	-0.918	0	-32.731
Etretinate	354.483	54350-48-0	-3.929	-0.151	-1.887	0	-36.52
Molsidomine	242.232	25717-80-0	-3.929	-0.231	-0.613	-0.133	-25.349
Ceritinib (LDK378)	558.135	1032900-25-6	-3.928	-0.103	-1.477	0	-49.558
Pimavanserin	1005.196	706782-28-7	-3.924	-0.127	-1.305	0	-40.435
Chlorpheniramine Maleate	390.861	113-92-8	-3.917	-0.206	-1.351	-0.014	-28.821
Cinnarizine	368.514	298-57-7	-3.909	-0.14	-1.324	-0.031	-34.231
Pentoxyverine Citrate	437.47	23142-01-0	-3.907	-0.156	-1.001	-0.16	-30.515
Cisapride hydrate	483.961	260779-88-2	-3.901	-0.122	-1.456	-0.16	-34.51
Ketotifen fumarate (Zaditor)	425.497	34580-14-8	-3.9	-0.177	-0.884	0	-29.725
Fluphenazine (dihydrochloride)	510.443	146-56-5	-3.894	-0.13	-0.841	-0.608	-35.382
Deflazacort (Calcort)	441.517	14484-47-0	-3.891	-0.122	-0.862	-0.16	-33.559
Torcetrapib (CP-529414)	600.473	262352-17-0	-3.88	-0.095	-0.463	-0.207	-38.454

Nicorandil (Ikorel)	211.175	65141-46-0	-3.879	-0.259	-0.784	-0.419	-25.529
Terbinafine	291.43	91161-71-6	-3.879	-0.176	-1.581	-0.014	-31.184
Vitamin E	430.706	21590	-3.872	-0.125	-1.713	-0.229	-30.553
Binimetinib (MEK162, ARRY-162, ARRY-438162)	441.227	606143-89-9	-3.871	-0.143	-0.567	-0.16	-34.476
Quetiapine fumarate (Seroquel)	883.086	111974-72-2	-3.87	-0.143	-0.855	-0.263	-29.422
Benzydamine Hydrochloride	345.866	132-69-4	-3.868	-0.168	-1.294	0	-33.47
Milnacipran HCl	282.809	101152-94-7	-3.867	-0.215	-0.904	-0.27	-24.074
trimipramine maleate	410.506	521-78-8	-3.866	-0.176	-0.8	0	-30.581
Valnemulin HCl	601.281	133868-46-9	-3.862	-0.099	-1.036	-0.455	-38.475
Topotecan HCl	457.907	119413-54-6	-3.86	-0.125	-0.669	-0.16	-35.243
Dextrose (D-glucose)	180.156	50-99-7	-3.858	-0.322	-0.5	0	-19.984
clofoctol	365.337	37693-01-9	-3.854	-0.161	-0.961	-0.16	-31.645
IMipraMine hydrochloride	316.868	113-52-0	-3.853	-0.183	-0.877	0	-30.125
Docusate Sodium	444.558	577-11-7	-3.846	-0.137	-1.837	-0.241	-30.216
Aspartame	294.303	22839-47-0	-3.845	-0.183	-0.389	-0.919	-27.757
Alcaftadine	307.39	147084-10-4	-3.843	-0.167	-0.429	-0.118	-30.994
Terbinafine hydrochloride (Lamisil)	327.891	78628-80-5	-3.842	-0.175	-1.615	0	-30.929
Dehydroandrographolide	332.434	134418-28-3	-3.837	-0.16	-1.09	0	-25.56
Simvastatin (Zocor)	418.566	79902-63-9	-3.833	-0.128	-0.96	-0.32	-31.484
Glucosamine sulfate	277.25	29031-19-4	-3.831	-0.319	-0.591	-0.431	-18.727
Monobenzone (Benoquin)	200.233	103-16-2	-3.83	-0.255	-0.88	-0.16	-22.954
Citalopram HBr	405.304	59729-32-7	-3.829	-0.16	-0.982	-0.192	-32.613
arbinoxaMine Maleate	406.86	3505-38-2	-3.824	-0.191	-1.128	-0.192	-29.88
Lidocaine (Alphacaine)	234.337	137-58-6	-3.823	-0.225	-1.19	-0.08	-26.14
Escitalopram Oxalate	414.427	219861-08-2	-3.822	-0.159	-0.975	-0.194	-32.601
All-trans Retinoic Acid (Tretinoin)	300.435	302-79-4	-3.819	-0.174	-0.965	0	-27.381
Chenodeoxycholic acid	392.572	474-25-9	-3.816	-0.136	-0.921	-0.085	-24.459
Oxybutynin chloride	393.947	1508-65-2	-3.813	-0.147	-1.197	0	-29.101
Capsaicin	305.412	404-86-4	-3.812	-0.173	-1.48	-0.124	-30.851
Benserazide HCl	293.704	14919-77-8	-3.808	-0.212	-0.899	-0.32	-26.061
Artemether	298.375	71963-77-4	-3.808	-0.181	-0.823	0	-26.137
Etonogestrel	324.457	54048-10-1	-3.807	-0.159	-0.922	0	-28.321
Clarithromycin (Biaxin, Klacid)	747.953	81103-11-9	-3.804	-0.073	-0.542	-0.288	-28.493
Isotretinoin	300.435	4759-48-2	-3.799	-0.173	-1.002	0	-26.185
Fesoterodine Fumarate	527.649	286930-03-8	-3.798	-0.127	-0.908	-0.321	-34.81

Calcipotriene	412.605	112965-21-6	-3.798	-0.127	-1.248	-0.456	-26.521
Pyrilamine maleate	401.456	59-33-6	-3.792	-0.181	-1.224	-0.163	-31.301
Doxapram HCl	432.983	7081-53-0	-3.788	-0.135	-0.757	-0.317	-33.2
Paeoniflorin	480.462	23180-57-6	-3.788	-0.111	-0.8	0	-34.769
Dronedarone HCl (Multaq)	593.217	141625-93-6	-3.786	-0.097	-1.791	0	-43.512
Tiagabine	375.548	115103-54-3	-3.772	-0.151	-1.047	-0.162	-34.247
Butylparaben	194.227	94-26-8	-3.771	-0.269	-0.893	-0.32	-22.839
Acetylcholine iodide	273.112	2260-50-6	-3.77	-0.377	-0.454	-0.141	-16.532
procyclidine hydrochloride	323.901	1508-76-5	-3.768	-0.179	-0.942	-0.16	-23.27
Benzyl isothiocyanate	149.213	622-78-6	-3.768	-0.377	-0.724	0	-22.78
Piperacillin Sodium	539.537	59703-84-3	-3.768	-0.105	-0.526	-0.008	-46.397
Osthole (Osthol)	244.286	484-12-8	-3.767	-0.209	-0.999	-0.05	-31.955
HMN-214	424.47	173529-46-9	-3.766	-0.126	-0.982	0	-38.976
Neratinib (HKI-272)	557.043	698387-09-6	-3.765	-0.094	-0.807	-0.297	-44.59
Pheniramine Maleate	356.416	132-20-7	-3.762	-0.209	-1.402	-0.024	-26.327
Sildenafil	474.576	139755-83-2	-3.761	-0.114	-0.734	0	-41.807
Hydroxyzine 2HCl	447.826	2192-20-3	-3.759	-0.145	-1.357	-0.296	-27.298
disopyramide phosphate	437.47	22059-60-5	-3.756	-0.15	-0.959	-0.16	-30.397
VX-745	436.262	209410-46-8	-3.756	-0.134	-1.232	-0.059	-39.56
Lidocaine hydrochloride	270.798	73-78-9	-3.751	-0.221	-1.097	-0.16	-25.701
Triacetin	218.204	102-76-1	-3.749	-0.25	-0.621	-0.608	-28.725
Nedaplatin	269.113	95734-82-0	-3.735	-0.623	-0.28	-0.32	-10.819
Fluvoxamine maleate	434.407	61718-82-9	-3.735	-0.17	-0.54	-0.608	-24.129
Azelastine hydrochloride (Astelin)	418.359	79307-93-0	-3.725	-0.138	-1.078	-0.015	-36.744
Erlotinib(OSI-744)	429.897	183319-69-9	-3.722	-0.128	-0.882	-0.16	-40.479
Piperonyl butoxide	338.438	18693	-3.722	-0.155	-1.395	-0.32	-34.926
Tripelennamine HCl	291.819	154-69-8	-3.721	-0.196	-1.224	0	-28.603
Alprostadil(Caverject)	354.481	745-65-3	-3.711	-0.148	-1.679	-0.16	-26.691
Imatinib (STI571)	493.603	152459-95-5	-3.709	-0.1	-1.373	0	-54.656
Nicergoline	484.386	27848-84-6	-3.708	-0.12	-0.772	-0.32	-40.741
Bambuterol HCl	403.901	81732-46-9	-3.707	-0.143	-0.586	-0.44	-34.144
Isradipine (Dynacirc)	371.387	75695-93-1	-3.707	-0.137	-0.609	-0.054	-38.466
Xylose	150.13	25990-60-7	-3.707	-0.371	-0.513	0	-16.563
Bosentan Hydrate	569.629	157212-55-0	-3.702	-0.095	-0.812	0	-43.476
Felodipine (Plendil)	384.254	72509-76-3	-3.7	-0.148	-0.717	-0.306	-38.788

Carbenoxolone disodiuM	614.72	7421-40-1	-3.697	-0.09	-0.87	-0.214	-37.947
Sodium Gluconate	218.137	527-07-1	-3.695	-0.284	-0.494	0	-18.964
Amisulpride	369.479	71675-85-9	-3.68	-0.147	-0.508	0	-35.804
Arbidol HCl	513.875	131707-23-8	-3.678	-0.127	-1.658	0	-44.133
Pantoprazole sodium	405.352	138786-67-1	-3.674	-0.141	-1.043	-0.139	-28.556
Arteether	312.401	75887-54-6	-3.672	-0.167	-0.844	0	-27.427
Acetylcysteine	163.195	616-91-1	-3.671	-0.367	-0.233	-0.118	-19.996
Quinestrol	364.52	152-43-2	-3.664	-0.136	-1.162	0	-39.061
Eperisone hydrochloride	295.847	56839-43-1	-3.661	-0.193	-0.738	0	-27.085
Bupivacaine hydrochloride (Marcain)	324.889	18010-40-7	-3.66	-0.174	-1.379	0	-30.116
Diphenhydramine HCl (Benadryl)	291.816	147-24-0	-3.659	-0.193	-1.544	0	-27.309
Betamethasone valerate (Betnovate)	476.577	2152-44-5	-3.657	-0.108	-1.241	-0.127	-36.247
Sinomenine (Cucoline)	329.39	115-53-7	-3.653	-0.152	-0.853	0	-27.229
Divalproex sodium	310.405	76584-70-8	-3.652	-0.365	-0.934	-0.23	-14.776
camylofine chlorhydrate	393.391	54-30-8	-3.636	-0.158	-1.024	-0.16	-30.721
Azelnidipine	582.646	123524-52-7	-3.634	-0.085	-0.318	-0.16	-48.314
Rivastigmine tartrate (Exelon)	400.424	129101-54-8	-3.63	-0.202	-1.171	0	-28.167
Pramiracetam	269.383	68497-62-1	-3.621	-0.191	-0.637	-0.32	-26.151
Testosterone Enanthate	400.594	315-37-7	-3.615	-0.125	-1.057	-0.085	-36.101
Rivastigmine	250.337	123441-03-2	-3.612	-0.201	-1.189	0	-28.165
Cyproterone acetate	416.938	427-51-0	-3.611	-0.125	-0.864	0	-33.851
Fosinopril sodium (Monopril)	585.644	88889-14-9	-3.606	-0.092	-0.556	-0.312	-43.701
Diethylcarbamazine (citrate)	391.417	1642-54-2	-3.605	-0.258	-0.595	0	-20.724
Prilocaine	220.311	721-50-6	-3.603	-0.225	-1.038	-0.186	-26.034
Desogestrel	310.473	54024-22-5	-3.598	-0.156	-1.015	0	-25.519
Propiverine hydrochloride	403.942	54556-98-8	-3.591	-0.133	-1.361	0	-36.701
D-Mannitol (Osmitrol)	182.172	69-65-8	-3.586	-0.299	-0.318	0	-14.195
Dimethyl Fumarate	144.125	624-49-7	-3.585	-0.359	-0.086	-0.4	-21.044
Danoprevir (ITMN-191)	731.831	850876-88-9, 916	-3.583	-0.07	-1.42	-0.317	-56.142
Butamben	193.242	94-25-7	-3.582	-0.256	-0.801	-0.349	-22.726
Scopolamine hydrobromide	384.265	114-49-8	-3.582	-0.163	-0.277	-0.344	-24.656
Miltefosine (Hexadecylphosphocholine)	407.568	58066-85-6	-3.581	-0.133	-1.824	-0.16	-34.361
Chloroprocaine HCl	307.216	3858-89-7	-3.581	-0.199	-1.101	-0.083	-28.442
Vitamin D3 (Cholecalciferol)	384.638	67-97-0	-3.579	-0.128	-1.21	-0.16	-23.266
Bedaquiline fumarate	671.577	845533-86-0	-3.577	-0.097	-1.413	0	-42.915

Retapamulin	517.763	224452-66-8	-3.562	-0.099	-0.974	-0.251	-31.841
Azithromycin	748.984	83905-01-5	-3.56	-0.068	-0.639	-0.2	-40.931
Chloroambucil	304.212	305-03-3	-3.558	-0.187	-0.733	0	-26.404
Diclazuril	407.638	101831-37-2	-3.557	-0.137	-0.603	-0.078	-38.862
Tamoxifen Citrate (Nolvadex)	563.638	54965-24-1	-3.553	-0.127	-0.968	-0.174	-33.749
Tamoxifen	563.638	ICI 46474	-3.553	-0.127	-0.968	-0.174	-33.749
Allylthiourea	116.185	109-57-9	-3.548	-0.507	-0.206	-0.456	-14.156
Adefovir Dipivoxil (Preveon, Hepsera)	501.471	142340-99-6	-3.543	-0.104	-0.53	-0.373	-40.541
Atenolol	266.336	29122-68-7	-3.522	-0.185	-1.105	-0.785	-26.479
Levobupivacaine HCl	324.889	27262-48-2	-3.518	-0.168	-1.21	0	-29.444
Meclizine dihydrochloride	463.87	1104-22-9	-3.499	-0.125	-1.148	0	-35.595
Afatinib (BIBW2992) Dimaleate	718.083	850140-73-7	-3.498	-0.103	-0.474	-0.333	-39.843
Fusidate Sodium	538.691	751-94-0	-3.496	-0.094	-1.495	-0.304	-29.038
Ursolic acid (Malol)	456.7	77-52-1	-3.495	-0.106	-0.993	0	-40.686
Azithromycin Dihydrate	785.015	117772-70-0	-3.494	-0.067	-0.665	0	-36.636
Bisoprolol fumarate	441.515	104344-23-2	-3.486	-0.152	-0.276	-0.831	-24.252
Apixaban	459.497	503612-47-3	-3.47	-0.102	-0.888	0	-38.439
Sildenafil Mesylate	570.682	1308285-21-3	-3.47	-0.105	-0.371	0	-44.043
Mesna (Uromitexan, Mesnex)	164.179	19767-45-4	-3.468	-0.495	-0.047	-0.48	-8.579
Erythromycin Cyclocarbonate	759.921	55224-05-0	-3.468	-0.065	-0.848	-0.043	-39.999
Benactyzine hydrochloride	363.878	57-37-4	-3.466	-0.144	-0.65	0	-29.21
Lappaconitine	584.7	32854-75-4	-3.455	-0.082	-0.708	-0.142	-36.606
Ginkgolide C	440.398	15291-76-6	-3.452	-0.111	-0.241	0	-25.577
Rilmenidine Phosphate	278.242	85409-38-7	-3.449	-0.265	-0.858	-0.16	-20.104
Cyclophosphamide monohydrate	279.101	6055-19-2	-3.447	-0.246	-0.227	-0.111	-23.36
pimozide	461.546	2062-78-4	-3.442	-0.101	-0.638	0	-43.346
Ciclesonide	540.688	126544-47-6	-3.428	-0.088	-0.947	-0.134	-39.348
Clevidipine Butyrate	456.316	167221-71-8	-3.423	-0.114	-0.577	-0.216	-45.76
Sodium butyrate	110.087	156-54-7	-3.414	-0.569	-0.109	-0.16	-6.159
Acebutolol HCl	372.887	34381-68-5	-3.406	-0.142	-0.854	-0.127	-35.231
Procainamide HCl	271.786	614-39-1	-3.404	-0.2	-0.759	-0.228	-26.347
Neticonazole Hydrochloride	338.895	130773-02-3	-3.392	-0.162	-1.291	0	-34.614
Tipifarnib (Zarnestra)	489.396	192185-72-1	-3.392	-0.1	-1.121	-0.159	-43.702
Cetirizine Dihydrochloride	461.81	83881-52-1	-3.39	-0.126	-0.522	0	-33.45
Cyclizine 2HCl	339.303	5897-18-7	-3.381	-0.169	-1.011	0	-27.832

Domiphen Bromide	414.463	538-71-6	-3.377	-0.141	-1.777	0	-32.654
Roflumilast (Daxas)	403.207	162401-32-3	-3.377	-0.13	-0.719	0	-38.443
Oxacillin sodium monohydrate	441.433	7240-38-2	-3.362	-0.12	-0.667	0	-40.176
Daminozide	160.171	1596-84-5	-3.357	-0.305	-0.081	-0.338	-17.652
Mozavaptan	427.538	137975-06-5	-3.356	-0.105	-0.92	-0.21	-32.526
proadifen hydrochloride	389.959	62-68-0	-3.353	-0.129	-1.243	-0.005	-36.278
Otilonium Bromide	563.567	26095-59-0	-3.352	-0.096	-1.206	-0.095	-38.036
Perifosine (KRX-0401)	461.658	157716-52-4	-3.343	-0.108	-1.673	-0.16	-32.056
Vitamin A Acetate	328.488	127-47-9	-3.333	-0.139	-1.128	0	-35.917
Ebastine	469.658	90729-43-4	-3.3	-0.094	-1.436	0	-49.781
Tiopronin (Thiola)	163.195	19392	-3.297	-0.33	-0.412	-0.152	-18.164
Procaine (Novocaine) HCl	272.771	18756	-3.289	-0.193	-1.106	-0.163	-26.839
Fluticasone propionate (Flonase, Veramyst)	500.571	80474-14-2	-3.288	-0.097	-0.66	-0.16	-30.908
Procaine	236.31	59-46-1	-3.281	-0.193	-1.104	-0.16	-26.836
Ibutilide fumarate	885.225	122647-32-9	-3.275	-0.126	-0.883	-0.304	-25.276
Selexipag	496.622	475086-01-2	-3.274	-0.094	-1.177	0	-46.313
oxelaidin citrate	527.604	52432-72-1	-3.261	-0.136	-1.221	-0.014	-32.23
Clomifene citrate (Serophene)	598.083	50-41-9	-3.257	-0.112	-1.324	0	-37.351
Ambrisentan	378.421	177036-94-1	-3.252	-0.116	-0.879	0	-35.753
Ropivacaine HCl	310.862	98717-15-8	-3.252	-0.163	-0.875	0	-26.187
Meglumine	195.214	6284-40-8	-3.249	-0.25	-0.378	-0.141	-18.415
Sorbitol (Glucitol)	182.172	50-70-4	-3.248	-0.271	-0.069	-0.32	-12.056
Fumaric acid	116.072	110-17-8	-3.238	-0.405	-0.208	-0.119	-12.631
Toremifene Citrate (Fareston, Acapodene)	598.083	89778-27-8	-3.235	-0.112	-1.136	0	-34.153
Vitamin D2 (Ergocalciferol)	396.648	50-14-6	-3.23	-0.111	-0.989	-0.16	-28.744
Probucol	516.842	23288-49-5	-3.203	-0.092	-1.264	0	-39.319
Oxybuprocaine HCl	344.877	5987-82-6	-3.194	-0.145	-0.783	-0.32	-33.105
Fusidine	516.709	1859240	-3.19	-0.086	-0.704	-0.221	-33.546
Levocetirizine Dihydrochloride	461.81	130018-87-0	-3.177	-0.118	-0.294	0	-33.676
Orlistat (Alli, Xenical)	495.735	96829-58-2	-3.175	-0.091	-1.362	-0.4	-44.8
Xylitol	152.146	87-99-0	-3.175	-0.318	-0.586	0	-11.272
Halothane	197.382	151-67-7	-3.161	-0.452	-0.258	0	-12.922
Lacidipine (Lacipil, Motens)	455.543	103890-78-4	-3.16	-0.096	-1.06	0	-43.618
Sodium Nitrite	68.995	7632-00-0	-3.159	-1.053	0	0	-1.076
Oxybutynin (Ditropan)	357.486	5633-20-5	-3.144	-0.121	-0.769	-0.309	-33.081

5-Aminolevulinic acid hydrochloride	167.591	1297222	-3.116	-0.346	-0.168	-0.326	-13.509
Clemastine Fumarate	459.962	14976-57-9	-3.104	-0.129	-1.022	0	-32.692
Doxylamine Succinate	388.457	562-10-7	-3.092	-0.155	-0.517	-0.113	-27.653
Aminoguanidine (hydrochloride)	110.546	1937-19-5	-3.068	-0.614	0	-0.534	-5.956
Vitamin A Palmitate	524.86	79-81-2	-3.028	-0.08	-2.368	0	-41.624
Erythromycin	733.927	114-07-8	-3.013	-0.059	-0.704	0	-37.952
Tiamulin fumarate	609.814	55297-96-6	-3.01	-0.089	-0.574	-0.343	-33.787
Ifosfamide	261.086	3778-73-2	-3.004	-0.215	-0.399	0	-25.537
Enoxolone (Glycyrrhetin)	470.684	471-53-4	-2.998	-0.088	-0.596	0	-40.054
Vinblastine sulfate	909.053	143-67-9	-2.971	-0.05	-0.565	-0.218	-37.956
Diatrizoate sodium	635.895	737-31-5	-2.957	-0.148	-0.291	-0.317	-30.026
BIBR-1048 (Dabigatran)	627.733	211915-06-9	-2.953	-0.064	-0.765	0	-50.429
Disulfiram (Antabuse)	296.539	97-77-8	-2.944	-0.184	-0.516	0	-26.247
Vincristine Sulfate	923.036	2068-78-2	-2.925	-0.049	-0.932	-0.028	-36.2
Trometamol	121.135	77-86-1	-2.918	-0.365	-0.301	-0.057	-8.284
Mechlorethamine HCl	192.514	55-86-7	-2.913	-0.364	-0.574	0	-17.602
Pargyline hydrochloride	195.689	306-07-0	-2.902	-0.242	-0.777	0	-23.543
Cimetidine (Tagamet)	252.339	51481-61-9	-2.885	-0.17	-1.016	-0.678	-23.597
DicycloMine Hydrochloride	345.948	67-92-5	-2.883	-0.131	-0.726	0	-27.23
Esmolol HCl	331.835	81161-17-3, 8114	-2.825	-0.135	-1.223	-0.456	-28.371
Pamidronate Disodium	279.033	57248-88-1	-2.813	-0.216	-0.022	-0.033	-12.061
Sorbic acid	112.127	110-44-1	-2.801	-0.35	-0.332	-0.088	-13.235
Aminocaproic acid-┼ثرخ-٦ثذ	131.173	60-32-2	-2.794	-0.31	-0.26	-0.563	-11.88
fosfomycin tromethamine	259.194	78964-85-9	-2.762	-0.345	-0.422	0	-9.619
Succinic acid	118.088	110-15-6	-2.67	-0.334	-0.087	-0.428	-14.575
Erythritol	122.12	149-32-6	-2.648	-0.331	0	0	-6.719
Amifostine	268.269	112901-68-5	-2.572	-0.214	-0.566	-0.708	-13.597
Dyclonine hydrochloride	325.873	536-43-6	-2.537	-0.121	-1.137	-0.167	-31.111
Teprenone	330.547	6809-52-5	-2.47	-0.103	-1.072	-0.138	-31.351
Metoprolol tartrate	684.815	392-17-7	-2.321	-0.122	-1.337	-0.345	-28.313
Metformin hydrochloride (Glucophage)	165.625	1115-70-4	-2.313	-0.257	-0.154	-0.304	-19.362
Vecuronium Bromide	637.731	50700-72-6	-2.311	-0.058	-0.458	0	-23.408
2-Aminoheptane	115.217	123-82-0	-2.306	-0.288	-0.314	-0.304	-11.995
Tetracaine hydrochloride (Pontocaine)	300.824	136-47-0	-2.278	-0.12	-1.52	-0.159	-30.29
Tolbutamide	270.348	64-77-7	-2.204	-0.122	-0.903	-0.32	-25.76

Vindoline	456.531	2182-14-1	-2.14	-0.065	-0.54	0	-30.593
Gemfibrozil (Lopid)	250.333	25812-30-0	-2.095	-0.116	-1.276	0	-25.608
Pramoxine HCl	329.862	637-58-1	-2.081	-0.099	-1.315	-0.318	-30.564
Articaine HCl	320.835	23964-57-0	-1.984	-0.104	-0.635	-0.346	-28.596
Succinylcholine Chloride Dihydrate	397.336	6101-15-1	-1.978	-0.099	-0.563	-0.32	-25.503
Alverine Citrate	473.559	5560-59-8	-1.891	-0.09	-1.225	0	-31.371
Proparacaine HCl	330.85	1452000	-1.712	-0.082	-1.165	-0.083	-30.153
Vorinostat (SAHA, MK0683)	264.32	149647-78-9	-1.665	-0.088	-0.318	-0.483	-23.423
Bufexamac	223.268	2438-72-4	-1.55	-0.097	-0.495	-0.47	-27.202
L-Arginine HCl	210.662	1119-34-2	-1.466	-0.122	-0.256	-1.066	-12.566
Ethambutol HCl	277.232	1070-11-7	-1.302	-0.093	-1.189	-0.82	-19.442
Fudosteine	179.237	13189-98-5	-0.624	-0.057	-0.109	-0.668	-14.982
DL-Panthenol	205.251	16485-10-2	-0.272	-0.019	-0.237	-0.64	-13.959
D panthenol	205.251	81-13-0	-0.068	-0.005	-0.452	-0.301	-19.836
Spermine	202.34	71-44-3	1.063	0.076	-1.214	-0.499	-22.269
Azelaic acid	188.221	123-99-9	1.248	0.096	-0.441	-0.11	-19.066
Lauric Acid	200.318	143-07-7	1.484	0.106	-1.581	-0.136	-15.569
Zinc Undecylenate	431.925	557-08-4	1.684	0.13	-1.301	-0.32	-14.853
1-Hexadecanol	242.441	36653-82-4	2.381	0.14	-1.704	-0.16	-22.555
