# Supplementary figures and images for: Repurposing FDA-approved phytomedicines, natural products, antivirals and cell protectives against SARS-CoV-2 (COVID-19) RNA-dependent RNA polymerase
Source: PeerJ. 2020 Nov 30;8:e10480. doi: 10.7717/peerj.10480 (PMC7713599; doi:10.7717/peerj.10480)

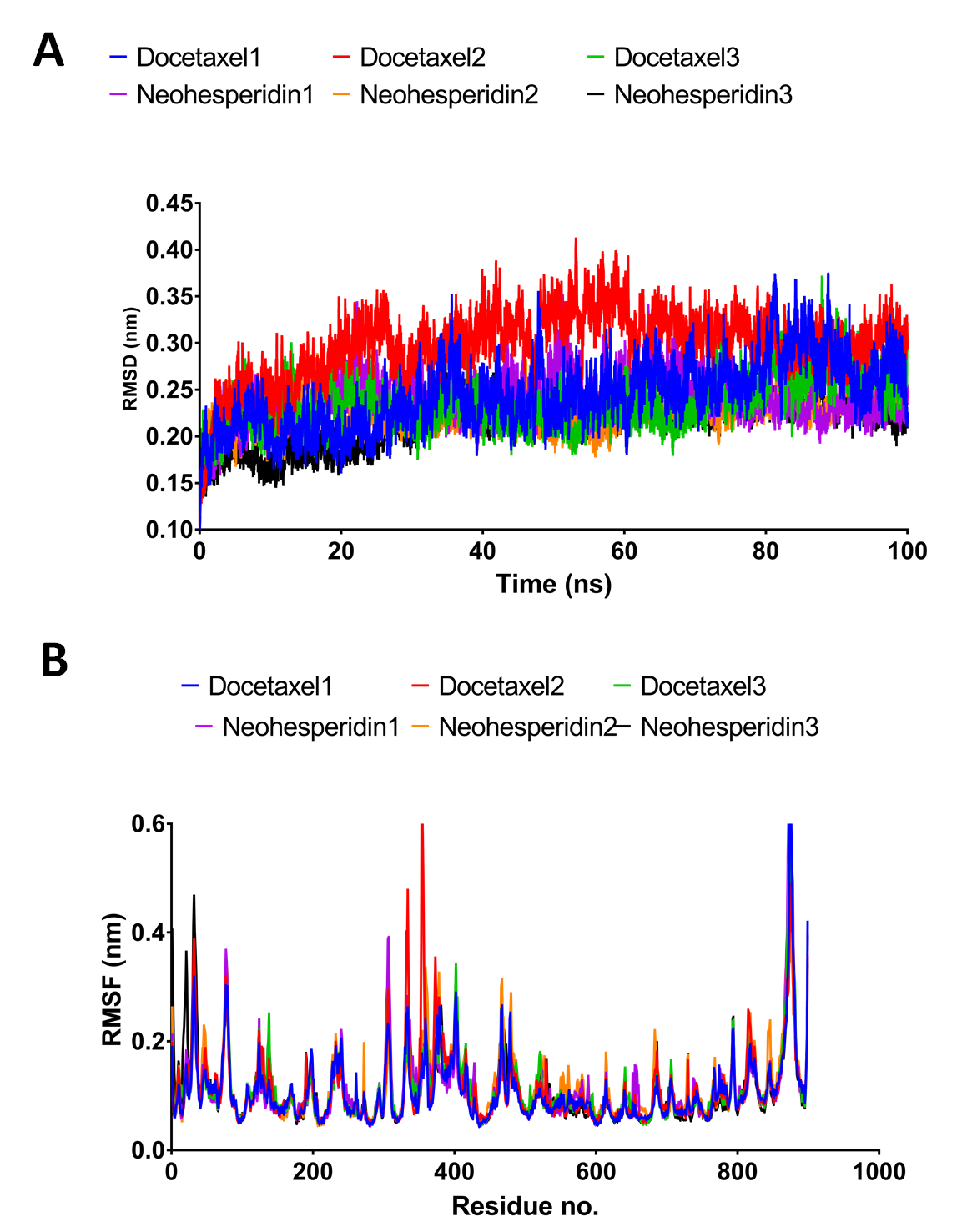

Supplement: Supplemental Information 2 — The RMSD during 100 ns simulation of the top 2 compounds after MD simulation for 20 ns, docetaxel and neohesperidin. (A) RMSD of docetaxel and neohesperidin (B) The per-residue RMSF of RdRP bound with docetaxel and neohesperidin. The average from the three experiments is displayed in Fig. 4. [file peerj-08-10480-s002.png]

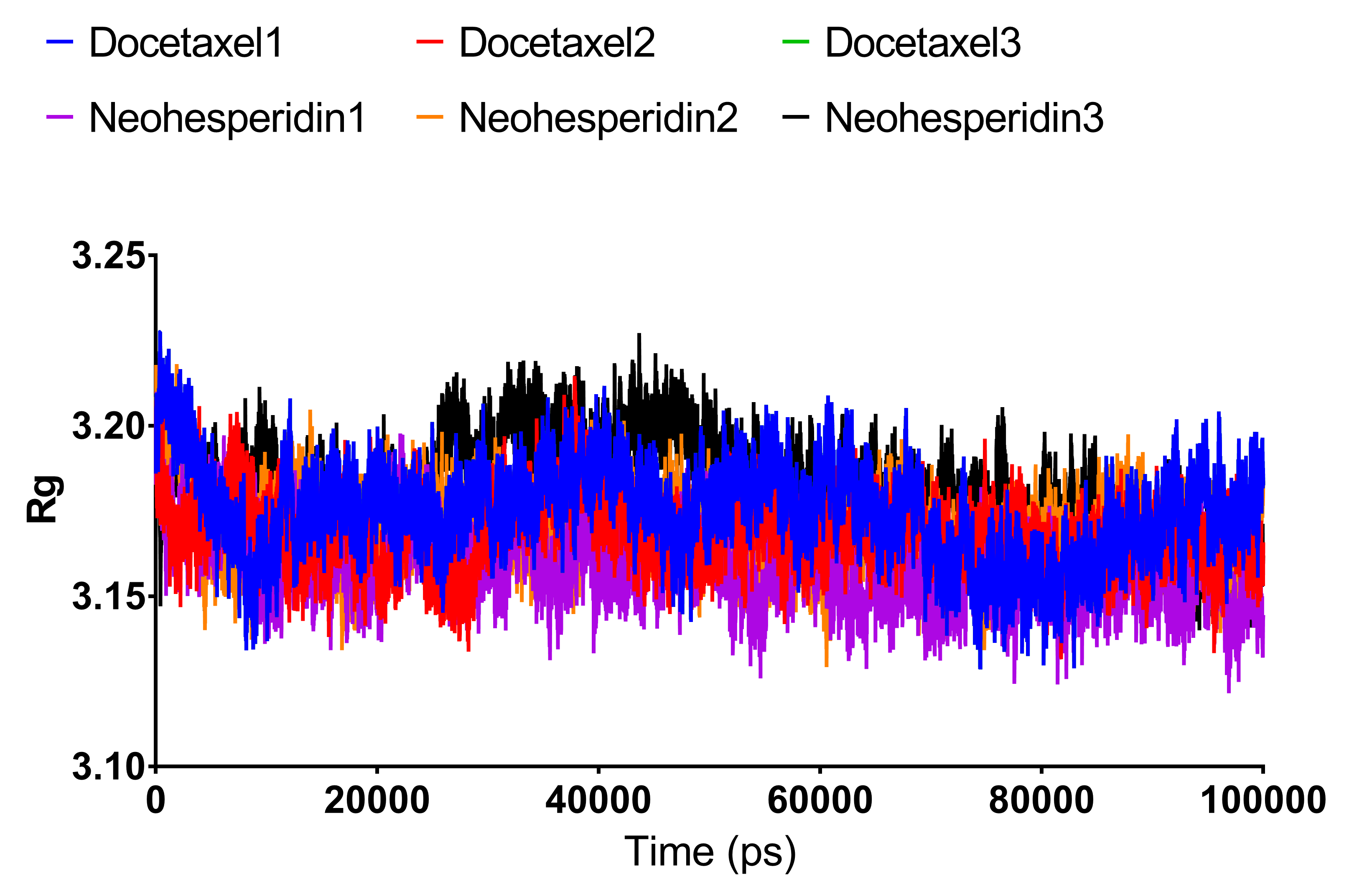

Supplement: Supplemental Information 3 — The average from the three experiments is displayed in Fig. 5. [file peerj-08-10480-s003.png]

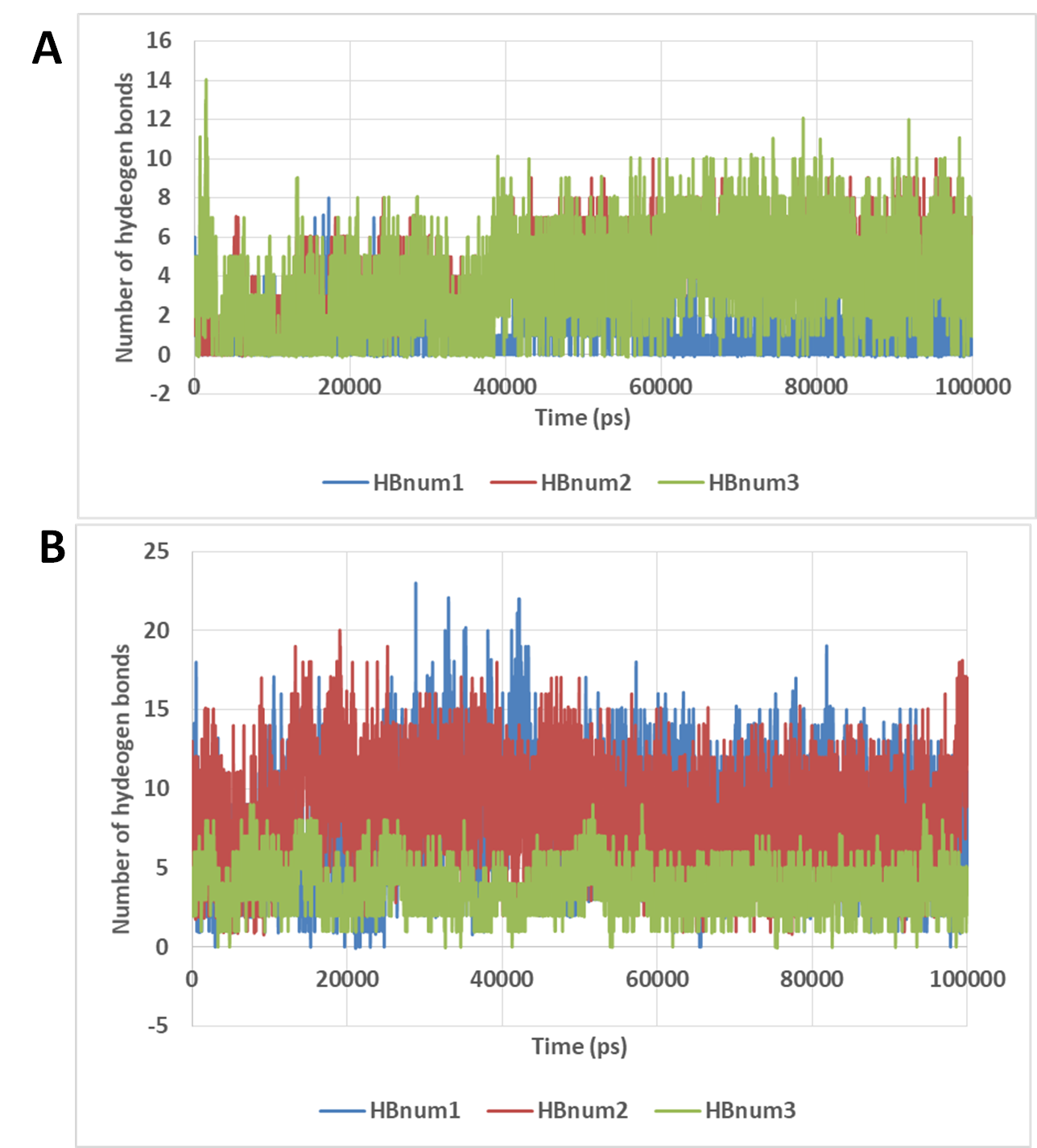

Supplement: Supplemental Information 4 — (A) The number of hydrogen bonds formed by docetaxel (B) The number of hydrogen bonds formed by neohesperidin. The average from the three experiments is displayed in Fig. 7. [file peerj-08-10480-s004.png]

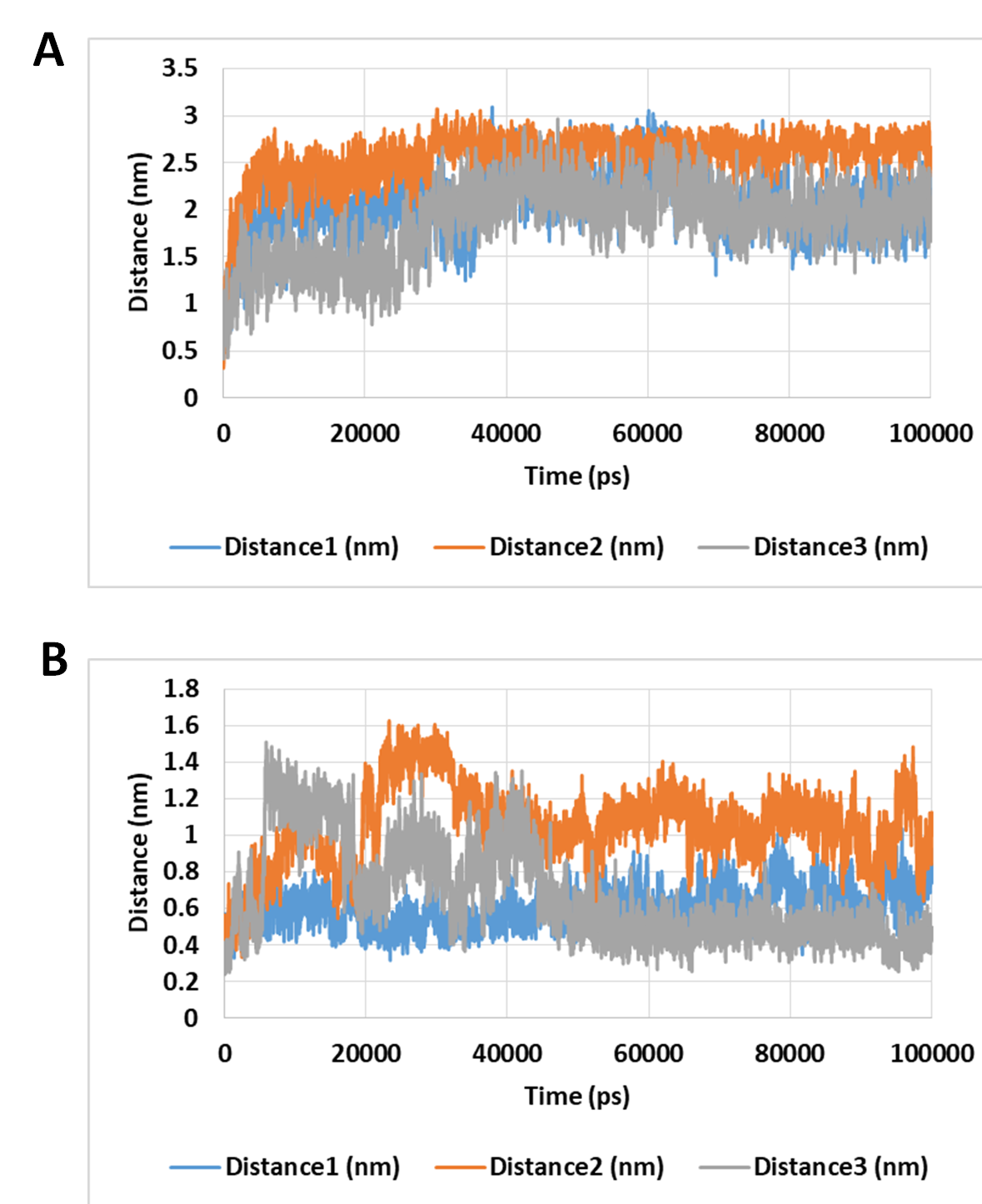

Supplement: Supplemental Information 5 — The selected residues were LYS521 for docetaxel and ASP588 for neohesperidin. [file peerj-08-10480-s005.png]
