# Supplementary material for: Repurposing FDA-approved phytomedicines, natural products, antivirals and cell protectives against SARS-CoV-2 (COVID-19) RNA-dependent RNA polymerase
Source: PeerJ. 2020 Nov 30;8:e10480. doi: 10.7717/peerj.10480 (PMC7713599; doi:10.7717/peerj.10480)
Supplement: Supplemental Information 6 [file peerj-08-10480-s006.docx]

**Supplementary Methods**

**Retrieval of RdRP sequences**

The sequences of RdRP were either retrieved from NCBI GenBank or GISAID (<https://www.gisaid.org/>). The accession numbers were AWH65952.1, P_828869 and YP_009725307 for MERS, SARS and SARS-2 CoVs, respectively.

**Sequence alignment and multiple sequence comparisons**

Sequences were analyzed by pairwise and multiple sequence comparisons. Alignment, sequence identity rates and the number of similarities and differences were analyzed in CLC genomics software (Qiagen Inc., USA).

**Construction of phylogenetic tree**

The neighbor-joining method was used to generate a phylogenetic tree. The tree was generated and edited by CLC genomics workbench software.

**Sequence comparisons**

A comparison between SARS and SARS-2 (COVID-19) CoVs is provided in Figure 1. The highlighted (non-conserved) residues (Fig. S1A) indicate that 34 different residues are present. The percentage identity was 96.35% (Fig. S1B), indicating a high homology rate between SARS and SARS-2 RdRP. In contrast, there was a low homology rate between SARS-2-CoV and MERS-CoV (Fig. S2). There were 267 amino acids that were different, and the percentage identity was lower at 71.44%.


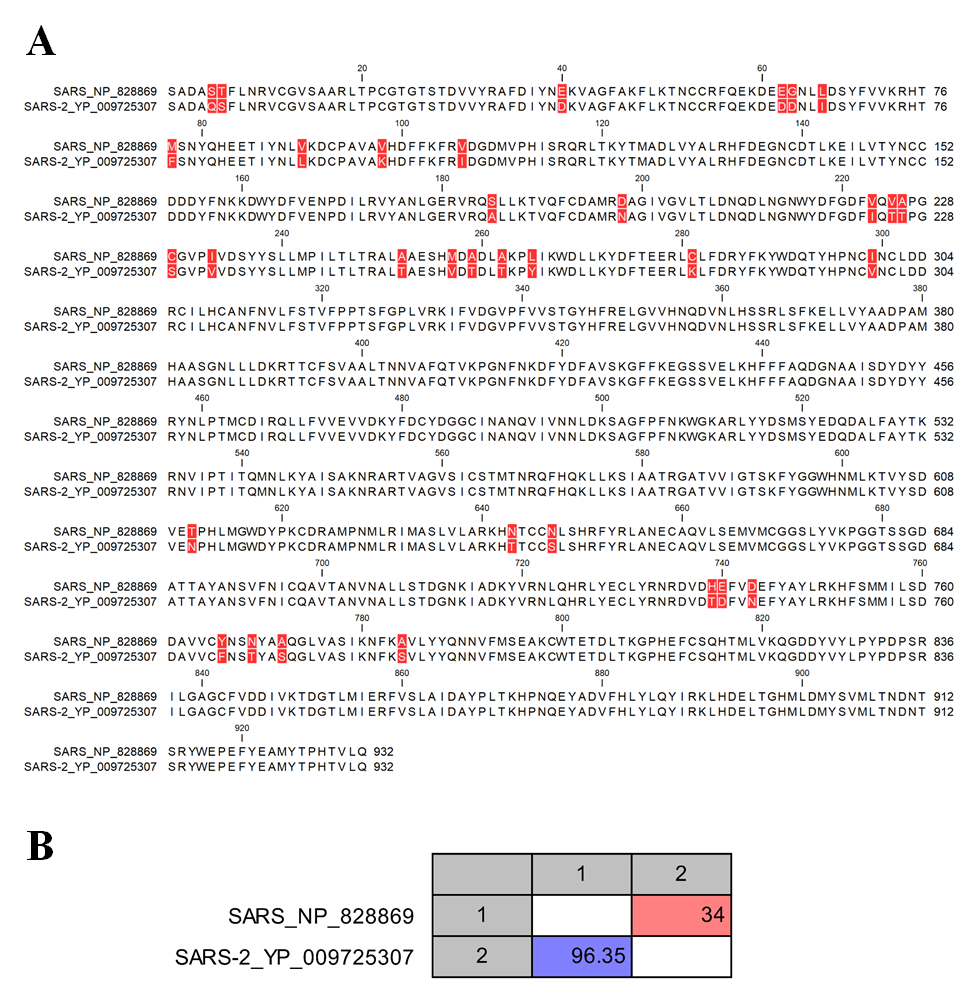


**Fig. S1: Sequence alignment of RdRP from SARS CoV and SARS-CoV-2 (COVID-19). A) Pairwise sequence alignment of SARS CoV and SARS-CoV-2. Conserved residues are not highlighted and displayed in black colour. The different residues are displayed in white and highlighted by a red background colour B) Sequence comparison matrix of COVID-19 and SARS RdRP. The top right cell (in red) shows the number of amino acids differences. The lower left cell (blue) shows the identity%.**


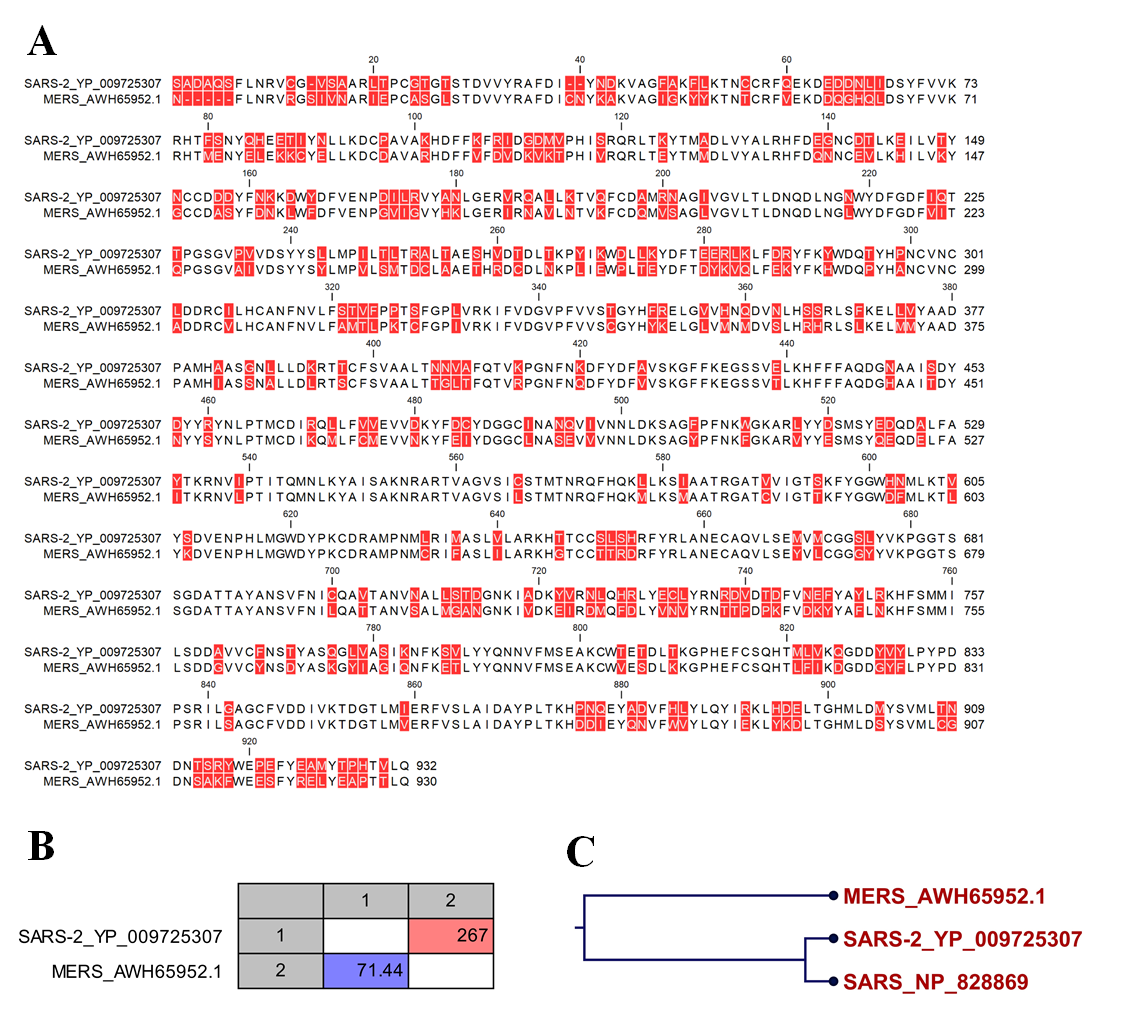


**Fig. S2: Sequence alignment of RdRP from MERS CoV and SARS-CoV-2 (COVID-19). A) Pairwise sequence alignment of MERS CoV and SARS-CoV-2. Conserved residues are not highlighted and displayed in black colour. The different residues are displayed in white and highlighted by a red background colour B) Sequence comparison matrix of COVID-19 and MERS CoV RdRP. The top right cell (in red) shows the number of amino acids differences. The lower left cell (blue) shows the identity%.**
